# Pilot deployment of beta carotene-enriched rice (Golden Rice) in the Philippines

**DOI:** 10.1038/s41598-026-48565-5

**Published:** 2026-05-04

**Authors:** Ronan G. Zagado, Marissa V. Romero, Jesusa C. Beltran, Fidela P. Bongat, Reynante L. Ordonio, Joy Bartolome A. Duldulao, Anielyn Y. Alibuyog, Victoria C. Lapitan, Albert Christian S. Suñer, Gerardo F. Estoy, Mary Ann U. Baradi, Sailila E. Abdula, Ommal H. Abdulkadil, Rhemilyn Z. Relado-Sevilla, Raul M. Boncodin, Ma. Aileen A. Garcia, Ellen E. Villate, Russell F. Reinke

**Affiliations:** 1 Department of Agriculture - Philippine Rice Research Institute (DA-PhilRice), Maligaya, Science City of Muñoz, Nueva Ecija 3119 Philippines; 2 International Rice Research Institute (IRRI), Los Baños, Laguna 4030 Philippines; 3 Biotechnology Coalition of the Philippines (BCP), 47 Kalayaan Ave, Diliman, Metro Manila, Quezon City, 1101 Philippines; 4 Present Address: Central Luzon State University (CLSU), Science City of Muñoz, 3119, Nueva Ecija Philippines

**Keywords:** Beta carotene, Biofortified rice, Golden Rice, Malusog Rice, Vitamin A deficiency, Biological techniques, Biotechnology, Genetics, Plant sciences

## Abstract

Golden Rice, registered as Malusog Rice in the Philippines, is a biofortified rice containing beta carotene in its grains to address vitamin A deficiency (VAD), a prevalent public health problem. It can provide 30–50% of the estimated average requirement for vitamin A. Its commercial approval in July 2021 led to a pilot-scale deployment in selected high VAD prevalent areas. From 2022 wet cropping season to 2023 wet cropping season, it was cultivated on 202 hectares across 24 provinces in the country, demonstrating significant field performance comparable to that of other rice varieties. It gained an average yield of 3.9t/ha and a maximum yield of 8.3t/ha. Over 77 tons of Malusog Rice were distributed to its target VAD beneficiaries, while eight tons were allocated for market testing. Survey results show that 97% of the target VAD beneficiaries expressed their desire to include Malusog Rice in their diet when it becomes widely available. Moreover, market testing indicates that Malusog Rice was found to be a marketable product, with a price comparable to regular/well-milled rice. The milestone approval and deployment of Malusog Rice in the Philippines position the country as a global leader in agricultural biotechnology, being the first to approve and deploy genetically engineered rice with nutritional benefits.

**Supplementary Information:**

The online version contains supplementary material available at 10.1038/s41598-026-48565-5.

## Introduction

Golden Rice, registered as Malusog Rice (‘Malusog’ meaning healthy in Filipino) in the Philippines, is a biofortified rice that contains beta carotene (a source of vitamin A), giving its grains a golden color. Study shows that beta-carotene from biofortified crops can significantly enhance vitamin A intake and improve serum retinol concentrations^1^. A cup of cooked Malusog Rice can provide 30–50% of the estimated average requirement of vitamin A for under-five children and pregnant or lactating mothers^2 ^(See Supplementary Document 1 for the methodology in computing how much vitamin A can be provided by Malusog Rice; the same calculations are detailed elsewhere^3^. In the Philippines, vitamin A deficiency (VAD) remains a significant public health issue affecting 15.5% percent or 2 million Filipino children under the age of 5 and poses risks to pregnant and lactating women^4^. Despite efforts such as food fortification, vitamin A capsule supplementation, promotion of optimal breastfeeding and complementary feeding, dietary diversification, and nutrition education, the prevalence of VAD has not significantly improved in the past decade. Based on the 2018–2019 Expanded National Nutrition Survey (ENNS), 23 out of 79 areas reported severe VAD levels^4^.

Professors Ingo Potrykus and Peter Beyer developed Golden Rice using genetic engineering, incorporating genes from corn and a soil microorganism to produce beta carotene in the grain^5,6^. This beta carotene is the same as that found in green leafy and yellow-colored fruits and vegetables. With licenses from the co-inventors, Golden Rice technology was donated for humanitarian reasons^7^. The inventors intended it to be available in developing countries as a public good for resource-poor farmers^8,9^, allowing them to save seeds for future plantings, at no greater cost than white rice and with no use limitations except export^7^.

The Department of Agriculture-Philippine Rice Research Institute (DA-PhilRice) leads its development and deployment in the Philippines in partnership with the International Rice Research Institute (IRRI). Following the approval of the commercial propagation, DA-PhilRice together with IRRI launched the pilot-scale deployment of Malusog Rice in selected areas.

This paper is a documentation of the experience of Malusog Rice pilot-scale deployment in the Philippines from 2022 to 2023. It specifically describes the extent of the deployment in terms of (1) Supply (ensuring availability and accessibility of quality Malusog Rice seeds and milled rice); (2) Demand Creation (increasing knowledge, promoting acceptability, and encouraging desirable behaviors for uptake); and (3) Governance (creating a supportive, science-based policy and enabling environment and governing structures for the smooth deployment of Malusog Rice in target areas). Most importantly, this paper presents the acceptability of Malusog Rice among farmers, retailers, and consumers. Moreover, it draws insights on the Malusog Rice scalability and sustainability.

## Methods

### Study design

**Fig. 1:**
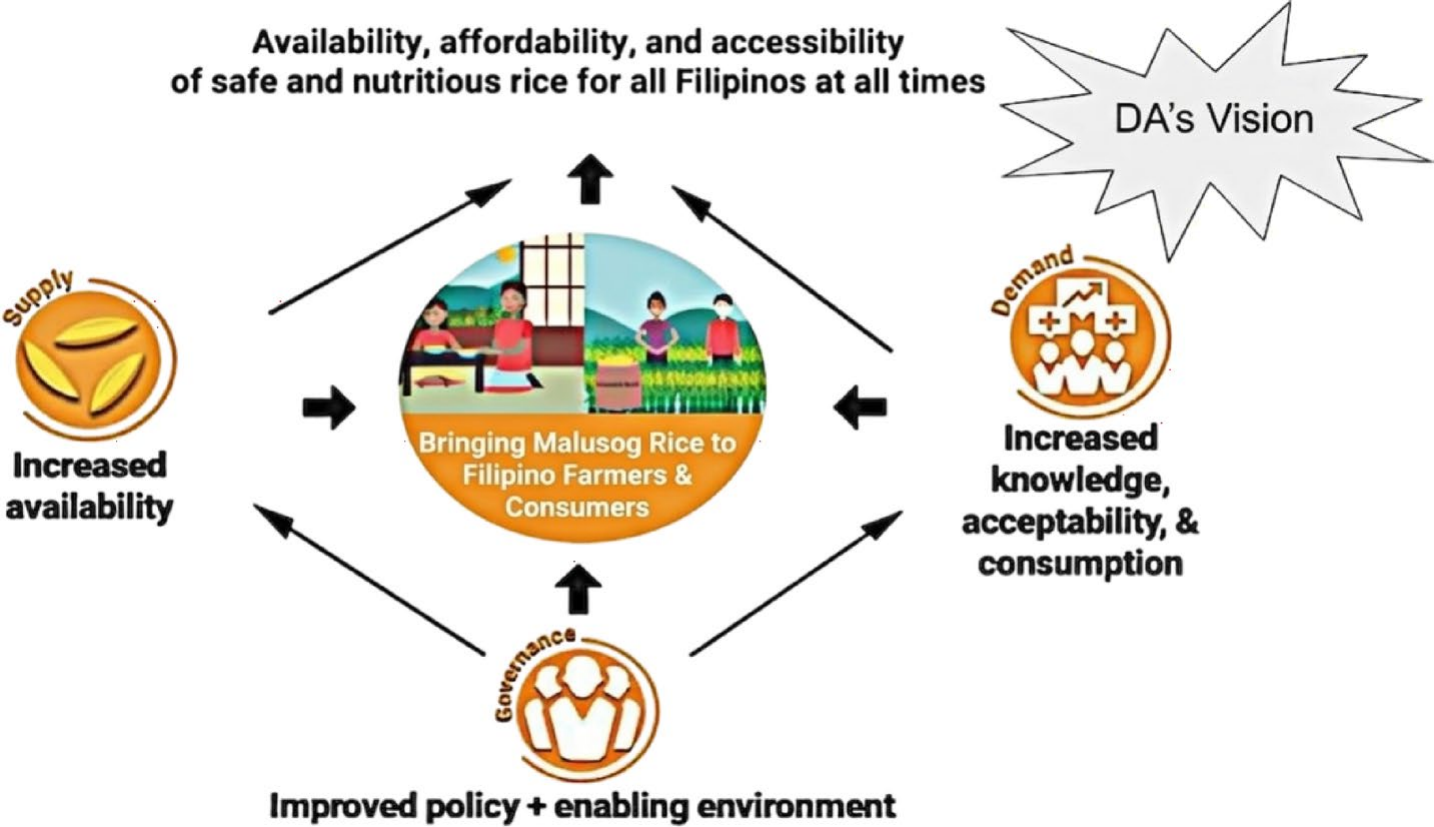
Deployment framework for Malusog Rice showing interrelationship of supply, demand, and governance in support of the Philippine Department of Agriculture’s vision for safe and nutritious rice for all Filipinos.

Conducted from 2022 to 2023, this study employed an exploratory pilot deployment design to document processes, product field performance, observations, and stakeholder feedback during the initial rollout of Malusog Rice. The pilot framework was structured around three interrelated components: supply, demand, and advocacy/governance (Fig. 1). Supply creation focused on ensuring seed availability through on‑farm production. It involved (1) seed production, (2) on-farm production in selected areas, and (3) conduct of briefings/training. Since Malusog Rice is a genetically modified product, it required stringent seed quality management to maintain high-quality seeds. To develop more varieties of Malusog Rice, DA-PhilRice also continued to introgress the Malusog Rice trait into other popular inbred varieties.

Demand generation aimed to build knowledge and positive receptivity in target communities. This component facilitated product positioning and simulated demand creation for Malusog Rice. The Malusog Rice Program in close collaboration with partners implemented (1) product management, (2) acceptability and market testing, and (3) education and communication. These activities employed strategic planning and execution that involved deliberate messaging, use of appropriate channels, and targeted outreach to stakeholders.

Policy advocacy and stakeholder outreach were undertaken. Policy advocacy refers to organized initiatives that seek to secure support of policymakers and program implementers for Malusog Rice through the passage of resolutions, position statements, or other forms of endorsement. Stakeholder outreach was carried out through courtesy calls, one-on-one meetings, information sessions, council presentations, as well as local media outreach. The team consistently communicated with stakeholders, allies, and the general public at multiple levels (national, regional, and local) to create an enabling environment and solicit support for the technology.

The overarching goal was to establish a system for continued production and distribution until Malusog Rice becomes widely accepted, cultivated, and consumed. In the long term, the strategy envisions Malusog Rice as economically sustainable—providing profitable income for farmers while enhancing the nutritional quality of Filipino diets.

### Study setting and population

The pilot deployment of Malusog Rice covered 24 provinces, comprising the 7 initial provinces and the additional 17 provinces included based on the willingness of local government units (LGUs) to participate in the deployment. The participating provinces were selected based on three criteria: (1) provincial stunting data (used as a proxy for poor nutrition) obtained from the 2015 National Nutrition Survey of the Department of Science and Technology-Food and Nutrition Research Institute (DOST‑FNRI), (2) designation as a priority province under the Philippine Plan of Action for Nutrition (PPAN) 2017–2022 with stunting rates above 40%, and (3) quantity of rice production per capita. Additional considerations included the absence of ordinances prohibiting GMO cultivation, peace and order conditions, strong LGU commitment, and the presence of local champions to facilitate consumer acceptance and integration with existing nutrition programs. The initial seven provinces were: Quirino, Antique, Agusan del Sur, Maguindanao, Catanduanes, Western Samar, and Lanao del Norte. The additional 17 provinces joined the pilot following engagement with LGU officials who expressed interest in adopting Malusog Rice within their agriculture and nutrition programs. These provinces included Albay, Batangas, Bukidnon, Capiz, Leyte, Cagayan, Dinagat Islands, Ilocos Norte, Iloilo, Isabela, Laguna, North Cotabato, Nueva Ecija, Pangasinan, South Cotabato, Surigao del Sur, and Sultan Kudarat.

The study population comprised stakeholders, including farmers, households vulnerable to Vitamin A Deficiency (VAD), relevant agencies, LGUs, and market players across the aforementioned provinces.

#### Sample size and recruitment

Participants in the pilot deployment were selected using defined criteria. For on-farm production, farmer cooperators were drawn from provinces that expressed willingness to grow Malusog Rice. Willingness was included as a criterion for two reasons: (1) certain areas prohibit GMO cultivation, making recruitment of unwilling or legally restricted farming communities infeasible and ethically inappropriate; and (2) alignment with study objectives, as without willingness the product could not be trialed, and meaningful evaluation would not have been possible. While necessary to ensure feasibility and compliance, this criterion may introduce selection bias and limit generalizability, which we have acknowledged as a study limitation. For the distribution and acceptability survey, households vulnerable to VAD—specifically those with lactating women and children aged 6–59 months—were recruited in the seven initial target provinces. In each of the seven initial provinces, 100 households were selected (50 with lactating women and 50 with children aged 6–59 months), yielding 700 identified respondents, of whom 694 ultimately participated in the acceptability survey. Surveys were conducted in VAD-deficient populations because Malusog Rice is designed to complement existing interventions addressing vitamin A deficiency. These settings provided representative environments for rollout and practical insights for deployment strategies. However, focusing on VAD-deficient groups may introduce selection bias and limit the generalizability of findings to the wider Philippine population. Eligible beneficiaries were identified by DA‑PhilRice in close coordination with the LGUs, particularly the provincial and municipal health offices and the respective nutrition committees/councils. For the market study, 14 market retailers across the seven pilot provinces and three highly commercial provinces were engaged to provide insights into the product’s marketability. The partner-retailers were selected based on being active rice retailers in the town’s public market. Retailers were briefed beforehand and only those who expressed willingness to participate were included, ensuring feasibility of the market test and reliable feedback from retailers capable of handling the product at scale. For policy advocacy, stakeholders with interest and influence from local, regional, and national levels were reached through structured engagement activities to secure governance support and integration of Malusog Rice into agriculture and nutrition programs.

### Data collection

Various data collection methods were employed. For on‑farm production, monitoring logs were maintained to capture field data such as yield and production costs. For feedback about Malusog Rice on-farm cultivation, secondary data, such as farmer testimonies, were drawn from existing news stories published in the project newsletter available on the project’s official webpage at https://www.philrice.gov.ph/golden-rice/golden-rice-e-newsletter/. For quality control purposes, grain quality evaluation was conducted at the PhilRice Grain Quality Laboratory to determine the carotenoid content of Malusog Rice harvest samples after two months of storage as rough rice (i.e., as whole grains with the hulls intact) to give a realistic estimate of provitamin A availability at the point of consumption. Samples were transported from the deployment production sites, where rough rice was stored at ambient conditions in plastic sacks. Upon receipt, the rice was milled and placed in paper envelopes at refrigerated temperature until analysis. From one bag of rough rice, three samples were obtained, which were milled and processed for grain quality and carotenoid analysis, resulting in triplicates. As these analyses were conducted for monitoring purposes, mean values are shown, and no statistical analysis was performed. All storage and handling of the samples followed the same protocols as for ordinary rice. A survey was administered among target VAD households to assess their willingness to integrate Malusog Rice into their diet, while a sensory evaluation measured its storage, cooking, and eating qualities. In the market test, each retailer was given 500 kg of Malusog Rice for one week of sales. Malusog Rice was sold by partner retailers at prices comparable to regular/well‑milled rice, and sales, selling prices, purchased volumes (including repurchases), and restock volumes were observed under real market conditions. The objective with the limited quantities available was to assess “marketability” and not the actual market penetration of the product. Communication and stakeholder engagement metrics were also monitored, including the number of engagements conducted, types of stakeholders reached, news articles published, and outcomes achieved. Documentation of activities as well as stakeholder’s testimonies was done through news articles published on the project’s official webpage. While this pilot demonstrated strong communication and stakeholder engagement activities with complete recording of activities, a limitation is that no formal pre- and post- assessment of knowledge was conducted. Future studies should include systematic knowledge assessments to strengthen the evidence of impact. Challenges encountered during the pilot deployment were documented and immediately addressed to avoid disruption of the implementation. However, the effectiveness of the strategies employed was not systematically assessed, and it is therefore proposed that future studies should include such evaluation.

### Data analysis

The data analysis was primarily descriptive in nature. On‑farm production of Malusog Rice was analyzed in terms of minimum and maximum yield, as well as production cost per hectare per cropping season. To enrich the findings and provide contextual perspectives on these production data, relevant farmer testimonies drawn from the project newsletter were cited in the Result section. To measure the total carotenoid content of Malusog Rice, a spectrophotometer was used. It is important to note that the carotenoid measurements were conducted for product quality monitoring, and that all the detailed statistical analysis of carotenoid content across seasons and locations is reported in Swamy et al., (2021). Results of the acceptability survey were summarized as percentages across categories of responses. Market test data were described in terms of selling price (in pesos, converted to USD, with each peso equivalent to $0.02 USD), purchased volume (in kilograms), and restock volume (in bags). For social marketing and communication of the deployment, the data analysis was purely descriptive, focusing on the summarization and presentation of activities, outputs, and stakeholder feedback. This approach was intended to document the implementation, rather than to draw statistical inferences.

### Compliance 

The research and deployment activities involving Malusog Rice were conducted in accordance with implementing guidelines and protocols approved by the Philippine Rice Research Institute (PhilRice) and the International Rice Research Institute (IRRI). Activities were also carried out in compliance with national biosafety regulations, and all necessary approvals were obtained from the Philippine Department of Agriculture – Bureau of Plant Industry (DA-BPI). Field trials were conducted under Biosafety Permit No. 19 − 001, issued on May 20, 2019 (See Supplementary Document 2 for the biosafety permit for field trials). Approval for direct use as food, feed, and for processing (FFP) was granted under Permit No. 19-060FFP, issued on December 19, 2019 (See Supplementary Document 3 for the biosafety permit for FFP). Commercial propagation was authorized through Biosafety Permit No. 21-012Propa, issued on July 21, 2021 (See Supplementary Document 4 for the biosafety permit for commercial propagation). The Philippines is the first country in the world to approve Malusog Rice for propagation^10^. Not only that, the product has also been assessed as safe as ordinary rice by Food Standards Australia New Zealand (FSANZ)^11^, Health Canada^12^, and United States Food and Drug Administration (US FDA)^13^(See Supplementary Documents 5, 6, and 7 for the biosafety approvals from FSANZ, US-FDA, and Health Canada).

Prior to commercial propagation, Golden Rice underwent confined testing under a Confined Test Permit (2015 − 0286) issued by the DOST Biosafety Committee, following national biosafety guidelines (See Supplementary Document 8 for the confined testing approval). Moreover, in accordance with national biosafety regulations, field trials were conducted with the formal consent of the concerned local government units, as documented through legislative resolutions endorsing the activity. Golden Rice in the background of PSB Rc 82 was also registered with the National Seed Industry Council (NSIC) as NSIC 2022 Rc 682GR2E or Malusog 1; hence, the shift from Golden Rice to Malusog Rice (See Supplementary Document 9 for the varietal registration certification).

An ethical review certificate of exemption for the study on market and consumer acceptance of Malusog Rice was granted by the Single Joint Research Ethics Board of the Department of Health on October 30, 2020 (See Supplementary Document 10 for the SJREB ethical review certificate of exemption for Malusog Rice Market Study). For subsequent assessments of Malusog Rice’s acceptability, ethical approval was obtained from the International Rice Research Institute (IRRI) on December 12, 2022, under Protocol Code Number 2022-0016-A-2022-14 (See Supplementary Document 11 for the IRRI Research Ethics Committee Approval Form). Informed consent was obtained from all participants involved in the acceptability studies.

## Results

### SUPPLY: Ensuring availability and access to Malusog Rice

Following the approval of the biosafety permit for its commercial propagation, Malusog Rice underwent seed increase on 1.33 ha at DA-PhilRice [1.13 ha in PhilRice Central Experiment Station and 0.20 ha in PhilRice Isabela] during the 2022 dry cropping season with a total seed produce of 3.78 tons. The seed produced was then distributed beginning May 2022 for the first-ever on-farm cultivation of Malusog Rice in selected DA-PhilRice branch stations, DA research stations, and farms of partner farmers and seed growers. Before releasing the seeds for planting, genotyping was performed to ensure that these were of the highest quality (95–99% purity). Nucleus seeds produced during the 2023 dry season had 99.8% purity, while the 2023 WS harvest had 99.6%. In terms of grain quality, the average total milling recovery was around normal 65.0% and they achieved acceptable physical attributes and physicochemical properties.

**Fig. 2:**
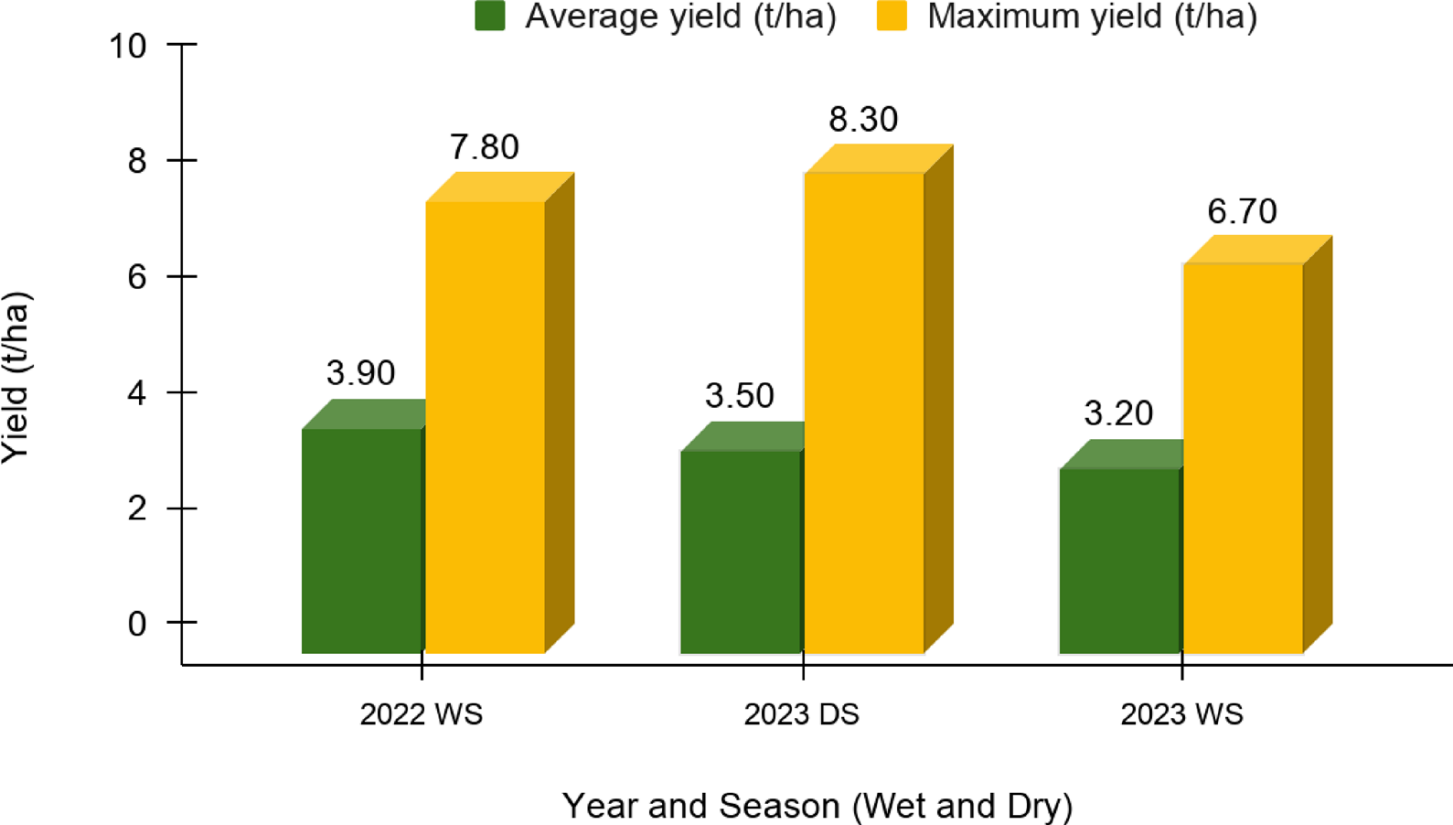
Average and maximum yields (tons per hectare) of Malusog Rice recorded across three farm‑level production seasons—2022 Wet Cropping Season (WS), 2023 Dry Cropping Season (DS), and 2023 Wet Cropping Season (WS) —as part of the pilot product deployment.

**Fig. 3:**
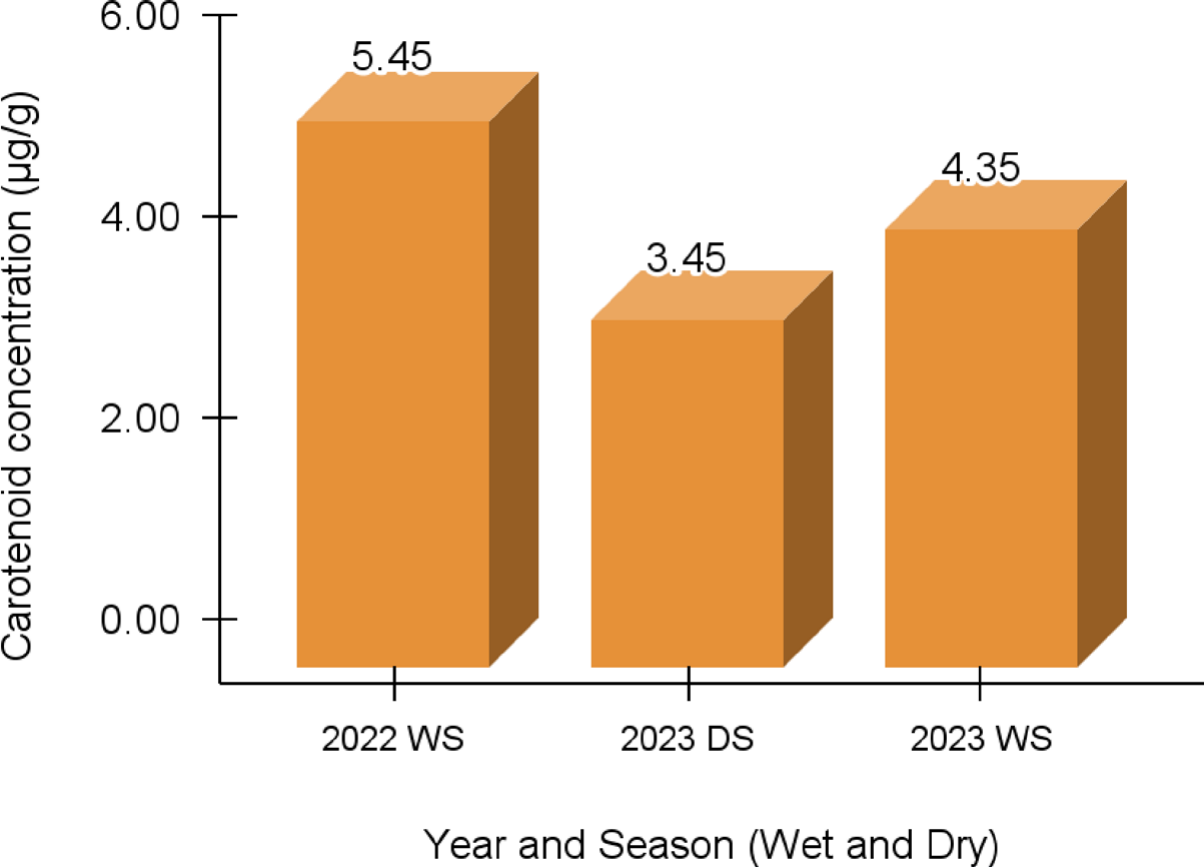
Mean carotenoid levels two months after harvest of milled Malusog Rice samples collected across cropping seasons in the Philippines.

In 2022 wet season (WS), Malusog Rice production was established for the first time at the farm level on 38.15 hectares across 17 provinces in the country, with an average yield of 3.9t/ha and a maximum yield of 7.8t/ha. By 2023 dry season (DS), 47.09 hectares were established across 17 provinces with an average yield of 3.5t/ha and a maximum yield of 8.3t/ha. By 2023 wet season (WS), production expanded to 117 hectares across 18 provinces with an average yield of 3.2t/ha and a maximum yield of 6.7t/ha (Fig. 2). The carotenoid content of on-farm harvests was measured and analyzed, with average values ranging from 3.45 to 5.45 *µ*g/g in milled rice (equivalent to ~ 2.5 *µ*g/g in cooked rice), as shown in Fig. 3. At these concentrations, Malusog Rice can contribute at least 30% of the Estimated Average Requirement (EAR) for vitamin A in children (See Supplementary Document 1 for the methodology in computing how vitamin A can be provided by Malusog Rice). This level of contribution is considered adequate, as Golden Rice is intended to complement other dietary sources of vitamin A, such as vegetables, fruits, and fortified foods.

Meanwhile, production costs during the 2022 wet season were collected from 16 sampling sites (PhilRice Bicol − 2, PhilRice Isabela − 4, PhilRice Midsayap − 6, and PhilRice Negros − 4). They served as the initial deployment locations for Malusog Rice at the farm level. Total production cost was US$1,015 (Php57,864.38) per hectare, resulting in a mean cost of US$0.213 (Php12.15) per kilogram. In the 2023 dry season, production costs were recorded at US$1,058 (Php60,302.14) per hectare with a mean cost of US$0.211 (Php12.07) per kilogram. Data were gathered from 20 sampling sites (PhilRice Bicol − 7, PhilRice Isabela − 6, PhilRice Midsayap − 4, and PhilRice Agusan − 3).

Notably, farmers reported that Malusog Rice production cost is comparable with the existing popular inbred rice varieties, using the same crop management practices and inputs (See Supplementary Document 12 for farmers’ testimonies, as reported in the Malusog Rice newsletter).

### Continuous breeding work

**Fig. 4:**
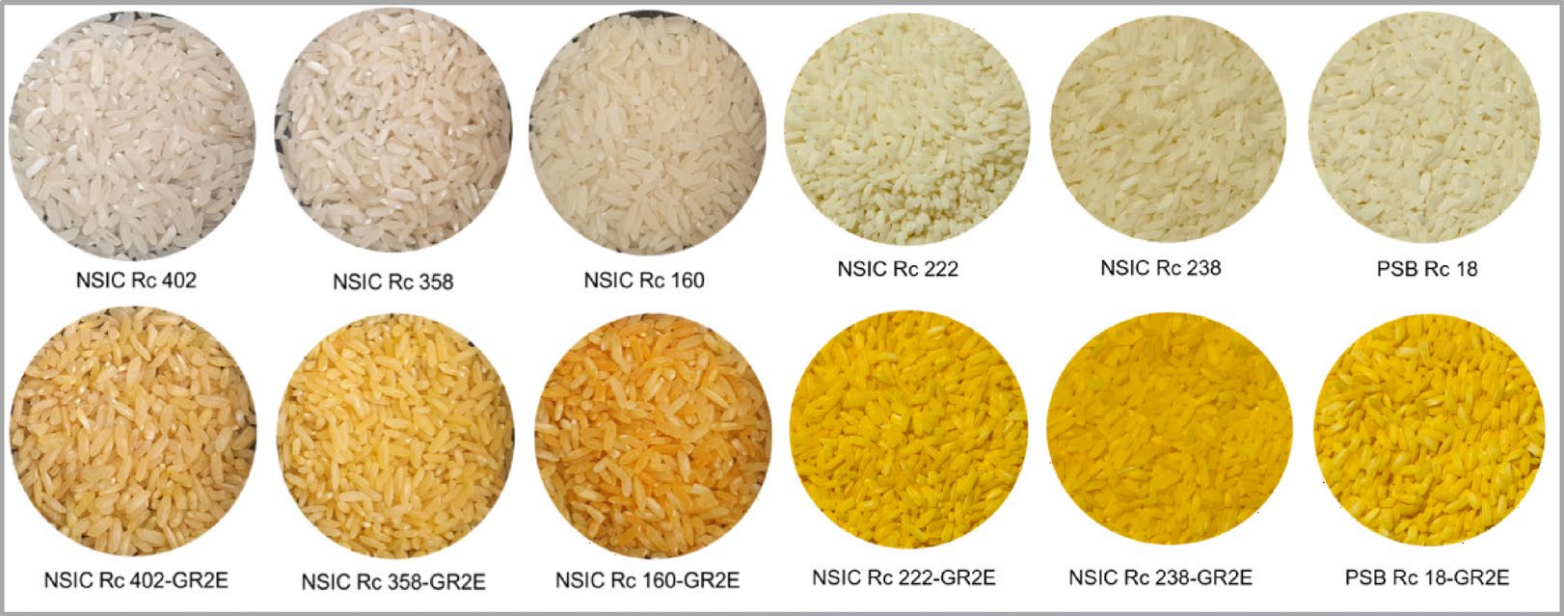
Six Malusog Rice lines shown alongside their near‑isogenic comparators.

Breeding activities continued to transfer the Malusog Rice trait into other popular, high-yielding local inbred rice varieties leading to the development of 23 introgression lines. Six lines in the backgrounds of NSIC Rc 402, Rc 358, Rc 160, Rc 222, Rc 238, and PSB Rc 18 were selected as candidates for varietal registration (Fig. 4). Developed through introgression (a breeding technique that transfers beta‑carotene genetic trait into other popular, high‑yielding rice varieties in the Philippines), these lines will undergo multi‑location trials, including distinctiveness, uniformity, and stability (DUS) testing, to generate agronomic and grain quality data required for varietal registration.

### DEMAND: Raising awareness and creating demand for Malusog Rice

#### Social marketing and communication activities

A targeted communication and marketing strategy was designed and implemented, reaching out to the program’s main stakeholders: farmers, consumers, and intermediaries (i.e., LGUs, policy makers, media/information network) in the pilot provinces. The team undertook efforts to enhance the stakeholders’ awareness and promote consumer demand for Malusog Rice using the following communication and marketing strategies:

##### Branding and packaging 

 To develop a strong brand presence, a brand playbook was created to provide guidance on Malusog Rice brand elements, including, but not limited to, its prescribed logo, color palette, imagery, typography, and voice (See Supplementary Document 13 for the brand playbook). All branding and packaging for Malusog Rice seeds, milled rice, and related marketing collateral were developed consistent with the guidelines set in the brand playbook.

##### Knowledge products and marketing collateral 

Aligned with the branding guidelines, knowledge products and marketing collateral were developed (e.g. posters, banners, videos, radio plugs, flyers, brochures, etc.), distributed in various formats, and translated into local languages as needed (See Supplementary Document 14 for the samples of posters, banners, and other information materials). In 2022, 26 new/updated knowledge products were produced and circulated to 4,839 stakeholders. In 2023, 35 knowledge products and marketing collateral were distributed to 14,450 stakeholders. Sampling booths, campaign shirts, bucket hats, tote bags, and diary planners were also provided. A monthly e-newsletter sent to 914 stakeholders to update about the development and deployment of Malusog Rice in the Philippines gained 90% client satisfaction rating.

##### Technology demonstrations, farm walks, and harvest festivals

The field performance of Malusog Rice was showcased through farm walks, harvest festivals, and technology demonstrations, where farmers and other stakeholders were invited to visit rice fields of partner-farmers, providing stakeholders with firsthand experience on how Malusog Rice performs in the field. In 2022, six farm walks/harvest festivals were organized while 14 farm walks and 21 techno demo farms in 2023 allowing farmer-cooperators to share experiences in planting Malusog Rice. Farmers recognized its similarity to ordinary rice in terms of management and field performance (See Supplementary Document 15 for the farm walk documentation, as reported in the Malusog Rice newsletter).

##### Participation in international and local scientific and external milestone events

The Malusog Rice Program aimed to increase visibility, product awareness, and engagement with stakeholders by participating in global, national, and local activities (e.g., conventions and exhibits, workshops, boot camps, webinars, etc.). The program was presented at several global events (The Micronutrient Forum, International Rice Congress) and national initiatives related to agriculture, nutrition and health, (Nutrition Month, National Biotechnology Week, National Rice Awareness Month, World Food Day, and other key events on the United Nations calendar) and local milestone celebrations (cultural festivals, community events). In 2022, the program participated in 11 national and local events (Farmers’ and Fisherfolks’ Month in May), Nutrition Month in July), National Rice Awareness Month, and National Biotechnology Week in November), 18 conferences/webinars and organized four exhibits and two cooking contests. In 2023, the program engaged in 20 webinars/symposia/conferences both local and international and participated in 55 exhibits and trade fairs to introduce the product to target audiences. Notably, DA-PhilRice won the Best Paper Award (1st Place) at the Crop Science Society of the Philippines scientific conference.

##### Ramping up information campaigns through network mobilization

The Malusog Rice Program successfully engaged communication and information officers from various organizations (DA-Regional Field Offices, Philippine Information Agency, Agricultural Training Institute, and LGU information offices) through a workshop in August 2022. This resulted in 7 radio interviews and 9 press releases in the target provinces (See Supplementary Document 16 for the list of press releases on Malusog Rice). Additionally, a Facebook group was established to facilitate information exchange and content vetting. In July 2023, the program received the BINHI Award for Best Agri-related Advocacy Campaign from the Philippine Agricultural Journalists, Inc. (PAJ).

##### Media outreach and news monitoring

**Fig. 5:**
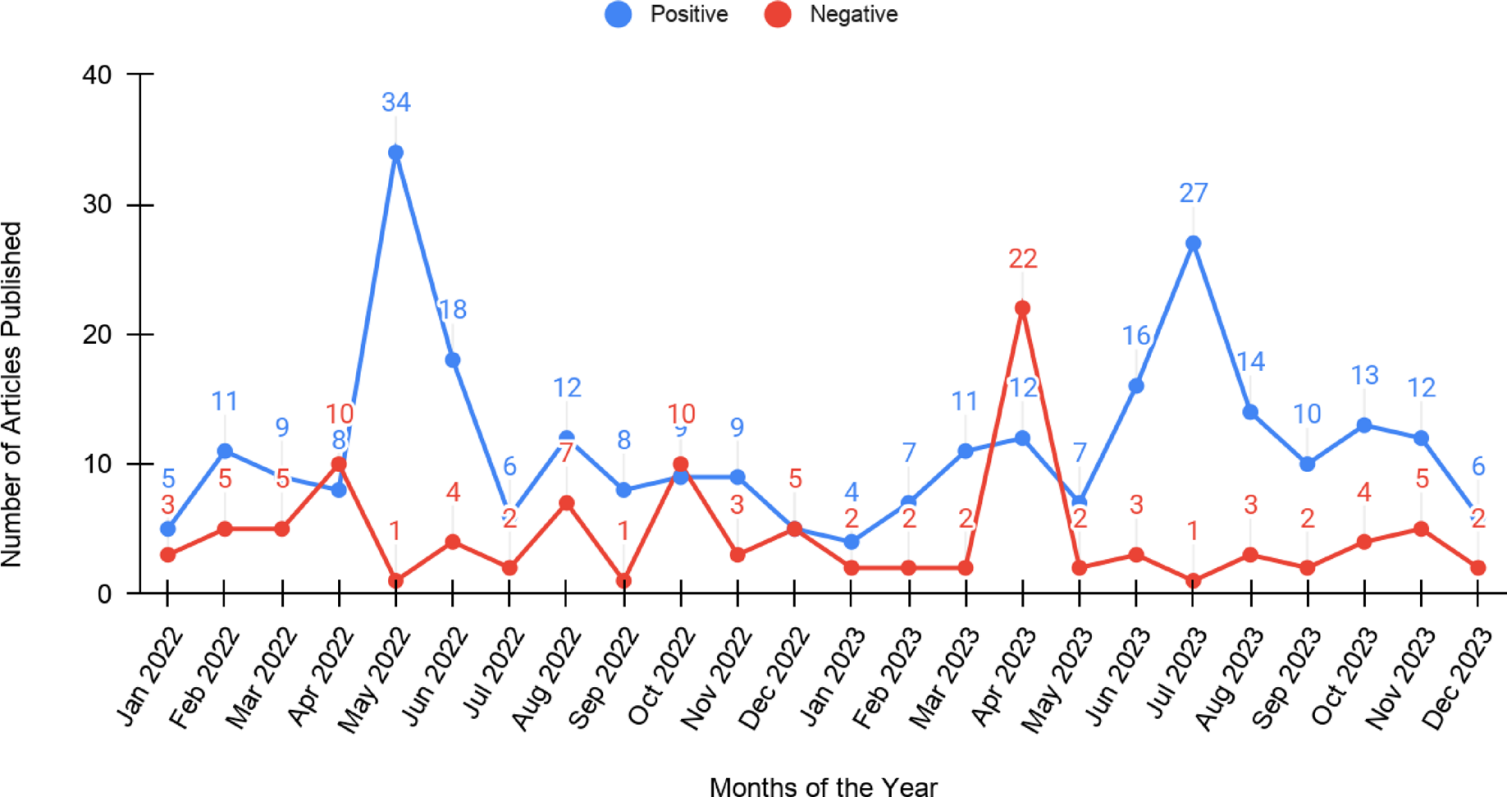
Tone analysis (positive vs. negative) of press releases about Malusog Rice, based on 379 published articles, showing that 70% had a positive tone while 28% had a negative tone.

**Fig. 6:**
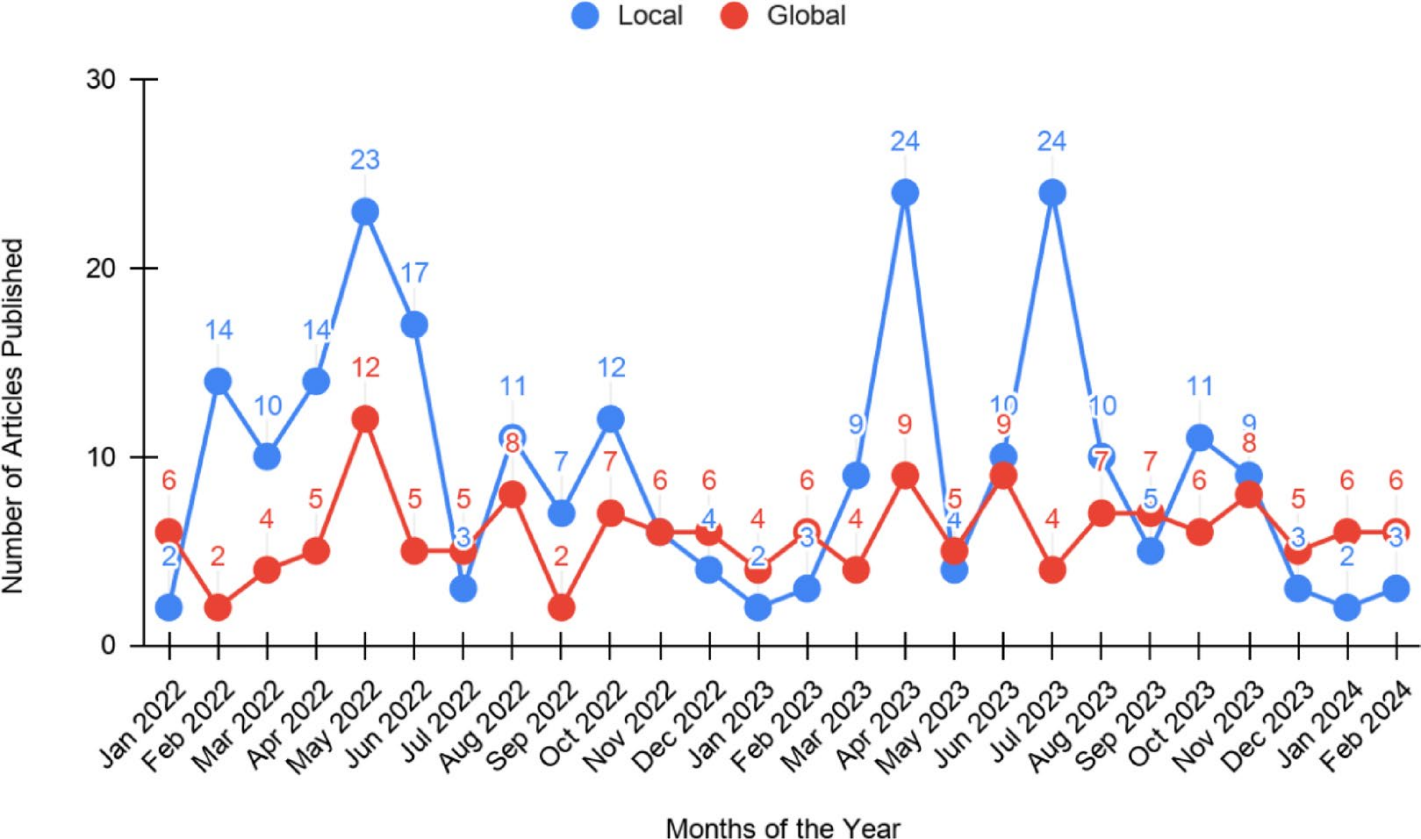
Distribution of press releases by scope (global vs. local), showing that approximately 63% originated from local outlets and 37% from global outlets.

 The Malusog Rice Program implemented a comprehensive media outreach strategy to promote product visibility and deliver key messages. This includes fostering relationships with local journalists and broadcasters. In 2022, local media briefings held in the pilot provinces resulted in18 radio broadcasts, including live interviews and news on Malusog Rice. In 2023, 30 public service announcements and 11 radio interviews were aired. Local media outlets published 10 stories (from feature stories to coverage of events and field activities). In 2022 and 2023, media monitoring revealed that out of 379 published articles, 70% had a positive tone, while 28% had a negative tone (Fig. 5). Around 63% of the news came from local outlets, while 37% came from global outlets, indicating that on-the-ground activities are more likely to attract media attention (Fig. 6). Proactive outreach, including interviews, technical briefings, and taste-testing, effectively communicated the objectives and activities related to the pilot-scale deployment of Malusog Rice to the local community. Additionally, the Malusog Rice Communication Team ensured that spokespersons were well-prepared for media engagements through refresher media training and the development of talking points.

##### Social media presence

Social media was used to amplify messages and facilitate engagement. Online presence was maintained to provide up-to-date information on Malusog Rice, frequently asked questions (FAQs), key resources, and program updates (https://www.philrice.gov.ph/golden-rice/about-gr/). In 2022, the program’s microsite on the DA-PhilRice official webpage garnered 4,283 page views, while its social media accounts on Facebook (https://www.facebook.com/MalusogRice), Twitter (https://x.com/MalusogRice), and Instagram (https://www.instagram.com/malusogrice/) reached a combined total of 1,118,967 people with 57,473 engagements. Collaborative efforts with local partners, pages of DA-PhilRice branch stations, regional government agencies, LGUs, and other organizations led to the posting of various content, including a video feature highlighting the first harvest of Malusog Rice in Antique. In 2023, the program’s social media campaign resulted in 26,736 total engagements and 433,906 total audience reach.

##### Recipe and product development

The Malusog Rice Program collaborated with the Center for Culinary Arts, Manila, to create recipes using Malusog Rice as the main ingredient. Twenty (20) recipes were developed showcasing Malusog Rice’s versatility by using various indigenous ingredients, cooking methods, and heirloom techniques from the Philippines (See Supplementary Document 17 for the Malusog Rice recipes). Cooking festivals were held in the target sites to encourage women/mothers to develop their recipes using Malusog Rice. Stakeholders noted that Malusog Rice is ‘tasteful’, ‘smells like ordinary well-milled rice’, and ‘does not have any distinct smell’ (See Supplementary Document 18 for the stakeholder’s feedback on the sensory characteristics of Malusog Rice).

### Distribution of Malusog Rice to VAD households

Malusog Rice contains enhanced levels of beta-carotene, a precursor of Vitamin A, which contributes to improved dietary intake among vulnerable households (Fig. 3). ​​A total of 3,500 kg of milled Malusog Rice was distributed to 700 households at risk of VAD in Quirino, Catanduanes, Antique, Samar, Agusan del Sur, Lanao del Norte, and Maguindanao. Each household received 5 kg of Malusog Rice. In addition to these recipients, 605 households were provided Malusog Rice during promotional activities. Altogether, the program served 1,305 households out of a target population of 1,000,000 (Table 4). It also covered 41% (14/34) of the priority provinces of the Philippine Plan of Action for Nutrition (PPAN). The goal is to have Malusog Rice comprise 10% of the total rice production in the Philippines, enough to meet the rice requirement of at least 50% of the VAD households in the country within the next 5–8 years, and Table 1 shows the balance of seed and commercial production area to support the planned trajectory to achieve 10% of total rice production. The expansion and production targets were determined based on the goal of reaching 50% (approximately 1,000,000 households) of the VAD population in the Philippines. This estimate was derived from the figure that about 15.5% of the national population has VAD, as reported by DOST-FNRI. The production target was calculated using national per capita rice consumption data together with yield estimates to project the required production volume and potential coverage.

**Table 1:** Malusog Rice multi-year deployment targets and emerging outcomes.

Year	2023	2024	2025	2026	2027	2028	2029	2030
Malusog production (% of total)	0.0028	0.032	0.5	1%	3	5	7	10
Seed Production Area (ha)	23	200	312	624	1,871	3,119	4,366	6,238
Commercial Production Area (ha)	140	1,597	24,950	49,000	149,701	249,502	349,3030	499,004
No. of Households	1,305	100,000	160,000	300,000	500,000	600,000	800,000	1,000,000

### Survey & market testing results

The distribution of Malusog Rice to selected VAD households was conducted along with an acceptability survey. Results showed that 97% of the households expressed their desire to include Malusog Rice in their diet when it becomes widely available, 97% were willing to pay for Malusog Rice, and 95% were satisfied with the qualities of Malusog Rice in terms of storage, cooking, and eating qualities (Table 2).

**Table 2:** Number of households expressing willingness to adopt Malusog Rice and reporting satisfaction with its qualities in selected provinces during 2023.

ITEM	Overall *n* = 694	Surplus area				Deficit area		
		Quirino	Antique	Agusan del Sur	Maguindanao	Catanduanes	Samar	Lanao del Norte
		*n* = 96	*n* = 100	*n* = 100	*n* = 100	*n* = 98	*n* = 100	*n* = 100
Inclusion of MR in daily diet (%)								
*Will not include*	0.4	-	-	1.0	-	1.0	1.0	-
*Undecided*	2.2	4.2	5.0	2.0	-	2.0	-	2.0
*Will include*	97.4	95.8	95.0	97.0	100.0	96.9	99.0	98.0
Willingness to pay for MR (%)	97.0	94.8	95.0	97.0	99.0	96.9	99.0	97.0
Satisfaction with MR qualities (%)								
*Storage*	87.3	93.8	93.0	86.0	75.0	94.9	95.0	74.0
*Cooking qualities*	96.1	95.8	94.0	97.0	95.0	95.9	95.0	100.0
*Eating qualities*	95.8	96.9	94.0	96.0	95.0	96.9	96.0	96.0
*Overall qualities*	95.5	95.8	90.0	96.0	95.0	96.9	96.0	92.0

For the market testing, results indicate that Malusog Rice is a marketable product, with a price (0.74–0.96 USD/kg) comparable to regular/well-milled rice (0.68–1.08 USD/kg) (See Supplementary Table S1 for the comparison of prices between Malusog rice and regular/well-milled rice). Partner-retailers across the target provinces reported repurchase counts ranging from 5 to 11 (See Supplementary Table S1 for the number of repurchases), though limited supply (500 kg/retailer) and short selling periods in some areas may have constrained repeat purchases. On average, customers initially purchased 1 kg of Malusog Rice as a trial of the product (Table 3). All 14 participating partner retailers were willing to resell Malusog Rice and restock a volume of 77 bags per week (Table 3).

**Table 3:** Market testing of Malusog Rice conducted in 10 provinces during 2023, indicating that the product is marketable at a price comparable to regular or well‑milled rice (See Supplementary Table S1 for the selling prices of Malusog Rice, with price comparison with regular, well-milled, and premium rice).

ITEM	Overall	Pilot Provinces^1^							Other provinces^2^		
		Quirino	Antique	Agusan del Sur	Maguindanao	Catanduanes	Samar	Lanao del Norte	Nueva Ecija	Pampanga	Tarlac
Selling price of MR (USD/kg)^3^	0.84	0.82	0.88	0.96	0.90	0.90	0.84	0.90	0.76	0.74	0.74
Commonly purchased volumes (kg)^4^	1.0	1.0	1.0	25.0	1.0	1.0; 2.0	1.0	1.0	1.0; 3.0	1.0; 2.0; 5.0	1.0; 2.0
MR volume to be stocked (bag)	77.0	30.0	18.0	50.0	150.0	28.0	18.0	113.0	35.0	310.0	13.0

^1^ Conducted during the last quarter of 2023.

^2^ Conducted during the first quarter of 2023; highly rice commercial areas.

^3^ Each peso is worth $0.02 USD.

^4^ 1.0; 2.0; 5.0; 25 indicates that 1.0, 2.0,5.0, and 25.0 kg were commonly purchased quantities.

### GOVERNANCE: Creating an enabling environment for the smooth deployment and uptake of Malusog Rice

A science-driven policy advocacy initiative, along with stakeholder engagement, was implemented to gain support from local government units and policymakers toward the adoption of Malusog Rice. Activities conducted were dialogue and consultation, policy pitching, capacity building, and partnerships. These activities resulted in 23 resolutions issued in 23 provinces/municipalities, 9 institutional policies, 32 statements of support, 19 buyback arrangements mostly by the LGUs in the target provinces, 5 partner-initiated cookfests, 11 partner-initiated feeding programs, 42 partner-initiated publicity materials, 170 farmers participated in the cultivation of Malusog Rice, and 700 households participated in the acceptability study. Table 4 summarizes the programs where Malusog Rice has been used/integrated during the pilot-scale deployment period (See Supplementary Document 19 for documentation of stakeholder support for Malusog Rice, as reported in the Malusog Rice newsletter).

**Table 4:** Local nutrition programs of local government units and private organizations in the deployment sites integrating Malusog Rice, 2022–2023.

Region	Area	Program
I	Batac, Ilocos Norte	Feeding Program
I	Pangasinan (Second District)	Rice Relief Program (AyudaMark) by Congressman Mark Cojuangco
II	San Mateo, Isabela	Supplementary Feeding
II	Cabarroguis, Quirino	Healthy Diet Gawing Affordable (Nutrition Month Campaign)
IV-A	Cabuyao, Laguna	Donation Program of St. Ignatius Academy
IV-A	Infanta, Quezon	Feeding Program
V	Ligao City, Albay	Nutrition Care Package Program
V	San Miguel, Catanduanes	Food for Work
VIII	Calbiga, Samar	Kainan Supplementary Program
CARAGA	Dinagat Islands	Feeding Program

## Discussion

### SUPPLY: Field performance of Malusog Rice and ensuring its accessibility

The field performance of Malusog Rice recorded during WS 2022 to WS 2023 was comparable with the existing popular inbred rice varieties. Swamy^2,3^ reported that Malusog Rice is compositionally comparable to popular rice varieties in the Philippines, except for the provitamin A trait. Results of the biosafety assessment also concurred that Malusog Rice is the same as conventional rice, except for the added beta-carotene content. This means that it requires no changes in crop management practices and inputs. This evidence strengthens the case for promoting Malusog Rice as an alternative to existing rice varieties. The carotenoid levels of the harvest samples of Malusog Rice during WS 2022 to WS 2023 were within the desired level to meet 30–50% of the estimated vitamin A requirement of the target population. These results are consistent with the findings of Swamy^3^ who reported that Malusog Rice lines containing the GR2E event accumulated sufficient provitamin A carotenoids to contribute meaningfully to dietary vitamin A intake. Swamy^3^ further stressed that the varying levels of carotenoids in Malusog Rice grain are mainly due to genetic background of the rice variety, environmental growing conditions, and natural variability in plant metabolism.

The seed and commercial production of Malusog Rice during the pilot implementation was conducted in selected areas through techno demo and on-station schemes in partnership with PhilRice stations, regional DA experimental stations, and selected seed growers and farmers. To increase consumer access to newly released biofortified rice varieties, it is urgently necessary to improve seed chain accessibility^14^. It needs to be taken up by value chains and integrated into consumer diets^15^. Once the seed certification guidelines specifically developed for Malusog Rice are approved by the National Seed Industry Council (NSIC) and Malusog Rice has been cleared from any legal cases, the beta-carotene-enriched biofortified rice will be produced via the existing formal seed system, with seed certification administered by the Bureau of Plant Industry (BPI) - National Seed Quality Control Services (NSQCS) across the country. Moreover, DA-PhilRice and IRRI have continued their breeding work to develop additional nutritious rice through biofortification. According to Mugode^16^, biofortification with beta-carotene can deliver meaningful amounts of provitamin A, even though degradation of beta-carotene degradation was observed during storage and cooking under the Zambian practices. De Moura^17^ supported that biofortification remains effective despite the carotenoid degradation and recommended that it is a viable strategy to combat vitamin A deficiency.

### DEMAND CREATION: Social marketing, communication, consumer acceptance, and market testing

Our social marketing and communication activities conducted during the pilot deployment are consistent with broader work in promoting biofortified products. Birol & Bouis^18^ emphasized that demand creation through social marketing and education are necessary for adoption. They particularly stressed that providing information on the health benefits of biofortified products means higher acceptance rate and higher willingness to pay for the products. Oswalt^19^ supported this claim, noting that consumers are willing to pay a premium for zinc-biofortified rice, particularly when informed about its health benefits. Once promoted at scale, the messaging focusing on health benefits of Malusog Rice must be sustained. In addition, consistent with the observations of Birol and Bouis^18^, the color of biofortified products did not hinder consumers from purchasing and consuming the product.

The conduct of acceptability study and market testing for Malusog Rice are aligned with the findings of Talsma^20^ who claimed that marketability and consumer acceptability are crucial to the success of biofortified rice. The positive result of the acceptability survey of Malusog Rice also corroborates the findings of Birol & Bouis^18^ who reported that consumers are willing to pay as much – if not more – for food made with biofortified varieties of crops. This favorable consumer acceptance of Malusog Rice implies that there is a demand for Malusog Rice. Hence, farmers will be more likely to plant it. It also provides evidence of its viability to be integrated into government programs that promote nutrition. The encouraging market testing outcomes of Malusog Rice further demonstrate its potential for consumer adoption, as evidenced by the willingness of the retailers to restock. Marketing Malusog Rice is unlikely to encounter major barriers, as according to Custodio^21^, Filipinos valued healthier rice. Oswalt^19^ noted that awareness campaigns must be done to significantly increase consumer willingness to pay for biofortified rice.

### DEMAND CREATION: Providing access to target population

The pilot-scale deployment of Malusog Rice in the Philippines provided the first household access to the beta carotene-enriched rice in the world. When more supply becomes available and Malusog Rice is cleared from all legal, regulatory, and certification requirements, the plan is to expand to other areas with high prevalence of VAD and stunting in partnership with key stakeholders. Leveraging the lessons learned from the pilot-scale deployment, the nutrient-dense rice will be rolled out through a dual pathway: (1) program-based pathway; and (2) market-driven pathway for scale up and expansion:

#### Program-based pathway

 The program-based approach will be employed to help address VAD in close collaboration with the nutrition sector and ensure farmers have access to this nutritious variety through the government rice and seed distribution programs. It will tap existing programs and interventions by national government agencies such as the Department of Health, Department of Social Welfare and Development, and National Nutrition Council, among others, and non-governmental organizations (NGOs)—both local and international—to facilitate the promotion and acceptability of Malusog Rice. The goal is a full integration of Malusog Rice into the different government health and nutrition programs, such that it becomes the preferred variety in feeding programs driven by the LGUs and national program implementers. The inclusion of Malusog Rice into government seed distribution programs (e.g., National Rice Program) is also being targeted to ensure a steady supply of seeds in alignment with flagship programs of the Department of Agriculture.

#### Market-driven pathway

The market-based pathway will ensure that there is a sustainable supply and demand for Malusog Rice in the rice industry. A stable consumer demand for Malusog Rice is important to provide strong incentives for the rice value chain to sustain planting, processing, and distributing Malusog Rice to the target consumer segments. The positive response from the market tests conducted during pilot-scale deployment demonstrates that Malusog Rice is in the position to be scaled-up for wider reach. The program needs to ensure that an efficient and effective flow of Malusog Rice from farmers to target end-users is established. Extensive planning and marketing work will have to be carried out to include market segmentation. Value chain actors such as traders and retailers can play a crucial role in informing these plans about industry best practices. Priority consumer segments for Malusog Rice may come from VAD vulnerable and pilot area households and will not exclude other market segments that may be interested.

### GOVERNANCE: Policy advocacy and stakeholder outreach

The results demonstrate that policy advocacy coupled with extensive stakeholder outreach is needed to gain support for the adoption of Malusog Rice. De Steur^13^ strongly recommended this initiative, noting that the approval of Malusog Rice in the Philippines must be backed up with institutional commitment to ensure long-term success. Also consistent with their recommendation is that the deployment ensured that Malusog Rice was not promoted as a standalone project but as a part of a broader food-based approach. Bouis^22^ also saw this integration of biofortification into public and private policies, programs, and investments as a sustainable strategy to successfully deliver the technology to those who need it the most. Bouis^23^ stressed that biofortification is a cost-effective, nutrition-smart agricultural investment that is being pursued by national governments, several US agencies, and international financial organizations. They argued that while many building blocks are in place, institutional leadership is needed to continue to drive towards this ambitious goal. The role of policymakers is crucial in creating an enabling environment for smooth adoption of technology. It is therefore imperative that the advocacy and stakeholder outreach conducted for Malusog Rice must be sustained up to the point where it demonstrates a positive impact on the communities in need.

### Challenges and solutions applied

Some challenges were encountered during its initial deployment. Firstly, there was a limited supply of seeds and grains. To address this, key stakeholders, including the media, were actively engaged to present clear timelines. There was low awareness on Malusog Rice in the target areas^24^. Consequently, a coordinated public education campaign was launched involving local organizations, champions, and advocates. This includes preparation of learning modules on health and nutrition and biotechnology, conduct of community briefings, courtesy calls to local officials and leaders, and participation in partner-initiated events. Thirdly, anti-GMOs approached LGUs and implemented similar campaigns to stop the introduction of Malusog Rice as it is a genetically modified product. The program implemented the risk communication strategies and pursued policy advocacy activities with concerned LGUs. As discussed earlier, we were able to gain support from various stakeholders especially from LGUs.

### Strengths and limitations of the study

The pilot deployment of Malusog Rice provided valuable initial insights into farmer, retailer, and community experiences. This record of experience will serve as a practical reference for future rollouts within the Philippines and as a resource for other countries intending to develop and promote biofortified rice varieties. By documenting these early observations, the study contributes to the broader knowledge base on introducing biofortified crops at the community level.

A limitation of this study is the absence of exhaustive qualitative and quantitative research conducted by an independent external group. While the pilot deployment has yielded useful perspectives, these accounts can be substantiated in future work by conducting more comprehensive studies, including in-depth interviews, focus groups, and large-scale surveys, to provide a more robust evaluation of the initial deployment and its broader impact.

## Conclusion & Recommendations

The experience, success stories, and lessons learned from the pilot-scale deployment of Malusog Rice serve as “proof points” of the potential of the beta-carotene-enriched rice to complement existing interventions to help address VAD in the country. Our findings confirm that Malusog Rice performs reliably in the field, gains traction among consumers when supported by communication strategies, and is enabled by a favorable policy environment — all of which are necessary to ensure adoption of the technology. The results of the acceptability study and market testing showed that Malusog Rice is highly acceptable and marketable. This holds great potential in generating income for local farmers while providing the benefit of additional vitamin A to Filipino consumers.

It is recommended to (1) harness all these learnings from the past years of going through research and development, deregulation, and pilot-scale deployment, (2) continue the investment for at least another 5 years to be able to conduct implementation research to include stewardship, integration in the food value chain, and (3) measure the outcomes among farmers, stakeholders at the community, and households. This is crucial to ensure product integrity and traceability, and to manage critical points, until such time that the beta carotene-enriched rice becomes smoothly integrated into the Filipino food system. Future investments should also support the development of other healthier rice. The DA-PhilRice and IRRI breeding team is working on another nutrient-dense rice called high-iron and high-zinc rice (HIZR). HIZR currently undergoes the final stages of biosafety regulatory assessment. Once it is deregulated, the plan is to stack it with beta carotene-enriched Malusog Rice to develop and register 3-in-1 rice varieties. The ultimate goal is to champion food‑based solutions that break the cycle of malnutrition and build healthier futures. Lastly, the legal challenges and certification requirements have hindered the full commercialization of Malusog Rice. Plans for continuation and scale-up will proceed as soon as the technology overcomes these challenges.

## Supplementary Information

Below is the link to the electronic supplementary material.

Supplementary Material 1

Supplementary Material 2

## Data Availability

Data related to the development of Golden Rice have been published in peer-reviewed sources cited in the reference list, including: Potrykus (2010), New Biotechnology; Potrykus (2015), Annu Rev Plant Biol; Swamy et al. (2019), Journal of Agricultural and Food Chemistry; Oliva et al. (2020), Scientific Reports; Swamy et al. (2021), Scientific Reports; Wu et al. (2021), Proc Natl Acad Sci.Some datasets generated during the study are currently being organized and may be packaged for release in separate publications like books, monographs, and scientific papers. These will be made available at a later stage or upon reasonable request from the corresponding author. Additionally, certain datasets related to Malusog Rice are subject to restrictions due to an ongoing legal case. While they cannot be publicly disclosed at this time, they may be obtained through direct coordination with the corresponding author, subject to applicable conditions. Supporting data and protocols are provided in the supplementary information files, including biosafety permits, confined testing approval, varietal registration, and ethical approvals. In addition to the supplementary files, biosafety approvals and varietal registration details for Malusog Rice are available in the reference list: Health Canada Approval (Health Canada, 2018), and US-FDA Approval (US-FDA, 2018). Malusog Rice deployment implementation guidelines and protocols as well as informed consents and legislative resolutions are available from the corresponding author upon reasonable request.
