# Supplementary material for: Pilot deployment of beta carotene-enriched rice (Golden Rice) in the Philippines
Source: Sci Rep. 2026 May 4;16:20499. doi: 10.1038/s41598-026-48565-5 (PMC13328446; doi:10.1038/s41598-026-48565-5)
Supplement: Supplementary file 1 — Supplementary Material 1 [file 41598_2026_48565_MOESM1_ESM.pdf]

PILOT DEPLOYMENT OF BETA CAROTENE-ENRICHED RICE (GOLDEN RICE) IN THE PHILIPPINES

Ronan G. Zagado[1,4,*], Marissa V. Romero[1], Jesusa C. Beltran[1], Fidela P. Bongat[1], Reynante L. Ordonio[1], Joy Bartolome A. Duldulao[1], Anielyn Y. Alibuyog[1], Victoria C. Lapitan[1], Albert Christian S. Suñer[1], Gerardo F. Estoy, Jr.[1], Mary Ann U. Baradi[1], Sailila E. Abdula[1], Ommal H. Abdulkadil[1], Rhemilyn Z. Relado-Sevilla[1], Raul M. Boncodin[2], Ma. Aileen A. Garcia[2], Ellen E. Villate[3], and Russell F. Reinke[2]

[1]Department of Agriculture - Philippine Rice Research Institute (DA-PhilRice), Maligaya, Science City of Muñoz, 3119 Nueva Ecija, Philippines; [2]International Rice Research Institute (IRRI), Los Baños, 4030 Laguna, Philippines; [3]Biotechnology Coalition of the Philippines (BCP), 47 Kalayaan Ave, Diliman, Quezon City, 1101 Metro Manila, Philippines; [4]Current affiliation: Central Luzon State University, Science City of Muñoz, 3119 Nueva Ecija, Philippines.

*Corresponding Author: ronang.zagado@clsu2.edu.ph

Supplementary Table S1. Price Comparison between Malusog Rice and Conventional Rice (regular, well-milled, and premium rice) across 10 provinces

Province	Top Selling SKU of Retailer (USD/kg)			Selling Price of MR (USD/kg)	# of Repurchase of MR
	Regular milled	Well-milled	Premium Rice		
Quirino	0.88			0.82	11
Catanduanes		1.08		0.90	5
Antique		1.0		0.88	
Samar		1.08		0.84	6
Agusan del Sur		1.0		0.96	
Lanao del Norte			1.18	0.90	16
Maguindanao		1.08	1.18	0.90	
Nueva Ecija*	0.72	0.80		0.76	7
Pampanga*	0.68			0.74	9
Tarlac*		0.8		0.74	9

MR = Malusog Rice

SKU = Stock Keeping Unit

*conducted earlier than other provinces

Each peso is worth about $0.02 USD

USD = United States Dollar
