# Supplementary material for: Pilot deployment of beta carotene-enriched rice (Golden Rice) in the Philippines
Source: Sci Rep. 2026 May 4;16:20499. doi: 10.1038/s41598-026-48565-5 (PMC13328446; doi:10.1038/s41598-026-48565-5)
Supplement: Supplementary file 2 — Supplementary Material 2 [file 41598_2026_48565_MOESM2_ESM.pdf]

PILOT DEPLOYMENT OF BETA CAROTENE-ENRICHED RICE (GOLDEN RICE) IN THE PHILIPPINES

Ronan G. Zagado[1,4,*], Marissa V. Romero[1], Jesusa C. Beltran[1], Fidela P. Bongat[1], Reynante L. Ordonio[1], Joy Bartolome A. Duldulao[1], Anielyn Y. Alibuyog[1], Victoria C. Lapitan[1], Albert Christian S. Suñer[1], Gerardo F. Estoy, Jr.[1], Mary Ann U. Baradi[1], Sailila E. Abdula[1], Ommal H. Abdulkadil[1], Rhemilyn Z. Relado-Sevilla[1], Raul M. Boncodin[2], Ma. Aileen A. Garcia[2], Ellen E. Villate[3], and Russell F. Reinke[2]

[1]Department of Agriculture - Philippine Rice Research Institute (DA-PhilRice), Maligaya, Science City of Muñoz, 3119 Nueva Ecija, Philippines; [2]International Rice Research Institute (IRRI), Los Baños, 4030 Laguna, Philippines; [3]Biotechnology Coalition of the Philippines (BCP), 47 Kalayaan Ave, Diliman, Quezon City, 1101 Metro Manila, Philippines; [4]Current affiliation: Central Luzon State University, Science City of Muñoz, 3119 Nueva Ecija, Philippines.

*Corresponding Author: ronang.zagado@clsu2.edu.ph

Methodology for calculating how much Vitamin A is provided from Golden Rice

Milled Rice → Useful carotenoids → Cooking losses → Bioconversion rate → RAE

Milled Rice: We only measure total carotenoids after two months storage to account for losses

Useful carotenoids: Includes all-trans-β-carotene and other provitamin A carotenoids (β-cryptoxanthin, α-carotene, and other cis isomers of β-carotene) with 50% the conversion rate of all-trans-β-carotene

Cooking losses: We measured 15-23% losses, rounded to 30%

Bioconversion rate: Based on Tang et al. (2012), where conversion factor for children was 2.3:1 compared to adult conversion factor of 3.8:1 (reported in Tang et al. 2009)

RAE: Retinol activity equivalent - the vitamin A that can be used by the body

ONE DA

GOLDEN RICE
Malapit na 'to!

100 g approximates to one cup of Golden Rice, with 4.5ppm total carotenoids (TC)

We measured 15-23% losses, and rounded to 30%

For children 1-3 the Estimated Average Requirement (EAR) is 210 (ug/day RAE)

100 g milled Rice (4.5 µg/g)	Useful carotenoids (3.6 µg/g)	Cooking losses (2.52 µg/g)	Bioconversion rate (2.8:1)	42% of EAR
450 µg	360µg	252 µg	88µg	88/210=42%

All-trans-β-carotene (61% of TC), plus β-cryptoxanthin, α-carotene, and other cis isomers of β-carotene, converted at 50% the rate of all-trans-β-carotene (31%/2= 15.5%) = 80%

This is a conservative value greater than the 2.3 noted for children by Tang (2012)

ONE DA

GOLDEN RICE
Malapit na 'to!

Republic of the Philippines
Department of Agriculture
BUREAU OF PLANT INDUSTRY
692 San Andres St., Malate, Manila

Biosafety Permit for Field Trial
Number 19-001

Field trial proposal ***"Field Trial to Generate Data for Environmental Biosafety Risk Assessment of Beta-Carotene Enriched "Golden Rice" Event GR2E in the Philippines"*** of the Philippine Rice Research Institute (PHILRICE) with office address at PHILRICE Experimental Station, Maligaya, Science City of Munoz, 3119 Nueva Ecija has satisfactorily completed biosafety risk assessment for field trial pursuant to the DOST-DA-DENR-DOH-DILG Joint Department Circular No. 1 series of 2016.

The Bureau of Plant Industry, in consultation with the different concerned national agencies, has approved the conduct of the proposed activity in Brgy. Maligaya, Science City of Munoz, Nueva Ecija, with back-up site in Brgy. Malasin, San Mateo, Isabela, subject to the following conditions:

a. The proponent shall submit to the BPI the duly accomplished Oath of Undertaking before commencing with the field trial (The Oath of Understanding shall contain the objectives of the field test);
b. The proponent has the option to consult with the National Seed Industry Council (NSIC) regarding Rice National Cooperative Testing if there is an intention to register the variety;
c. The proponent shall submit to the BPI and inform the designated monitors of the detailed schedule of planting, planting plan, cultural management practices, monitoring activities and pest damage assessment;
d. The proponent shall adhere as closely as possible to the planting schedules for these sites. Modification in the planting schedule and other activities could be made only with the concurrence of BPI;
e. The proponent shall strictly observe the 100m physical isolation and 30-day temporal isolation at all times, from rice fields outside the enclosed experimental area; regular monitoring/inspection of the surrounding 100m radius isolation shall be done;
f. The proponent shall ensure that three (3) rows of corn plants will be grown around the site to further prevent pollen dispersal;
g. The proponent shall ensure the security of the experiment, and safety of the field workers and the monitors;
h. The proponent shall ensure that the workers and monitors have access to health facilities as the need arises;
i. The proponent shall be held accountable/responsible for any inadvertent release of the regulated plant material into the surrounding environment and shall undertake the necessary mitigating measures to address the incident;
j. The proponent shall ensure that only authorized persons are allowed inside the trial sites;
k. The proponent shall ensure that access to the site is limited. Visitors, staff and personnel are strictly monitored (physically search) during the maturity of the grain;
l. The proponent shall ensure that stray animals, birds and rodents are excluded from the trial sites while tests are being conducted;
m. The proponent shall ensure that all materials saved for other studies are well accounted for;
n. Immediately after harvest, the proponent shall ensure that all viable plant parts are heat killed (incineration is not allowed) and the emerged seedlings will be uprooted, chopped and buried at a designated spot in the site;

o. Immediately after harvest, the proponent shall ensure that the area shall be flooded after which volunteer plants will be monitored;

p. The proponent shall ensure that the area may be re-used for the second season planting only if no volunteer plants are observed after successive ten (10) day monitoring interval;

q. The permit holder shall notify the Director of BPI, within the time periods and in the manner specified below, in case of any of the following occurrences:

   r.1. Immediately upon discovery, not exceeding twenty-four (24) hours, through verifiable means of communication (email, text message, etc.), in the event that new information becomes available indicating that the regulated article could pose greater risks to biodiversity, human and animal health than its conventional counterpart; and

   r.2. In writing, as soon as possible, but not to exceed three (3) working days, if the regulated article or associated host organism is found to have characteristics substantially different from those listed in the application, or suffers from any unusual occurrence (e.g., excessive mortality or morbidity, unanticipated effect on non-target organisms);

r. The proponent shall strictly comply with the additional requirements that the BPI may impose, as necessary, during the trial period;

s. The proponent shall strictly adhere to the provisions of DA MC No. 02 Series of 2000 "Guidelines on the conduct of field test of plants derived from modem biotechnology"; and

t. The proponent shall comply with the reportorial requirements as discussed in Section 38 Item A and B of the DOST-DA-DENR-DOH-DILG Joint Department Circular No. 1 series of 2016.

This permit is valid for a period of two (2) years from the time of issuance subject to extension as maybe necessary to complete the field trial, unless sooner revoked for any of the reasons set forth in Article V Section 11 Item L of the DOST-DA-DENR-DOH-DILG Joint Department Circular No. 1 series of 2016.

Issued this **May** 20 **2019** at the Bureau of Plant Industry, San Andres St., Malate, Manila.

GEORGE Y. CULASTE
OIC-Director

I have read and understood the Terms and Conditions herein set forth and I enter in this agreement voluntarily and with my express consent, I here unto affix my signature with full knowledge of its legal effect.

REYNANTE L. ORDONIO
Signature over printed name of the Technology Developer

May 23, 2019
Date

Supplementary Document 3 (biosafety permit for FFP)

Republic of the Philippines
Department of Agriculture
BUREAU OF PLANT INDUSTRY
692 San Andres St., Malate, Manila

Biosafety Permit for Direct Use as Food and Feed, or for Processing

Number 19-060FFP

Rice GR2E owned and licensed by Philippine Rice Research Institute (PHILRICE) and International Rice Research Institute (IRRI), with office addresses at PHILRICE Central Experiment Station, Maligaya, Science City of Munoz, Nueva Ecija and Pili Drive, University of the Philippines Los Banos, Los Banos, Laguna, respectively, has undergone satisfactory biosafety assessment for the issuance of biosafety permit for direct use as food and feed, or for processing, pursuant to the DOST-DA-DENR-DOH-DILG Joint Department Circular No. 1, Series of 2016 and found to be as safe as conventional rice.

The permittee shall take appropriate measures to prevent accidental and unintentional release/reproduction and its recurrence and thereby protect human and animal health, and the environment. A Plant Quarantine Officer and his/her duly authorized representatives shall be allowed access during regular business hours to the facility where the regulated article is located and to any records relating to the importation of the regulated article, if applicable.

Issued on **December 10, 2019** at the Bureau of Plant Industry, San Andres St., Malate, Manila subject to conditions stated at the back of this permit, and shall expire on **December 9, 2024**.

GEORGE Y. CULASTE, PhD
Director
Bureau of Plant Industry

Biosafety Permit Conditions

1. The permittee shall duly inform the public of this approval by way of publishing in any one of the top three leading newspapers in the country. A copy of such publication shall be submitted to the Bureau of Plant Industry within ten days after publication;
2. If the regulated article is to be imported:
    a. There shall be strict monitoring of the regulated article from port of entry to the traders/importers storage/warehouse as stated in Section 32 of JDC 1 s2016;
    b. The importer shall ensure the proper and secure packaging of the regulated article for transport and the safety and durability of the transport vehicle, for prevention of any possible spillage or unintended release during transport/import. The Bureau of Plant Industry (BPI) shall conduct inspection of the shipment at the port of entry;
    c. In case of accidental release of rice GR2E, such as road spill during transport, it is the responsibility of the importers to inform the Bureau of Plant Industry (BPI) immediately. The BPI shall impose this condition on the Sanitary and Phytosanitary Import Clearance (SPSIC). PHILRICE and IRRI will assist the BPI to prevent unwanted germination of the GM seeds.
3. Control and mitigating measures as identified in the Environmental Management Plan (EMP) must be complied to prevent unwanted release of GM seeds into the environment;
4. If new information on the regulated article and its effects on human health and the environment becomes available, and such information is relevant and significant, the risk assessment shall be readdressed to determine whether the risk has changed or whether there is a need to amend the risk management strategies accordingly; and
5. The permit holder shall comply with the conditions set by the BPI as stated in the Biosafety Permit for Direct Use (Article 7, Section 21.H of the JDC 1, s.2016).

Violation of any condition shall lead to revocation of this permit as provided in the DOST-DA-DENR-DOH-DILG Joint Department Circular No.1, Series of 2016 Article VII, Section 21, J. In the event new information becomes available indicating that the regulated article could pose greater risks to biodiversity, human and animal health than its conventional counterpart, the applicant shall, on its own, immediately take measures necessary to protect human and animal health, and the environment.

I have read and understood the Terms and Conditions herein set forth and I enter in this agreement voluntarily and with my express consent, I here unto affix my signature with full knowledge of its legal effect.

JOHN C. DE LEON

Signature over printed name of the Technology Developer

Dec. 13, 2019

Date

Republic of the Philippines
Department of Agriculture
BUREAU OF PLANT INDUSTRY
692 San Andres St., Malate, Manila

Biosafety Permit for Commercial Propagation
Number 21-012Propa

GR2E Rice owned and licensed by Philippine Rice Research Institute., with office address at PhilRice-Central Experiment Station, Maligaya, Science City of Muñoz, Nueva Ecija, has undergone satisfactory biosafety assessment pursuant to the DOST-DA-DENR-DOH-DILG Joint Department Circular No. 1, Series of 2016. This permit is hereby issued for propagating the seeds of the said regulated article. Further, the permittee has satisfactorily complied with all requirements for the issuance of biosafety permit for commercial propagation.

Issued on **July 21, 2021** at the Bureau of Plant Industry, San Andres St., Malate, Manila subject to conditions stated at the back of this permit, and shall expire on **July 20, 2026.**

GEORGE Y. CULASTE, PhD.
Director
Bureau of Plant Industry

Biosafety Permit Conditions

1. The permittee shall duly inform the public of this approval by way of publishing in any one of the top three leading newspapers in the country. A copy of such publication shall be submitted to the Bureau of Plant Industry within ten days after publication;
2. Limitation of planting and/or commercial propagation within agricultural lands, those of which are classified as Alienable and Disposable (A&D lands);
3. The permittee shall notify the Director of BPI, within the time periods and in the manner specified below, in case of any of the following occurrences:
   a. Immediately upon discovery, not exceeding twenty-four (24) hours, through verifiable means of communication (email, text message, etc.), in the event that new information becomes available indicating that the regulated article could pose greater risks to biodiversity, human and animal health than its conventional counterpart; and
   b. In writing, as soon as possible, but not to exceed three (3) working days, if the regulated article or associated host organism is found to have characteristics substantially different from those listed in the application, or suffers from any unusual occurrence (excessive mortality or morbidity, unanticipated effect on non-target organisms).
4. In the event new information becomes available indicating that the regulated article could pose greater risks to biodiversity, human and animal health than its conventional counterpart, the applicant shall, on its own, immediately take measures necessary to protect human health and the environment; and
5. The permit holder shall not cause the commercial propagation in areas where the local government unit has a known policy or ordinance prohibiting the propagation or entry of regulated articles. For this purpose, it shall include in the labeling of products that these are not intended for propagation in prohibited areas.

Violation of any condition shall lead to revocation of this permit as provided in the DOST-DA-DENR-DOH-DILG Joint Department Circular No.1, Series of 2016 Article VII, Section 21, J. In the event new information becomes available indicating that the regulated article could pose greater risks to biodiversity, human and animal health than its conventional counterpart, the applicant shall, on its own, immediately take measures necessary to protect human and animal health, and the environment.

I have read and understood the Terms and Conditions herein set forth and I enter in this agreement voluntarily and with my express consent, I here unto affix my signature with full knowledge of its legal effect.

REYNANTE L. ORDONIO

Signature over printed name of the Technology Developer

July 22, 2021

Date

FOOD STANDARDS Australia New Zealand
Te Mana Kounga Kai – Ahitereiria me Aotearoa

20 December 2017
[35-17]

Approval report – Application A1138

Food derived from Provitamin A Rice Line GR2E

Food Standards Australia New Zealand (FSANZ) has assessed an application made by the International Rice Research Institute to seek approval for food derived from rice line GR2E, genetically modified to produce provitamin A carotenoids, especially beta-carotene, in the grain.

On 3 August 2017, FSANZ sought submissions on a draft variation and published an associated report. FSANZ received 33 submissions.

FSANZ approved the draft variation on 6 December 2017. The Australia and New Zealand Ministerial Forum on Food Regulation was notified of FSANZ's decision on 19 December 2017

This Report is provided pursuant to paragraph 33(1)(b) of the *Food Standards Australia New Zealand Act 1991* (the FSANZ Act).

Table of contents

EXECUTIVE SUMMARY 2

1 INTRODUCTION 3

1.1 THE APPLICANT 3
1.2 THE APPLICATION 3
1.3 THE CURRENT STANDARDS 4
1.4 REASONS FOR ACCEPTING APPLICATION 4
1.5 PROCEDURE FOR ASSESSMENT 5
1.6 DECISION 5

2 SUMMARY OF THE FINDINGS 5

2.1 SUMMARY OF ISSUES RAISED IN SUBMISSIONS 5
2.2 SAFETY AND NUTRITION RISK ASSESSMENT 8
2.3 RISK MANAGEMENT 9
2.3.1 Labelling 10
2.3.2 Detection methodology 11
2.3.3 Trade considerations 12
2.4 RISK COMMUNICATION 12
2.4.1 Consultation 12
2.5 FSANZ ACT ASSESSMENT REQUIREMENTS 13
2.5.1 Section 29 13
2.5.2. Subsection 18(1) 15

3 REFERENCES 16

ATTACHMENT A – APPROVED DRAFT VARIATION TO THE *AUSTRALIA NEW ZEALAND FOOD STANDARDS CODE* 17
ATTACHMENT B – EXPLANATORY STATEMENT 19

Supporting documents

The following documents[1] which informed the assessment of this Application are available on the FSANZ website:

SD1 Safety Assessment Report (at Approval)
SD2 Nutrition Risk Assessment Report (at Approval)

[1] http://www.foodstandards.gov.au/code/applications/Pages/A1138GMriceGR2E.aspx

Executive summary

Food Standards Australia New Zealand (FSANZ) received an Application from the International Rice Research Institute on 16 November 2016. The Applicant requested a variation to Schedule 26 in the *Australia New Zealand Food Standards Code* (the Code) to include food from a new genetically modified (GM) rice (*Oryza sativa*) line, GR2E. This rice line has been genetically modified to produce beta (β)-carotene (the predominant form of provitamin A) and other minor provitamin A carotenoids in the endosperm of the rice grain.

GR2E is a new food crop designed to mitigate vitamin A deficiency in developing countries. GR2E is not intended to be used in the Australian or New Zealand food supplies. Approving this crop will prevent trade disruption should GR2E be inadvertently present in imported shipments of milled rice.

The primary objective of FSANZ in developing or varying a food regulatory measure, as stated in section 18 of the *Food Standards Australia New Zealand Act 1991* (FSANZ Act), is the protection of public health and safety. Accordingly, the safety assessment is a central part of considering an application.

The Safety Assessment of GM rice line GR2E is provided in Supporting Document 1 and the Nutrition Risk Assessment is provided in Supporting Document 2. No potential public health and safety concerns have been identified. Based on the data provided in the present Application, and other available information, food derived from line GR2E is considered to be as safe for human consumption as food derived from conventional rice cultivars.

The FSANZ Board has approved the draft variation to Schedule 26 (including permission for food derived from provitamin A rice line GR2E) and Standard 1.5.2 (a consequential amendment).

1 Introduction

1.1 The Applicant

The International Rice Research Institute (IRRI) is an independent, non-profit, research and educational institute dedicated to: reducing poverty and hunger through rice science; improving the health and welfare of rice farmers and consumers; and protecting the rice-growing environment for future generations.

1.2 The Application

Application A1138 was submitted by IRRI on 16 November 2016. It seeks a variation to Schedule 26 in the *Australia New Zealand Food Standards Code* (the Code) to include food from a new genetically modified (GM) rice (*Oryza sativa*) line, GR2E. This rice line has been genetically modified to produce beta (β)-carotene (the predominant form of provitamin A) and, to a lesser extent, the two other forms of provitamin A (α-carotene and β-cryptoxanthin) in the endosperm of the rice grain. This trait has been achieved through expression of a phytoene synthase protein (PSY1) encoded by a gene (*Zmpsy1*) from *Zea mays* (corn) and a carotene desaturase protein (CRTI) encoded by a gene (*crtI*) from the bacterium *Pantoea ananatis*. These two proteins, normally absent in rice endosperm, supply the necessary intermediates to support a functional β-carotene biosynthetic pathway. The collective name 'Golden Rice' has been used to describe a number of versions of rice containing these two proteins (not necessarily from the same genes as used in GR2E).

GR2E also contains the bacterial *phosphomannose isomerase* (PMI*)* gene which is derived from *Escherichia coli* strain K-12. Expression of the PMI protein in cells allows growth on mannose as a carbon source. This was used as a selectable marker to assist with identification of transformed rice cells in the early stages of selection. The PMI protein has been previously assessed by FSANZ in four corn applications – A564 (FSANZ 2006), A580 (FSANZ 2008a), A1001 (FSANZ 2008b) and A1060 (FSANZ 2012).

It is the Applicant's intention that lines containing the GR2E event be cultivated for humanitarian purposes in developing countries including Bangladesh, Indonesia and the Philippines which are at high risk of vitamin A deficiency (VAD) and where 30–70% of energy intake is derived from rice. The Applicant notes that GR2E rice will not solve the issue of population-based VAD for these populations but can be part of an overarching strategy to reduce VAD. Countries wishing to adopt the Golden Rice technology are free to introduce the GR2E event into preferred varieties that suit the local environment and meet certain criteria outlined in a Humanitarian Use Licence Agreement, subject to local regulatory arrangements.

Rice containing the GR2E event is not intended for commercialisation in Australia or New Zealand i.e. either for growing or intentional sale in the food supply. The Applicant has however applied for food approval because it is possible the rice could inadvertently enter the food supply via exports from countries that may supply significant quantities of milled[2] rice to Australia or New Zealand.

[2] Milled or white rice is rice that has had the embryo and outer seed layers removed, and therefore consists only of the starchy storage tissue known as the endosperm.

1.3 The current Standards

Pre-market approval is necessary before a GM food may enter the Australian and New Zealand food supply.

Approval of such foods is contingent on completion of a comprehensive pre-market safety assessment. Standard 1.5.2 – sets out the permission and conditions for the sale and use of food produced using gene technology (a GM food). Foods that have been assessed and approved are listed in Schedule 26.

Section 1.5.2—4 of Standard 1.5.2 also contains specific labelling provisions for approved GM foods.

GM foods and ingredients (including food additives and processing aids from GM sources) must be identified on labels with the words 'genetically modified' if novel DNA or novel protein (as defined in Standard 1.5.2) is present in the food.

Foods listed in subsections S26—3(2) and (3) of Schedule 26 must also be labelled with the words 'genetically modified', as well as any other additional labelling required by the Schedule regardless of the presence of novel DNA or novel protein in the foods. Foods listed in subsections S26—3(2) and (3) are considered to have an altered characteristic, such as an altered composition or nutritional profile, when compared to the existing counterpart food that is not produced using gene technology.

The requirement to label food as 'genetically modified' does not apply to GM food that:

- has been highly refined (other than food that has been altered), where the effect of the refining process is to remove novel DNA or novel protein
- is a substance used as a processing aid or a food additive, where novel DNA or novel protein from the substance does not remain present in the final food
- is a flavouring substance present in the food in a concentration of no more than 1 g/kg (0.1%)
- is intended for immediate consumption and which is prepared and sold from food premises and vending machines, including restaurants, take away outlets, caterers, or self-catering institutions
- is unintentionally present in the food in an amount of no more than 10 g/kg (or 1%) of each ingredient.

If the GM food for sale is not required to bear a label, the labelling information referred to in section 1.5.2—4 must accompany the food or be displayed in connection with the display of the food (in accordance with subsections 1.2.1—9(2) and (3) of Standard 1.2.1).

1.4 Reasons for accepting Application

The Application was accepted for assessment because:

- it complied with the procedural requirements under subsection 22(2) of the FSANZ Act
- it related to a matter that warranted the variation of a food regulatory measure
- it was not so similar to a previous application for the variation of a food regulatory measure that it ought to be rejected.

1.5 Procedure for assessment

The Application was assessed under the General Procedure.

1.6 Decision

The draft variation as proposed following assessment was approved without change. The variation takes effect on the date of gazettal. The approved draft variation is at Attachment A. The related explanatory statement is at Attachment B. An explanatory statement is required to accompany an instrument if it is lodged on the Federal Register of Legislation.

2 Summary of the findings

2.1 Summary of issues raised in submissions

A total of 33 submissions were received of which 11 were very similarly worded. A campaign (entitled *Speak up NOW for Golden Rice*) urging positive comments on GR2E to both FSANZ and the Philippines biotechnology regulator (Bureau of Plant Industry), which was also seeking public comments on GR2E at the same time as FSANZ, was initiated by the Cornell Alliance for Science[3]; and a further nine submissions were most likely generated as a result of the campaign but did not follow exactly the same wording as the 11 similar submissions. As a result of the campaign, submissions to FSANZ were received from individuals and non-government organisations in Japan, India, the Netherlands, Vietnam, China, Kenya, the U.S.A., Brazil, Spain, and Argentina, as well as from Australia and New Zealand.

Of the 33 submissions, 23 directly supported Option 1 – the approval of GR2E in the Code. No submissions from jurisdictions unreservedly supported Option 1 due to questions over whether GM foods were captured as part of fortification policy and labelling issues. Six submissions directly opposed Option 1 and two submissions implied (i.e. did not overtly state) opposition.

All those overtly opposed to approval of GR2E raised issues that are outside the scope of FSANZ's regulatory area and included concern about an approval influencing regulatory processes in the Philippines where GR2E has not yet been approved; the efficacy of GR2E in lessening Vitamin A deficiency (VAD) in affected countries; the social effect (particularly on farmers) of GR2E in VAD-affected countries; and general issues of GM food safety not related to the GR2E application.

The main issues, within scope, raised in the public consultation are addressed in Table 1.

[3] http://allianceforscience.cornell.edu/topic/gmo

Table 1: Summary of issues

Issue	Raised by	FSANZ response
Several policy issues regarding nutritionally modified foods by means of genetic modification were raised, including that:<br>• policy concerning biofortification should be considered by the Food Regulation Standing Committee (FRSC)<br>• the *Fortification of Foods with Vitamins and Minerals* Policy Guideline does not expressly exclude fortification by genetic means<br>• as the first nutritionally modified GM food application, it sets an important precedent regarding policy consideration<br>• Codex Alimentarius is currently working on a definition for biofortification.	• NSW Food Authority (NSWFA)<br>• SA Health<br>• Victorian Government Departments of Health & Human Services and Economic Development, Jobs, Transport & Resources (Vic Govt)<br>• New Zealand Ministry for Primary Industries (NZ MPI)	Policy review and development is the role of FRSC and the Forum on Food Regulation (the Forum), and not FSANZ.<br><br>As noted in section 2.5.3, fortification through genetic means was explicitly deemed to be beyond the scope in developing the *Fortification of Foods with Vitamins and Minerals* Policy Guideline. FSANZ is not aware of any further consideration of, or change to, this position by FRSC or the Forum.<br><br>Noted.<br><br>The ongoing work by the Codex Committee on Nutrition and Foods for Special Dietary Uses in drafting a biofortification definition is noted. No definition is yet confirmed and cannot be used as clear guidance at this time.
A 'stop clock' on the application could be activated while policy matters are considered.	• SA Health<br>• NSWFA	FSANZ must process applications in accordance with the *FSANZ Act 1991.* Section 109 of that Act sets out when FSANZ may stop the clock on an application. It permits FSANZ to suspend consideration of an application if the Forum has notified FSANZ that the Forum is formulating a policy guideline and the application, in FSANZ's opinion, would be affected by that policy guideline, once formulated. No such notification has been received from the Forum that would affect FSANZ's consideration of this application.
GR2E rice could be imported and sold in Australia and New Zealand without consideration of the impact on, or need for, the intake of Vitamin A.	• Vic Govt	FSANZ's nutrition risk assessment did consider the impact on potential β-carotene (provitamin A) intakes in the population. This found an estimated increase in intake equivalent to the amount of β-carotene from approximately 1 teaspoon or less of carrot juice (see sections 2.2, 2.3 and SD2).

Issue	Raised by	FSANZ response
GR2E is the first GM food application FSANZ has assessed that intentionally alters nutritional content; it would set a precedent if nutritional changes are not labelled.	• SA Health	FSANZ has previously assessed and approved food from five lines genetically modified for the purpose of changing the nutritional profile:<br>– high lysine corn line LY038 (a line specifically targeted to the animal feed industry and not intended to enter the food supply)<br>– high oleic acid soybean line DP-305423-1<br>– herbicide-tolerant high oleic acid soybean line MON87705<br>– soybean line MON87769 producing stearidonic acid<br>– reduced acrylamide potential and reduced browning potato line E12 (containing reduced levels of asparagine, fructose and glucose)<br><br>In the last four cases additional labelling to describe the nature of nutritional changes was not mandated. Therefore there is a precedent for not having additional labelling where the GM food has an altered nutritional content.<br><br>In each of these four cases, FSANZ has considered the potential for consumer confusion – see next response.
Consumers should be informed of the nutritional change through additional labelling, for example in the nutrition information panel (NIP).	• SA Health<br>• NZ MPI	FSANZ noted in the Call for Submissions that mandating a statement that the food has been genetically modified to contain Vitamin A as β-carotene could imply the food contributes a nutritionally significant amount of this vitamin, when the actual amount may be negligible, and therefore be potentially misleading to consumers.<br><br>FSANZ considers that the same issue would apply if a declaration of Vitamin A was mandated in the NIP. Further, consumers may not associate the Vitamin A content in the NIP with the ‘genetically modified’ statement appearing in the ingredient list or in conjunction with the name of the food.<br><br>A declaration in the NIP for a mixed food may also prevent consumers from linking the Vitamin A content with the GR2E ingredient. Other ingredients may be viewed as the source, and in some cases these other ingredients may also contribute to the Vitamin A content of the food.
Imported processed food containing food derived from GR2E would not be labelled.	• Slow Food Australia	All foods imported into Australia or New Zealand are required by law to comply with the Code requirements. FSANZ is proposing to apply the same regulatory approach that applies to all approved GM foods in Australia and New Zealand. If GR2E is approved in the Code, food derived from this GM line containing novel DNA or novel protein and/or beta-carotene would be required to carry the ‘genetically modified’ labelling statement. A few exceptions, as set out in the Code, would apply (for example, food intended for immediate consumption or certain highly refined products).<br><br>Refer to section 2.3.1 for the labelling requirements.

Issue	Raised by	FSANZ response
GR2E could end up in animal feed and enter the human food chain with no consumer awareness.	• Slow Food Australia	The Applicant notes that rice by-products, such as bran and straw, are used in livestock feed. The Code specifically excludes consideration of animals fed with approved GM food (whether intentional or accidental) as the animals are not themselves genetically modified and therefore the food products they produce (e.g. meat, milk and eggs) are not GM foods and do not require labelling. This regulatory approach has been in place since 2000 and applies to all approved GM foods in Australia and New Zealand.<br><br>FSANZ is not aware of any international or overseas legislation that requires food products derived from animals fed GM food to be labelled.
There has not been an appropriate level of public debate and disclosure within Australia about the potential for genetically modified rice to come into the country.	• Two private submitters	There has been ongoing public debate about GM foods in Australia since the GM food standard was first developed nearly 20 years ago. FSANZ welcomes enquiries on GM foods via a variety of communication interfaces and seeks to provide science-based and impartial information to all stakeholders. In addition, there is an open and transparent public consultation process for all GM food applications considered by FSANZ.<br><br>FSANZ has approved food from over 70 GM lines covering eight species, including rice. The first approval for food from GM rice was made in 2008. The majority of GM foods enter the Australian food supply as imported products as very few GM crops are licensed by the OGTR for commercial growing in Australia. In this respect food from GR2E, in the event it were to become available in Australia, would be no different from these other GM foods.

2.2 Safety and nutrition risk assessment

In conducting an assessment of food derived from GR2E, several criteria have been addressed including: a characterisation of the transferred gene sequences, their origin, function and stability in the rice genome; the changes at the level of DNA and protein in the whole food; compositional analyses; an evaluation of intended and unintended changes; and a nutrition risk assessment in relation to β-carotene intake.

FSANZ's safety assessment, as reported in Supporting Document 1 (SD1) and which deals with the genetic modification *per se*, did not identify any potential public health and safety concerns. FSANZ concluded from its safety assessment, that based on the data provided in the Application and other available information, food derived from GR2E is considered to be as safe for human consumption as food derived from conventional rice cultivars. SD1 focusses on human food safety and therefore does not address any risks to the environment that may occur as the result of growing GM plants used in food production or any risks to animals that may consume feed derived from GM plants. Some minor changes in the SD1 released with the call for submissions have been made, related to correction of typographical errors.

Supporting Document 2 (SD2) reports on the nutrition risk assessment undertaken by FSANZ and includes a nutrition hazard assessment that considered potential adverse effects associated with β-carotene intake, and a dietary intake assessment for β-carotene that assumes all rice (including brown and milled rice, rice bran and rice bran oil[4] that are consumed as is or in processed foods and mixed dishes) consumed in Australia and New Zealand are replaced with GR2E products.

Provitamin A carotenoids present in GR2E, namely alpha (α)-carotene, β-carotene and β-cryptoxanthin, are precursors of vitamin A that are widely available in vegetables, fruits and cereals. However, high intake of β-carotene in foods or from supplements has not been associated with vitamin A toxicity. Carotenemia, a clinically benign condition involving yellow to orange skin pigmentation, can occur after intakes of large amounts of carotene-rich foods or high doses of β-carotene (≥ 30 mg/day) in supplement form.

Daily intake of up to 50 mg β-carotene in supplemental form for several years did not result in adverse health effects in healthy people or people with different forms of cancer, except those with or at risk of developing lung cancer.

A slight, but statistically significant, increased incidence of lung cancer and mortality rate was shown in heavy smokers taking 20 mg β-carotene supplements per day for 5 to 8 years. This risk was shown to decline within four to six years after discontinuing β-carotene supplementation.

The dietary intake assessment concluded that if all rice in the Australian and New Zealand markets was replaced with GR2E products this may result in a 2–13% (40-336 µg per day) increase in estimated intakes of β-carotene by Australian and New Zealand population groups. The increase in β-carotene intakes is equivalent to the amount of β-carotene from approximately 1 teaspoon or less of carrot juice.

Based on a comparison of the doses resulting in no adverse effects in human studies and the relatively small increase in total dietary intake of β-carotene due to consumption of GR2E rice products, FSANZ concluded that GR2E rice consumption will not pose a nutritional risk to the Australian and New Zealand population.

2.3 Risk management

FSANZ considered the safety of the GR2E genetic modification, and public health nutrition issues that may arise should food derived from GR2E be sold in Australia and New Zealand (section 2.1, SD1 and SD2).

FSANZ notes that rice containing the GR2E event is not intended for commercialisation in Australia or New Zealand (as noted under section 1.2). It is also noted that GR2E paddy or brown rice could not be imported into Australia or New Zealand without an environmental approval from the Office of the Gene Technology Regulator in Australia (OGTR) or the Environmental Protection Authority in New Zealand (EPA) (see section 2.3.3). However, since these current arrangements may change in the future, approval of the GR2E rice line in the Code allows any food derived from it to be sold in Australia and New Zealand. FSANZ's dietary intake assessment (SD2) therefore assumed the replacement of all rice in the Australian and New Zealand markets with GR2E rice and its products, should these be commercialised in Australia and New Zealand in the future.

---

[4] Noting that the inclusion of rice bran and rice bran oil in the modelling is highly conservative as it is unlikely β-carotene would be present in these GR2E products.

Based on the conclusions of the safety assessment on the GR2E genetic modification (SD1), and the conclusions of the nutrition risk assessment (SD2), FSANZ considered that permitting food derived from GR2E to be sold in Australia and New Zealand poses no risk to public health and safety.

2.3.1 Labelling

2.3.1.1 Requirement to be labelled as 'genetically modified'

In accordance with the existing labelling provisions in Standard 1.5.2, food derived from GR2E would be required to be labelled as 'genetically modified' if it: contains novel DNA or novel protein; or is listed in the existing subsections S26—3(2) and (3) of Schedule 26 as being subject to the condition that the labelling must comply with section 1.5.2— 4 of Standard 1.5.2 (such food has altered characteristics).

FSANZ has determined that whole rice and unrefined rice products derived from line GR2E will contain novel DNA and novel protein, as well as an altered nutritional profile (contains β-carotene), and as such would be required to carry the mandatory statement 'genetically modified' on the label of the package of food. This labelling requirement will apply to rice sold as a single ingredient food (e.g. a package of rice) and when the rice is used as an ingredient in another food (e.g. rice flour, rice milk).

FSANZ has approved a draft variation to amend the Code, which includes inserting a new subsection S26—3(2A). This new provision extends the requirement to comply with the labelling requirement imposed by section 1.5.2—4 (as mentioned above) to food derived from provitamin A rice line GR2E containing beta-carotene as a result of the genetic modification. Section S26—3 ensures foods with an altered characteristic are labelled with the mandatory 'genetically modified' statement irrespective of the presence of novel DNA or novel protein. For example in the case of GR2E, there may be products such as rice malt syrup where novel DNA and novel protein is absent but the product may have an altered nutritional profile (contains β-carotene) that would trigger the requirement for the mandatory statement.

Another product from rice is rice bran oil. In accordance with the existing labelling provisions in Standard 1.5.2, rice bran oil derived from GR2E is unlikely to require labelling because it is unlikely to contain novel DNA or novel protein, or have an altered nutritional profile (β-carotene is absent). The composition and characteristics of this highly refined product will therefore be the same as rice bran oil made from conventionally produced (non-GM) rice.

In summary, Table 2 below lists scenarios in which the mandatory statement will or will not apply, if food derived from GR2E was ever made commercially available in Australia or New Zealand or was unintentionally present in a food (e.g. an imported food) in an amount of more than 10 g per kilogram of each ingredient.

Table 2: Application of labelling requirements for GR2E food and ingredients

GR2E Food/Ingredient	Mandatory statement
Contains novel DNA or novel protein	✓
Contains β-carotene	✓
Novel DNA or protein absent but contains β-carotene	✓
Novel DNA or protein and β-carotene not present<br>i.e. the same as its conventional (non-GM) counterpart	×

2.3.1.2 Need for additional labelling requirements

Labelling of GM food is intended to address the objective set out in paragraph 18(1)(b) of the FSANZ Act—the provision of adequate information relating to food to enable consumers to make informed choices. For this reason, FSANZ considered whether additional labelling (i.e. in addition to the mandatory 'genetically modified' statement described above) is required to alert consumers to the nature of the altered characteristic when compared to non-GM rice. Rice from line GR2E will appear yellow because β-carotene is present. However, FSANZ has not proposed additional mandatory labelling for the following reasons:

- Rice containing the GR2E event is not intended at this time to be sold commercially in Australia or New Zealand (as noted under section 1.2).

- If sold in Australia or New Zealand, rice containing the GR2E event will be required to be labelled as 'genetically modified' (see above). Ingredients derived from GR2E, would also be required to have the 'genetically modified' labelling statement if they contain novel DNA, novel protein or β-carotene. Other labelling requirements will also apply to such foods, including the requirements contained in Standards 1.2.2 (Information requirements – food identification) and 1.2.4 (Information requirements – statement of ingredients).

- If rice containing the GR2E event is unintentionally present in a food sold in Australia or New Zealand (e.g. imported rice) in an amount of more than 10 g per kilogram of each ingredient (that is, more than 1%), that food will be required to be labelled as 'genetically modified' (see above).

- Suppliers are unlikely to be able to make voluntary nutrition content claims or health claims because the amount of Vitamin A (β-carotene as retinol equivalents[5]) in GR2E will be insufficient to meet claim conditions. To require a statement to the effect that the food has been genetically modified to contain Vitamin A as β-carotene could imply the food contributes a nutritionally significant amount of this vitamin, when the actual amount may be negligible, and therefore be potentially misleading.

2.3.2 Detection methodology

An Expert Advisory Group (EAG), involving laboratory personnel and representatives of the Australian and New Zealand jurisdictions was formed by the Food Regulation Standing Committee's Implementation Sub-Committee[6] to identify and evaluate appropriate methods of analysis associated with all applications to FSANZ, including those applications for food derived from gene technology (GM applications).

[5] For an explanation of retinol equivalents see https://www.nrv.gov.au/nutrients/vitamin-a
[6] Now known as the Implementation Subcommittee for Food Regulation

The EAG indicated that for GM applications, the full DNA sequence of the insert and adjacent genomic DNA are sufficient data to be provided for analytical purposes. Using this information, any DNA analytical laboratory would have the capability to develop a PCR-based detection method. This sequence information was supplied by the Applicant for A1138. Further to this, however, an event-specific quantitative method for detection of GR2E has been developed and has been referenced on the website of the European Commission Joint Research Centre[7]

2.3.3 Trade considerations

The Applicant has indicated there is no intention to apply for commercial cultivation of GR2E in Australia of New Zealand. If cultivation were sought, it would require independent assessment and approval by the OGTR or EPA. Providing permission for growing, and/or distributing GR2E rice overseas is the responsibility of local regulatory agencies.

Although GR2E rice is not likely to be grown or sold in Australia or New Zealand, it may be inadvertently present in imported consignments of milled rice.

In 2013 Australia imported 145,370 tonnes of milled rice (representing around 45% of the rice consumed, according to figures in Ricepedia[8]) with the main suppliers being Thailand (49%), India (19%) and Pakistan (13%) (FAOSTAT 2017). In the same year, New Zealand imported 42,381 tonnes of milled rice with the main suppliers being Australia (39%), Thailand (26%), and the U.S. (13%). While none of these countries is currently targeted for growing GR2E, some of them are in general regions where GR2E rice may be grown and may inadvertently enter export consignments. Without a permission in the Code, a consignment of milled rice with a very small amount of GR2E rice present could be rejected at the Australian or New Zealand border and thereby create trade disruption.

This Application therefore facilitates trade and ensures the ongoing supply of milled rice into Australia and New Zealand.

It should be noted that uncooked GR2E paddy or brown rice could not be imported into Australia or New Zealand without an environmental approval from the OGTR or EPA because the presence of the embryo means the rice could be germinated i.e. would be regarded as a viable genetically modified organism.

2.4 Risk communication

2.4.1 Consultation

Consultation is a key part of FSANZ's standards development process. The process by which FSANZ considers standards matters is open, accountable, consultative and transparent. Public submissions are called to obtain the views of interested parties on issues raised by the Application and the impacts of regulatory options.

Public submissions were invited on a draft variation which was released for public comment between 3 August and 14 September 2017. The call for submissions was notified via the Notification Circular, media release and through FSANZ's social media tools and the publication, Food Standards News. Subscribers and interested parties were also notified.

---

[7] JRC method for detection of GR2E - http://gmo-crl.jrc.ec.europa.eu/gmomethods/entry?db=gmometh&id=qt-eve-os-001&q=id%3aQT-eve-OS*
[8] Ricepedia: the online authority on rice http://ricepedia.org/australia

FSANZ acknowledges the time taken by individuals and organisations to make submissions on this Application. Every submission on this Application was considered by the FSANZ Board. All comments are valued and contribute to the rigour of the safety assessment.

Documents relating to Application A1138, including submissions received, are available on the FSANZ website[9].

2.5 FSANZ Act assessment requirements

2.5.1 Section 29

2.5.1.1 Consideration of costs and benefits

The Office of Best Practice Regulation (OBPR), in a letter to FSANZ dated 24 November 2010, granted a standing exemption from the need for the OBPR to assess if a Regulatory Impact Statement is required for the approval of genetically modified foods (ref 12065).

This standing exemption was provided as such changes are considered as minor, machinery and deregulatory in nature. The exemption relates to the introduction of a food to the food supply that has been determined to be safe.

Notwithstanding the above exemption, FSANZ conducted a cost benefit analysis. That analysis found the direct and indirect benefits arising from a food regulatory measure developed or varied as a result of the Application outweigh the costs to the community, government or industry that would arise from the development or variation of that measure.

A consideration of the cost/benefit of the regulatory options is not intended to be an exhaustive, quantitative financial analysis of the options as most of the impacts that are considered cannot be assigned a dollar value. Rather, the analysis seeks to highlight the qualitative impacts of criteria that are relevant to each option. These criteria are deliberately limited to those involving broad areas such as trade, consumer information and compliance.

The cost/benefit analysis is based on rice containing event GR2E being commercialised (see section 2.3.3) noting that, if it were, cultivation in Australia or New Zealand would require separate regulatory approval.

Option 1 was selected.

Option 1 – Approve the draft variation

Industry: Rice derived from GR2E and its products would be permitted under the Code and therefore any rice grain imports that contained the GR2E event would not be prevented from entering Australia and New Zealand

The segregation of grain derived from GR2E from conventional rice grain, as for any GM crop, will be driven by industry based on market preferences. Implicit in this will be a due regard to the cost of segregation.

There may be additional costs to the food industry as food ingredients derived from GR2E would require the ‘genetically modified’ labelling statement if they contain novel DNA, novel protein or β-carotene.

[9] http://www.foodstandards.gov.au/code/applications/Pages/A1138GMriceGR2E.aspx

Since the permission relates to rice derived from GR2E and noting current OGTR or EPA requirements, this could potentially include future imports of such milled rice grain, or food products made overseas containing this brown or milled rice, or domestic products containing imported milled rice.

Consumers: Rice derived from GR2E has been assessed as being as safe as food from conventional lines of rice.

For GR2E rice grain or products containing novel DNA, novel protein or β-carotene, labelling would allow consumers wishing to avoid these products to do so.

If GR2E rice is approved for growing in overseas countries, it could be used in the manufacture of products using this co-mingled rice grain. This means that there would be no cost involved in having to exclude GR2E grain from co-mingling and hence that there would be no consequential need to increase the prices of foods that are manufactured using co-mingled rice grain.

Since the permission relates to rice derived from GR2E and noting current OGTR or EPA requirements, consumers could potentially have access to future imports of such milled rice grain, food products containing this brown or milled rice made overseas, or domestically made products containing imported milled rice.

Government: Approval would avoid any conflict with WTO obligations. As mentioned above, food from GR2E has been assessed as being as safe as food from conventional lines of rice.

This option would be cost neutral in terms of compliance costs, as monitoring is required irrespective of whether or not a GM food is approved.

In the case of approved GM foods, monitoring is required to ensure compliance with the labelling requirements, and in the case of GM foods that have not been approved, monitoring is required to ensure they are not illegally entering the food supply.

Option 2 – Reject the draft variation

As food derived from GR2E has been found to be as safe as food from conventional counterparts, not preparing a draft variation would offer little relative benefit to consumers, government and industry.

The direct and indirect benefits that would arise from a food regulatory measure developed or varied as a result of the application outweigh the costs to the community, Government or industry that would arise from the development or variation of the food regulatory measure.

2.5.1.2 Other measures

There are no other measures (whether available to FSANZ or not) that would be more cost-effective than a food regulatory measure developed or varied as a result of the Application.

2.5.1.3 Any relevant New Zealand standards

Standard 1.5.2 and Schedule 26 also apply in New Zealand.

2.5.1.4 Any other relevant matters

The Applicant has submitted applications for regulatory approval of GR2E to a number of other countries, as listed in Table 3.

Table 3: List of countries to which applications for regulatory approval of GR2E have been submitted

Country	Agency	Type of approval sought	Status
USA	Food & Drug Administration (FDA)	food & feed	Under assessment
Canada	Health Canada	food	Under assessment
Philippines	Department of Agriculture, Bureau of Plant Industry	food, feed, processing	Under assessment

2.5.2. Subsection 18(1)

FSANZ has had regard to the three objectives in subsection 18(1) of the FSANZ Act during the assessment.

2.5.2.1 Protection of public health and safety

Rice derived from GR2E has been assessed based on the data requirements for GM foods provided in the FSANZ *Application Handbook*[10] which, in turn reflect internationally-accepted GM food safety assessment guidelines. No public health and safety concerns were identified in this assessment.

Based on the available evidence, including detailed studies provided by the Applicant, food derived from GR2E is considered to be as safe and wholesome as food derived from other commercial rice lines.

As a result of the nutrition assessment undertaken by FSANZ, which specifically considered the intake of β-carotene, FSANZ concluded that GR2E rice consumption will not pose a nutritional risk to the Australian and New Zealand population.

2.5.2.2 The provision of adequate information relating to food to enable consumers to make informed choices

Food derived from GR2E would have to comply with labelling requirements as discussed in section 2.3.1 of this report. This will enable consumers to make informed choices in relation to such food.

2.5.2.3 The prevention of misleading or deceptive conduct

The provision of an event-specific detection method by the Applicant will permit the detection of food derived from GR2E (see section 2.3.2).

2.5.3 Subsection 18(2) considerations

FSANZ has also had regard to:

[10] http://www.foodstandards.gov.au/code/changes/pages/applicationshandbook.aspx

- **the need for standards to be based on risk analysis using the best available scientific evidence**

FSANZ's approach to the safety assessment of all GM foods applies concepts and principles outlined in the Codex Principles for the Risk Analysis of Foods derived from Biotechnology (Codex 2004). Based on these principles, the risk analysis undertaken for GR2E used the best scientific evidence available. The Applicant submitted to FSANZ a comprehensive dossier of quality-assured raw experimental data. In addition to the information supplied by the Applicant, other available resource material including published scientific literature and general technical information was used in the safety assessment.

- **the promotion of consistency between domestic and international food standards**

This was not a consideration as there are currently no relevant international standards. As noted in Table 1, Codex Alimentarius is undertaking work to develop a definition for biofortification. However, a definition is yet to be confirmed, and therefore the draft definition cannot be considered at this time.

- **the desirability of an efficient and internationally competitive food industry**

The inclusion of GM foods in the food supply, providing there are no safety concerns, allows for innovation by developers and a widening of the technological base for producing foods. GR2E is a new food crop designed to mitigate vitamin A deficiency in developing countries. While GR2E is not intended for the Australian or New Zealand food supplies, the approved draft variation will prevent trade disruption should there be inadvertent presence in imported shipments of milled rice.

- **the promotion of fair trading in food**

Issues related to consumer information and safety are considered in sections 2.2 and 2.3 above.

- **any written policy guidelines formulated by the Forum on Food Regulation**

No such policy guidelines apply to this Application. FSANZ notes that, in developing the Policy Guideline *Fortification of Food with Vitamins and Minerals*, 'fortification through genetic means' was explicitly deemed to be beyond the scope of the Guideline[11]. This is the same approach that equally applies to food containing a modified nutrient content achieved through conventional breeding techniques.

Policy review or development in regard to fortification of foods by genetic means is the role of FRSC and the Forum and not FSANZ (as discussed in Table 1).

3 References

Attachments

A. Approved draft variation to the *Australia New Zealand Food Standards Code*
B. Explanatory Statement

[11] http://foodregulation.gov.au/internet/fr/publishing.nsf/Content/24E85FF75806731ACA257FE300077FA7/$File/12-2003-FRSC-Consultation%20Paper-Fortification%20Food%20supply%20with%20Vitamins%20Minerals.docx

Attachment A – Approved draft variation to the *Australia New Zealand Food Standards Code*

Food Standards (Application A1138 – Food derived from Provitamin A Rice Line GR2E) Variation

---

The Board of Food Standards Australia New Zealand gives notice of the making of this variation under section 92 of the *Food Standards Australia New Zealand Act 1991*. The variation commences on the date specified in clause 3 of the variation.

Dated [To be completed by Standards Management Officer]

Standards Management Officer
Delegate of the Board of Food Standards Australia New Zealand

**Note:**<br><br>This variation will be published in the Commonwealth of Australia Gazette No. FSC XX on XX Month 20XX. This means that this date is the gazettal date for the purposes of the above notice.

1 Name

This instrument is the *Food Standards (Application A1138 – Food derived from Provitamin A Rice Line GR2E) Variation*.

2 Variation to Standards in the *Australia New Zealand Food Standards Code*

The Schedule varies standards in the *Australia New Zealand Food Standards Code*.

3 Commencement

The variation commences on the date of gazettal.

Schedule

[1] **Standard 1.5.2** is varied by omitting the words ‘subsections S26—3(2) and (3)’ from subparagraph 1.5.2—4(1)(a)(ii), substituting ‘section S26—3’.

[2] **Schedule 26** is varied by

[2.1] inserting after the Note to subsection S26—3(2)

(2A) Products containing beta-carotene from item 6(b) are subject to the condition that their labelling must comply with section 1.5.2—4

[2.2] inserting in the table to subsection S26—3(4), in alphabetical order under item 6

(b) provitamin A rice line GR2E

Attachment B – Explanatory Statement

1. Authority

Section 13 of the *Food Standards Australia New Zealand Act 1991* (the FSANZ Act) provides that the functions of Food Standards Australia New Zealand (the Authority) include the development of standards and variations of standards for inclusion in the *Australia New Zealand Food Standards Code* (the Code).

Division 1 of Part 3 of the FSANZ Act specifies that the Authority may accept applications for the development or variation of food regulatory measures, including standards. This Division also stipulates the procedure for considering an application for the development or variation of food regulatory measures.

The Authority accepted Application A1138 which seeks permission for the sale and use of food derived from a genetically modified rice line, GR2E, which produces provitamin A in the grain. The Authority considered the Application in accordance with Division 1 of Part 3 and has prepared a draft variation.

Following consideration by the Australia and New Zealand Ministerial Forum on Food Regulation, section 92 of the FSANZ Act stipulates that the Authority must publish a notice about the draft variation.

Section 94 of the FSANZ Act specifies that a variation of a standard in relation to which a notice is published under section 92 is a legislative instrument, but is not subject to parliamentary disallowance or sunsetting under the *Legislation Act 2003*.

2. Purpose

The purpose of this instrument is to amend Schedule 26 of the Code to permit the sale, or use in food, of food derived from provitamin A rice line GR2E and make a consequential amendment to Standard 1.5.2.

3. Documents incorporated by reference

The variations to food regulatory measures do not incorporate any documents by reference.

4. Consultation

In accordance with the procedure in Division 1 of Part 3 of the FSANZ Act, the Authority's consideration of Application A1138 included one round of public consultation following an assessment and the preparation of a draft variation and associated report. Submissions were called for on 3 August 2017 for a six-week consultation period.

The Office of Best Practice Regulation (OBPR), in a letter to FSANZ dated 24 November 2010, granted a standing exemption from the need for the OBPR to assess if a Regulatory Impact Statement is required for the approval of genetically modified foods (ref 12065). Therefore, a Regulation Impact Statement was not required in this case because the proposed amendments to Standard 1.5.2 and Schedule 26 are likely to have a minor impact on business and individuals.

5. Statement of compatibility with human rights

This instrument is exempt from the requirements for a statement of compatibility with human rights as it is a non-disallowable instrument under section 94 of the FSANZ Act.

6. Variation

Item [1] amends Standard 1.5.2 by replacing the reference to ‘subsections S26—3(2) and (3)’ in subparagraph 1.5.2—4(1)(a)(ii) with a reference to ‘section S26—3.’ This is a consequential amendment required as a result of the variation proposed by item [2.1] below.

Item [2] amends Schedule 26.

Subitem [2.1] inserts new subsection S26—3(2A) into Schedule 26. The new subsection requires food products containing beta-carotene derived from provitamin A rice line GR2E to comply with the labelling requirement imposed by section 1.5.2—4 of the Code.

Subitem [2.2] inserts new paragraph (b) into item 6 in the table to subsection S26—3(4). Paragraph (b) refers to ‘provitamin A rice line GR2E’. This amendment will permit the sale, or use in food, of food derived from provitamin A rice line GR2E.

U.S. FOOD & DRUG ADMINISTRATION

Biotechnology Notification File No. 000158
Note to the File

Date: May 8, 2018

From: Carrie McMahon, Ph.D., Consumer Safety Officer, DBGNR/OFAS/CFSAN

To: BNF 000158 Administrative Record

Subject: GR2E rice with altered composition (provitamin A carotenoids (mainly beta (β)-carotene))

Keywords:
Rice; *Oryza sativa* (L.); Gr2E rice; Golden Rice; provitamin A carotenoids; beta-carotene; β-carotene; *Zmpsy1* gene from *Zea mays* (corn); phytoene synthase; *Zm*PSY1; *crtI* gene from *Pantoea ananatis*; carotene desaturase I; CRTI; *pmi* gene from *Escherichia coli*; phosphomannose isomerase; PMI; selectable marker for plant transformation; New Protein Consultation No. (NPC) 000002; OECD unique identifier IR-ØØGR2E-5; International Rice Research Institute; IRRI.

Purpose

This document summarizes the Food and Drug Administration's (FDA's, our) evaluation of biotechnology notification file (BNF) No. 000158. International Rice Research Institute (IRRI) submitted a safety and nutritional assessment of genetically engineered (GE) rice, transformation event GR2E, which we received on November 14, 2016. In its submission, IRRI informed FDA that although GR2E rice is not currently intended for cultivation or marketing in the United States,[1] it anticipates that GR2E rice, or human and animal food products derived from GR2E rice, may enter the U.S. food supply via imports from countries of production. We evaluated the information in IRRI's submission to ensure that regulatory and safety issues regarding human or animal food derived from GR2E rice have been resolved.

In our evaluation, we considered all information provided by IRRI as well as publicly available information and information in the agency's files. Here we discuss the outcome of the consultation, but do not intend to restate the information provided in the final consultation in its entirety.

Intended Effect

The intended effect of the modification in GR2E rice is the production of provitamin A carotenoids (mainly β-carotene) in the rice endosperm. To confer this trait, IRRI introduced two genes that encode components of the carotenoid biosynthetic pathway: the *Zmpsy1* gene from *Zea mays* encodes phytoene synthase (PSY1) and the *crtI* gene from *Pantoea ananatis* encodes carotene desaturase I (CRTI). These enzymes catalyze the conversion of geranylgeranyl diphosphate to phytoene and the

[1] According to IRRI, GR2E rice is intended for cultivation and use in human food as a source of dietary provitamin A carotenoids (mainly β-carotene) in certain south and southeast Asian countries where vitamin A deficiency is common.

conversion of phytoene to lycopene, respectively. Lycopene is the precursor to provitamin A carotenoids, including β-carotene.

IRRI also introduced the *pmi* gene from *Escherichia coli*. This gene encodes phosphomannose isomerase (PMI) and was used by IRRI as a selectable marker for plant transformation.[2]

Regulatory Considerations

The purposes of this evaluation are (1) to assess whether the developer has introduced into human or animal food a substance requiring premarket approval as a food additive and (2) to determine whether use of the new plant variety in human or animal food raises other regulatory issues under the Federal Food, Drug and Cosmetic Act (FD&C Act).

Genetic Modification and Characterization

Transformation Plasmid and Method

IRRI transformed embryogenic calli of the japonica rice cultivar Kaybonnet with plasmid pSYN12424 using Agrobacterium-mediated transformation. The T-DNA region of pSYN12424 contains three expression cassettes between right and left border sequences:

- Cassette I: *crtI*[3] gene from *P. ananatis*, preceded by the N-terminal RUBISCO SSU transit peptide coding sequence from *Pisum sativum*, with regulatory elements, including the glutelin *GluA-2* promoter from *Oryza sativa* and nopaline synthase (NOS) 3′-untranslated region (3′-UTR) from *A. tumefaciens* Ti plasmid (pTiT37)
- Cassette II: *Zmpsy1* gene from *Z. mays* with regulatory elements, including the *GluA-2* promoter from *O. sativa* and NOS 3′-UTR from *A. tumefaciens* pTiT37
- Cassette III: *pmi* gene from *E. coli* K-12 with regulatory elements, including the polyubiquitin *ZmUBI* promoter region and first intron from *Z. mays* and NOS 3′-UTR from *A. tumefaciens* pTiT37

Following transformation, IRRI selected for transformants using mannose selection media to inhibit the growth of untransformed cells lacking expression the selectable marker, PMI. The proliferating transformed colonies were transferred to plant regeneration media. IRRI selected a transformed plant (T0) containing the GR2E event for propagation. The GR2E event was subsequently crossed into indica rice cultivars PSB Rc82, BRRI dhan 29, and IR64 for molecular characterization and compositional analyses.

Characteristics, Inheritance, and Stability of the Introduced DNA

IRRI characterized the insertion event in GR2E rice using Southern blot analyses and direct sequencing of PCR products amplified from the insertion site. IRRI concluded that GR2E rice contains a single, intact DNA insertion that is identical to the T-DNA region of plasmid pSYN12424, with the exception of small deletions at the ends of both the right and left borders. The two deletions do not affect the integrity of the three expression cassettes. IRRI used five probes corresponding to the entire plasmid backbone region to demonstrate by Southern blot analysis that GR2E rice does not contain plasmid backbone sequences, including antibiotic resistance marker genes.

IRRI used a combination of methods to demonstrate the inheritance as a single locus according to Mendelian principles and the stability of the inserted DNA, as well as the stability of the β-carotene

[2] PMI is the subject of New Protein Consultation No. (NPC) 000002. FDA responded to NPC 000002 on February 10, 2009.
[3] The *crtI* gene sequence in pSYN12424 contains an A3992 to G change compared to GenBank accession no. D90087 sequence. This nucleotide difference does not alter the amino acid sequence of the protein.

phenotype. IRRI confirmed the inheritance and stability of the inserted DNA using Southern blot analyses to analyze genomic DNA samples from four different germplasm backgrounds carrying the GR2E event (Kaybonnet, PSB Rc82, BRRI dhan 29, and IR64) and from multiple breeding generations.

IRRI also analyzed nucleotide sequences of the T-DNA insert and flanking rice genomic sequences to determine whether the integration event created new start-to-stop open reading frames (ORFs) that could potentially encode sequences homologous to known toxins or allergens. The analysis identified two potential new ORFs. IRRI then compared the amino acid sequences of the potential ORFs to the sequences of known and putative toxins[4] and allergens,[5] and reported that it did not identify significant sequence similarities. Based on these results, IRRI concluded that there were no new novel ORFs created as a consequence of the DNA insertion that would have the potential to encode a protein with any significant amino acid sequence similarity to known and putative toxins or allergens.

Protein Characterization

Identity and Function

GR2E rice was genetically engineered to express PSY1 from *Z. mays* and CRTI from *P. ananatis*. In immature rice grains, the carotenoid biosynthetic pathway functions up to the synthesis of geranylgeranyl diphosphate (GGPP). The PSY1 and CRTI enzymes bridge the gap between GGPP and lycopene, which is a precursor for α- and β-carotenoids. PSY1 catalyzes the condensation of two molecules of GGPP to form 15-*cis*-phytoene. CRTI then catalyzes the *cis* to *trans* conversion of 15-*cis*-phytoene to all-*trans*-lycopene. Lycopene is subsequently converted to α- and β-carotene by endogenous rice cyclases.

GR2E rice was also genetically engineered to express PMI from *E. coli*. PMI catalyzes the interconversion of mannose-6-phosphate and fructose-6-phosphate. PMI is used as a selectable marker by genetic engineers because plants expressing PMI are able to utilize mannose as their only or primary carbon source and thus grow on media lacking other carbon sources.

Protein Expression Levels

IRRI measured the levels of PSY1, CRTI and PMI in tissue samples from plants grown in the Philippines at four sites in 2015 (rainy season) and in 2016 (dry season). Composite samples were collected from at least five representative plants of each entry (GR2E introgressed into PSB Rc82 background (PSB Rc82+GRE2 rice) and from the near-isogenic, non-GE PSB Rc82 (the control)) within each of three replicated blocks. Grain samples were collected at the milk, dough, and mature stages of development; straw samples were collected at harvest. IRRI analyzed the samples using quantitative enzyme-linked immunosorbent assay and reported the results of its analyses. PSY1, CRTI, and PMI protein levels were below the limit of quantification (LOQ) in all tissue samples collected from the control rice. In tissue samples from the PSB Rc82+GR2E rice:

- Under the endosperm specific rice *GluA*-2 promoter, the PSY1 protein was present in grain but not straw samples. The highest levels in grain were measured in dough-stage samples and ranged from 308 – 359 ng/g fresh weight (FW) across both growing seasons. The highest level of PSY1 measured in mature rice grain samples was 245 ng/g FW.

---

[4] IRRI evaluated potential similarities to known and putative toxins using FASTA bioinformatic alignment (*E*-score of 1 x 10^{-5} or less) against a toxin database created from a subset of sequences from the UniProt Knowledgebase.
[5] IRRI evaluated potential similarities to known and putative allergens using FASTA bioinformatic alignment (>35% match over an 80 amino acid sliding window) against the Food Allergy Research and Resource Program (FARRP) allergen protein database. IRRI also screened for any 8 contiguous, identical amino acid matches to allergens in the FARRP database.

- Under the endosperm specific rice *GluA*-2 promoter, the CRTI protein was present in grain but not straw samples. The highest levels in grain were measured in dough-stage samples and ranged from 54 – 68 ng/g FW across both growing seasons. The highest level of CRTI measured in mature rice grain samples was 30 ng/g FW.
- Under the constitutive control of the *Z. mays* polyubiquitin promoter, the PMI protein was present in both grain and straw samples. The highest levels in grain were measured in dough-stage samples, which averaged about 2015 ng/g FW across the four locations over both growing seasons. PMI levels were on average about 1282 ng/g FW in mature rice grain across both growing seasons and ranged from 320 – 796 ng/g FW in straw depending on location and growing season.

Potential for Toxicity and Allergenicity

IRRI used a "weight of the evidence" approach to assess the potential toxicity and allergenicity of PSY1, CRTI, and PMI. In its safety assessment, IRRI discussed information about the history of dietary exposure to *Z. mays* PSY1 and to *E. coli* PMI protein homologs present in common food sources, as well as the likely incidental dietary exposure to *P. ananatis* CRTI. IRRI noted the lack of evidence of toxicity and allergenicity associated with these genetic sources. IRRI summarized unpublished acute oral toxicity studies of CRTI and PMI that demonstrated a lack of any test substance-related adverse effects. IRRI discussed information about the enzymatic functions of the three proteins and explained that these functions are not similar to the activities of known protein toxins. IRRI provided the results of standard bioinformatics analyses, which showed no significant amino acid sequence similarities to known and putative toxins[4] or allergens.[5,6] IRRI concluded that the PSY1, CRTI, and PMI proteins are unlikely to be toxic or allergenic to humans or animals.

IRRI discussed digestibility and heat stability test results for PSY1, CRTI, and PMI.[7] Digestibility studies using standard methods did not identify significant resistance to pepsin proteolysis. Heat stability studies using standard methods showed rapid and irreversible enzymatic inactivation of the proteins at temperatures below those normally used during processing or cooking of rice. IRRI concluded from these results that dietary exposure to intact and functional PSY1, CRTI, and PMI proteins will be negligible.

Based on the weight of this combined evidence, IRRI concluded that the PSY1, CRTI, and PMI proteins are unlikely to be toxic or allergenic to humans or animals through oral exposure.

Human and Animal Food Use

Oryza sativa L., commonly known as rice, is the dominant species of rice cultivated around the world for a variety of human and animal food uses. There are three types of *O. sativa* L.: japonica, indica, and javanica. After threshing and winnowing to remove chaff and other material, rice grain is known as paddy rice. Beginning with dehulling, paddy rice is further processed into a variety of food products for human consumption. Commonly consumed rice products are brown rice, milled rice, and parboiled rice. Processed rice is also consumed in prepared products such as rice noodles, cakes, crackers, sweets, and alcoholic beverages.

---

[6] IRRI identified one 8-amino acid identity match between PMI and a known allergen: α-parvalbumin from *Rana* species CH2001. However, IRRI concluded the match is not biologically meaningful because no cross-reactivity was observed between PMI and the serum of an individual with a documented IgE-mediated allergy to *Rana* species CH2001 α-parvalbumin.

[7] Because the concentrations of PSY1 and CRTI in GR2E rice are low, IRRI conducted the digestibility and heat stability studies using microbially-expressed PSY1 and CRTI. Prior to conducting the studies, IRRI confirmed the equivalence of the plant- and microbe-expressed proteins using mass spectral analysis of trypsin-digested protein samples as well as immunochemical cross-reactivity. For assessment of digestibility and heat stability of PMI, IRRI relied on published reports.

The by-products of food processing, as well as low grade paddy rice and whole crop silage, are used in animal food to varying degrees in different regions of the world. In the United States, rice grain, bran, hulls and polishings (material that is removed during the polishing process) are used in a wide range of livestock diets while rice may be used in pet foods.

Composition

Scope of Analyses

IRRI analyzed the composition of paddy rice, milled rice (hull removed), and straw, as well as bran samples, collected from event GR2E introgressed into PSB Rc82 background (PSB Rc82+GRE2 rice) and from the near-isogenic, non-GE PSB Rc82 (the control).

Study Design - Compositional Analyses

IRRI grew PSB Rc82+GR2E rice and the control over two growing seasons in 2015 (rainy) and 2016 (dry) in the Philippines at four sites, which IRRI states represent typical rice growing conditions. At each site, the rice varieties were planted using a randomized complete block design with three replicates, within which each entry was planted in a 10m^2 plot with 250 plants. Grain (paddy rice) and straw were collected from mature plants and pooled into a single sample per plot.[8] Composite samples of grain were processed to obtain bran samples.

IRRI analyzed the composition of paddy rice, straw, and bran.

- In paddy rice, IRRI reported the results for moisture, crude protein, crude fat, ash, fiber (acid detergent fiber, neutral detergent fiber, total dietary fiber, and crude fiber), carbohydrates by calculation, starch, amylose, 11 fatty acids, 18 amino acids, 9 minerals, 7 vitamins (including β-carotene), and antinutrients phytic acid and trypsin inhibitor.
- In straw and bran, IRRI reported the results for moisture, crude protein, crude fat, ash, ADF, NDF, crude fiber, carbohydrates by calculation, calcium, and phosphorus.

IRRI explained that compositional data were analyzed using mixed linear model for multi-year combined-sites analysis. The parameters included in the model were entry, site, and growing season and the appropriate interaction terms. For each component, the least squares (LS)-mean value across years and sites was estimated for PSB Rc82+GR2E rice and the control. IRRI used the LS-means to test for least significant differences between the components of PSB Rc82+GR2E rice and the control rice. Statistically significant differences were established at p-value < 0.05.

When a statistically significant difference was identified in the multi-year combined-sites analysis, IRRI evaluated the biological significance of this difference by comparing the individual PSB Rc82+GR2E rice component mean value with the range of values available in ILSI Crop Composition Database (2014) or that are reported in published literature.[9] IRRI concluded that ranges observed for PSB Rc82+GR2E rice that fell within the combined literature range for a given component are within the ranges of normal variability for conventional rice.

Results of analyses

Paddy rice, straw, and bran

IRRI analyzed 69 components in paddy rice and 10 components each in straw and bran. While no statistically significant differences were observed in either straw or bran, statistically significant

[8] Grain was collected from at least 150 plants per plot; straw was collected from at least 8 plants per plot.
[9] Published literature included OECD (2016), NARO (2011), Heuzé and Tran (2015), and Juliano and Bechtel (1985).

differences were observed in paddy rice for stearic acid and for β-carotene (the intended compositional change). IRRI concluded that, with the exception of β-carotene, the components measured in samples of PSB Rc82+GR2E rice, including stearic acid, were within or similar to the range of values observed for conventional rice varieties with a history of safe consumption.

Intended Compositional Change – provitamin A carotenoids (mainly β-carotene)

IRRI reported the results of analysis of β-carotene levels[10] in paddy rice samples from PSB Rc82+GR2E rice and the control. Whereas the LS-mean value for β-carotene in the control is below the LOQ (range = below LOQ to 0.07 mg/kg dry weight (DW)), the LS-mean value for β-carotene in PSB Rc82+GR2E rice is 1.26 mg/kg DW (range = 0.504 to 2.35 mg/kg DW).

IRRI also summarized unpublished results of a compositional analysis for β-carotene and other provitamin A carotenoids[11] in milled rice prepared from grain samples. As expected, the mean values of β-carotene and other carotenoids in the control rice were below the LOQ while the mean values in PSB Rc82+GR2E rice were quantifiable, with the highest mean reported for β-carotene (3.57 µg/g DW) and a total carotenoid mean of 5.88 µg/g DW[12]. IRRI attributes the differences in mean β-carotene levels (grain versus milled rice) to differences in the sample matrix and in the carotenoid extraction efficiencies of the two analytical methods employed.

In considering the safety of increased levels of β-carotene in human food, IRRI discussed the current dietary sources of β-carotene and other provitamin A carotenoids; dietary intakes by consumers in the United States from different sources, and the potential dietary exposure from consumption of GR2E rice. Assuming 100 percent of rice consumed in the United States is replaced with GR2E rice, IRRI used current dietary rice intake (11.8 kg per capita annually) and the highest value of β-carotene measured in samples of milled GR2E rice (7.31 µg/g DW) to estimate the potential increase in dietary exposure to β-carotene. IRRI estimated the potential dietary exposure to β-carotene from GR2E rice to be approximately 0.24 mg/day; this translates to approximately one tenth the current daily β-carotene consumption in the adult population, from all other food sources. IRRI acknowledged that it expects the actual dietary intakes to be lower given (1) that it is unlikely that all rice in the diet would be substituted with GR2E rice and (2) that β-carotene levels in food containing GR2E rice would decline over time due to storage, processing, and cooking.

IRRI discussed its consideration of the safety of β-carotene in animal diets. IRRI notes that with the exception of elevated levels of provitamin A carotenoids, there are no biologically meaningful differences in the composition of GR2E rice and conventional rice. IRRI highlights that β-carotene from synthetic or microbial sources are used as an additive in animal nutrition as a source of provitamin A. IRRI concludes that animal food derived from GR2E rice is as safe and nutritious as animal food derived from conventional rice varieties.

Endogenous Allergens

IRRI summarized what is known about rice allergies in humans, noting that while allergic reactions have been documented, rice is not considered to be a commonly allergenic food.

---

[10] Samples were analyzed using AOAC International Official Method 941.15 and data were reported as all-*trans*-β-carotene. Values obtained using this AOAC method may be influenced by the presence of other carotenoids. IRRI subsequently analyzed milled rice using a method that further differentiates carotenoids.

[11] Samples were analyzed using high performance liquid chromatography and concentrations of carotenoids were reported for β-cryptoxanthin, all-*trans*-α-carotene, β-carotene (as all-*trans*-β-carotene), 9′-*cis*-β-carotene, and total carotenoids.

[12] IRRI also reported mean β-cryptoxanthin (0.312 µg/g DW), all-*trans*-α-carotene (0.713 µg/g DW), and 9′-*cis*-β-carotene (07.62 µg/g DW).

Summary of Compositional Analyses

IRRI reports that, with the exception of provitamin A carotenoids, no consistent patterns emerged to suggest that biologically meaningful changes in composition or nutritive value of the grain or straw had occurred as an unintended consequence of the genetic modifications. Accordingly, IRRI concludes that human and animal food derived from GR2E rice is as safe and nutritious as food derived from conventional rice varieties.

Food Labeling Considerations

IRRI states that GR2E rice is not currently intended for use in human or animal food in the United States. However, in response to a request from FDA, IRRI explained, on October 9, 2017, its view regarding an appropriate common and usual name for rice derived from event GR2E in the ingredient statement of food products containing this rice if such products were to enter the US food supply.

Although GR2E rice is not intended for distribution in the United States, it is a producer's or distributer's responsibility to ensure that labeling of the foods it markets meets applicable legal requirements. Although the levels of β-carotene in GR2E rice are too low to warrant a nutrient content claim, the β-carotene in the endosperm of GR2E rice results in grain that is yellow-golden in color. We note that the name "Golden Rice" has been used by IRRI and others for many years to identify β-carotene-expressing rice varieties under development. CFSAN's Office of Nutrition and Food Labeling, Food Labeling and Standards Staff (ONFL/FLSS) considers that this name would accurately describe GR2E rice if present in human food. If companies market GR2E rice in human food in the United States, we advise them to consult with ONFL/FLSS to discuss any required or voluntary labeling including statements relating to attributes of this rice or any other type of claim.

FDA's Center for Veterinary Medicine, Office of Surveillance and Compliance, Division of Animal Feeds (OSC/DAF) has determined that "rice" is the appropriate name for GR2E rice if present in animal food. OSC/DAF notes that although GR2E rice is altered in color, color is not an important attribute of animal food unless it would be expected to impact food coloration despite the other ingredients that could be present in the food.

On July 19, 2016, the National Bioengineered Food Disclosure Law (Public Law 114-216) charged the United States Department of Agriculture's Agriculture Marketing Service with developing a national mandatory system for disclosing the presence of bioengineered material in food. Producers, distributers, and marketers of GR2E rice are responsible for following requirements issued by USDA relevant to the labeling of their products.

Conclusion

FDA evaluated IRRI's submission to determine whether GR2E rice raises any safety or regulatory issues with respect to its uses in human or animal food. Based on the information provided by the company and other information available to the agency, FDA did not identify any safety or regulatory issues under the FD&C Act that would require further evaluation at this time.

IRRI has concluded that its rice variety, GR2E rice, and the human and animal foods derived from it are as safe as and, with the exception of β-carotene expression, are not materially different in composition or any other relevant parameter from other rice varieties now grown, marketed, and consumed. At this time, based on IRRI's data and information, the agency considers IRRI's consultation on GR2E rice to be complete.

Carrie H. Mcmahon -S
Digitally signed by Carrie H. Mcmahon -S
Date: 2018.05.08 13:53:54 -04'00'
____
Carrie McMahon, Ph.D.

Novel Food Information – Provitamin A Biofortified Rice Event GR2E (Golden Rice)

canada.ca/en/health-canada/services/food-nutrition/genetically-modified-foods-other-novel-foods/approved-products/novel-food-information-golden-rice-gr2e.html

Health Canada has notified the International Rice Research Institute that it has no objection to the food use of Provitamin A Biofortified Rice Event GR2E (Golden Rice). The Department conducted a comprehensive assessment of this rice event according to its Guidelines for the Safety Assessment of Novel Foods. These Guidelines are based upon internationally accepted principles for establishing the safety of foods with novel traits.

Background:

The following provides a summary of the notification from the International Rice Research Institute and the evaluation by Heath Canada and contains no confidential business information.

1. Introduction

The International Rice Research Institute (IRRI) has developed a genetically modified rice event (GR2E) using recombinant-DNA techniques that is biofortified with provitamin A. GR2E rice will be grown commercially in major rice-producing regions, primarily in Asia. The main intended market for this product is in countries such as Bangladesh and the Philippines where diets are typically low in vitamin A. Intrinsic food supplementation could be a useful tool for alleviating vitamin A deficiency (VAD) in children, a known and preventable cause of blindness. The IRRI has indicated that this product is not intended to be sold in Canada.

The safety assessment performed by Food Directorate evaluators was conducted according to Health Canada's Guidelines for the Safety Assessment of Novel Foods. These Guidelines are based on harmonization efforts with other regulatory authorities and reflect international guidance documents in this area (e.g., Codex Alimentarius). The assessment considered: how this rice event was developed; how the composition and nutritional quality of event GR2E compared to non-modified rice; and the potential for the GR2E rice event to be toxic or cause allergic reactions. The IRRI has provided data that demonstrate that Provitamin A Biofortified Rice Event GR2E is as safe as traditional rice varieties used as food in Canada.

The Food Directorate has a legislated responsibility for pre-market assessment of novel foods and novel food ingredients as detailed in the Food and Drug Regulations (Division 28). Provitamin A Biofortified Rice Event GR2E is considered a novel food under the following part of the definition of novel foods: "c) a food that is derived from a plant, animal or microorganism that has been genetically modified such that

1. the plant, animal or microorganism exhibits characteristics that were not previously observed in that plant, animal or microorganism."

2. Development of the Modified Plant

The petitioner provided information describing the methods used to develop GR2E rice and molecular biology data that characterized the genetic change that results in the accumulation of provitamin A carotenoids. GR2E rice was produced using Agrobacterium tumefaciens (A. tumefaciens) mediated transformation of the japonica rice cultivar Kaybonnet with the transformation vector pSYN12424. This transformation vector was constructed to contain three gene cassettes, one for the crtI gene, one for the Zmpsy1 gene and one for the phosphomannose isomerase (pmi) selectable marker gene.

The crtI gene in the first expression unit derives from the bacteria Pantoea ananatis and is fused in frame at the 5' end with the RUBISCO SSU of Pisum sativum. P. ananatis is a Gram-negative species and is found widely in the environment, both in water and soil and as part of the flora associated with plant and animal hosts. While P. ananatis is an uncommon opportunistic human pathogen, the derived DNA encoding for CRTI lacks similarity to potential pathogenicity determinants. The RUBISCO SSU encodes a transit peptide in order to localize the protein to the chloroplast. The expressed phytoene desaturase enzyme, CRTI, catalyzes the conversion of 15-cis-phytoene to all-trans-lycopene.

The psy1 gene in the second expression unit derives from Zea mays (maize). The expressed enzyme, ZmPSY1, is a phytoene synthase which converts geranylgeranyl diphosphate into phytoene, and acts upstream of CRTI in the carotenoid biosynthesis pathway.

Together the expression of these proteins results in the production of lycopene in rice, which is used by naturally occurring mechanisms in the plant to produce provitamin A carotenoids. The expression of both of these gene cassettes is driven by the rice glutelin promoter (GluA-2), which targets expression to the rice endosperm.

The gene expressed in the third cassette, pmi, encodes a phosphomannose isomerase enzyme derived from Escherichia coli. This enzyme catalyzes the reversible isomerization of mannose-6-phosphate to fructose-6-phosphate. This serves as a selection marker for the ability of transformed calli and plantlets to grow on medium that contains mannose. The expression of pmi is controlled by the maize ubiquitin (ZmUbi1) promoter, associated intron, and 5'-UTR resulting in constitutive expression. The PMI protein has previously been assessed by Health Canada in several authorized maize events.

The original transformant T0, was self-crossed to create the T1 generation, which was then crossed into three Indica rice cultivars, PSB Rc82, BRRI dhan 29, and IR64. Each of these crosses was then backcrossed and self-crossed multiple times until they reached the BC5F4,

BC5F3 and BC5F3 generations respectively. The data presented in the molecular characterisation of GR2E rice is from generations throughout the breeding trees of each of the T1 x Indica rice variety crosses.

3. Characterization of the Modified Plant

The number of insert sites for the pSYN12424 plasmid T-DNA and the integrity of the genetic elements were investigated using Southern blot analyses using three probes and a combination of restriction enzymes. The Southern blot data supports the assertion that there is a single intact copy of the T-DNA insert contained in the GR2E rice, containing three expression cassettes for Zmpsy1, crtI, and pmi expression. No partial insertions or rearrangements of the insert were detected. The results were the same between the original genetic background, as well as three hybrid crosses with other lines of Indica rice, which suggests that inheritance of the entire cassette is stable in a breeding program.

Genomic samples from the controls and GR2E rice in the Kaybonnet background were also tested by Southern blot analysis using a set of five probes, which together covered the entire pSYN12424 plasmid backbone. No hybridizing fragments were detected in the GR2E rice samples demonstrating that no part of the plasmid backbone was inserted into the GR2E rice genome.

In order to assess the overall integrity of the insert and all its component elements, as well as to detect any single base-pair changes that may have occurred, the petitioner sequenced the entire insert in GR2E rice. Based on the known sequence of the insert and preliminary sequence information for the 5' and 3' flanking regions, seven PCR primer sets were designed to amplify the entire region of interest in overlapping fragments. Two deletions were found in the border regions, 23 bp at the right border and 11 bp at the left border. This type of deletion is common in Agrobacterium-mediated transformation. Aside from the border deletions, the remaining sequence was intact and identical to the T-DNA region of pSYN12424.

The sequenced genomic DNA was subject to ORF analysis in order to assess the possibility that the insertion could have created new unintended proteins that could be homologous to toxins or allergens. The deduced amino acid sequences of the identified ORFs were used to query toxin and allergen databases. No significant hits were returned for either database and it was concluded that the potential spurious ORFs are unlikely to be potential toxins or allergens.

The stability of the inserted DNA was assessed for four generations of GR2E rice using Southern blots. The consistent and expected banding patterns in the Southern blots observed across breeding generations supports the conclusion that the T-DNA is stably integrated and inherited in a typical breeding program.

Trait stability was also investigated by phenotypic analysis, specifically elevated production of β-carotene in the rice grain of the same four generations used in the Southern blots. The presented data demonstrated carotenoid expression was correlated with the presence of the T-DNA, although there was variation in expression levels. However, the observation that carotenoid accumulated in GR2E rice seed contained in different germplasm backgrounds and across several generations supports the conclusion that the carotenoid expression trait is stably inherited.

The petitioner also conducted a study of the inheritance of the inserted T-DNA using a PCR-based method to detect the presence or absence of the insert in progeny from three segregating generations. Statistical analysis of this data demonstrated a segregation pattern consistent with the expected Mendelian pattern for a single insertion site.

Enzyme-linked immunosorbent assays (ELISAs) were used to approximately measure the amount of protein in the different tissues, namely grain and straw (stems) from plants grown in four locations during either the rainy season (2015) or the dry season (2016). The range and mean protein levels were reported for each tissue type as nanograms per gram fresh tissue weight, uncorrected for extraction efficiency. The highest levels were measured in the dough stage grain, with levels ranging from 308-359 ng/g and 54-68 ng/g for ZmPSY1 and CRTI, respectively, across both growing seasons. In mature grain, the highest protein levels detected were 245 ng/g for ZmPSY1 and 30 ng/g for CRTI. The concentration of PMI protein was higher than for the other two expressed proteins, with a mean concentration in mature rice grain of 1282 ng/g across both seasons. PMI was also detected in straw tissue at a mean concentration of 482 ng/g.

Expression of ZmPSY1 and CRTI proteins in GR2E rice is low and thus could not be purified in sufficient quantity for use in subsequent testing. Therefore, these proteins were expressed in a bacterial (i.e., Escherichia coli) expression system and were characterised using SDS-PAGE, reverse phase HPLC, amino acid analysis, MALDI MS/MS peptide mapping, N-terminal amino acid sequencing, and enzymatic activity. Taken together, the data presented by the petitioner supports the equivalence between the E. coli expressed reference proteins and their plant produced counterparts.

The amino acid sequence identity the of PMI protein expressed in GR2E rice is identical to that of PMI expressed in maize events MIR162, 3272 and 5307. Based on this information no additional characterization was required to demonstrate the equivalence to the proteins used in previously submitted studies.

4. Product Information

Provitamin A Biofortified Rice Event GR2E differs from traditional counterparts through the presence of provitamin A in milled rice. All photosynthetic tissues of higher plants produce and accumulate β-carotene. Rice is usually consumed in milled form, which does not contain provitamin A carotenoids. Milling removes the carotenoid-containing embryo and aleurone

layer from rice grains to leave only the endosperm. Germplasm screening has not succeeded in identifying any rice cultivars that accumulate provitamin A in the endosperm, and so conventional breeding was not a viable avenue for introducing the desired trait.

Immature rice endosperm produces a precursor for carotenoid biosynthesis, geranylgeranyl diphosphate (GGDP). The incorporation of the genes that produce the enzymes, phytoene synthase (PSY) and multifunctional CRTI, permits GR2E rice to convert GGDP to lycopene. Bridging this gap in the carotenoid biosynthetic pathway allows the endogenous lycopene cyclase enzymes expressed in the rice endosperm to convert lycopene into a mixture of α- and β-carotene, the provitamin A compounds. Rice event GR2E can accumulate up to 30 μg/g total carotenoids in the endosperm, of which about 80% are mixed isomers of β-carotene.

5. Dietary Exposure

GR2E is intended for cultivation and use in a number of South and Southeast Asian countries. However, it may be possible that raw commodity or food products derived from GR2E rice may unintentionally enter Canada via imports from countries of production. The introduction of GR2E rice in the target markets is not expected to alter the use or consumption patterns of rice in Canada.

For the purposes of estimating daily dietary exposure, the petitioner used historic data from the highest rice-consuming countries in Asia. Based on this data, the upper limit of mean daily dietary intake of rice was set at 12.5 g/kg body weight, which accounts for consumption by all subpopulations, including children who are the highest consumers.

Although the petitioner indicated that Canada is not the intended market for this product, Health Canada analyzed baseline intake data for rice and estimated β-carotene intake if all the rice consumed by Canadians was replaced with GR2E rice. Replacement of all rice and rice products in Canada with GR2E rice would result in a very small 0.8-8% (34 μg-239 μg per day) increase in β-carotene intake. It should be noted that this estimate is conservative as it is unlikely that all rice consumed would be GR2E rice containing β-carotene at the highest level measured.

6. Nutrition

The data provided by the petitioner on the compositional studies conducted on GR2E rice were reviewed by Health Canada to determine whether the use of GR2E rice is safe relative to its conventional counterparts. The efficacy of the GR2E rice in helping vitamin A deficiency in affected populations was not evaluated.

Compositional data for GR2E rice and its near-isogenic, non-transgenic conventional counterpart were collected from four field trials over 2 years in the rice growing regions of the Philippines. In each study three blocks (replicates) of each entry (event GR2E and

control) were planted at each test site in a randomized complete block design. The petitioner states that at each site, planting and cultivation were done according to local agronomic practices. Normal pest control and maintenance practices consistent for the locale were used to produce the rice..

The grain samples were collected from mature rice plants, which represented the state where typical grain harvest occurs. The nutritional components measured in grain, straw, and bran were chosen based on recommendations of the OECD for comparative assessment of the composition of new varieties of rice (2016). The analyses for each component were conducted on all samples by a single laboratory using internationally approved and validated analytical methods and following consistent and appropriate sample storage and preparation procedures.

Specifically, test and control samples were analyzed for nutrients, anti-nutrients, and secondary metabolites, in paddy rice, as follows: Proximate(s) and fiber: moisture, crude protein, crude fat, ash, carbohydrates; fiber (crude fiber, acid detergent fiber (ADF), neutral detergent fiber (NDF), total dietary fiber (TDF)); Sugars: amylose Minerals: calcium, copper, iron, magnesium, manganese, phosphorus, potassium, sodium, zinc; Vitamins: B1 (thiamine), B2 (riboflavin), B3 (Niacin), B6 (pyridoxine), B9 (folic acid), E (α-tocopherol); Fatty Acid(s): Caprylic (C8:0), Capric (C10:0), Lauric (C12:0), Myristic (C14:0), Pentadecanoic (C15:0), Palmitic (16:0), Palmitoleic (C16:1), Heptadecanoic (C17:0), Stearic (18:0), Oleic (C18:1), Linoleic (C18:2), Alpha-linolenic (C18:3), Arachidic (20:0), Eicosenoic (C20:1), Eicosadienoic (C20:2), Eicosatrienoic (C20:3), Arachidonic (C20:4), Behenic (C22:0), Erucic (C22:1), Lignoceric (C24:0), Nervonic (C24:1); Amino Acid(s): Alanine, Arginine, Aspartic acid, Cysteine, Glutamic acid, Glycine, Histidine, Isoleucine, Leucine, Lysine, Methionine, Phenylalanine, Proline, Serine, Threonine, Trytophan, Tyrosine, Valine; Anti-nutrients: Phytic acid, and Trypsin inhibitor.

The data collected from the multi-year combined site studies were analyzed using statistically appropriate methodology. Where a statistically significant difference ($P < 0.05$) was identified in the multi-year combined site analysis, further context for interpreting the possible biological significance of the difference was gathered through comparisons with the range of values for each analyte reported in the published literature or available from the ILSI Crop Composition Database. Analyte ranges for GR2E rice that fell within the combined literature range for that analyte were considered to be within the range of normal variability of conventional rice.

In addition to the compositional components listed above, milled rice samples of GR2E and control were also analysed for all-trans-β-carotene and other carotenoids (β-cryptoxanthin, all-trans-α-carotene, 9′-cis-β-carotene, and total carotenoids). The concentrations of all-trans-β-carotene ranged from 1.96–7.31 μg/g dry matter across locations and years and on average comprised of approximately 59% of the total carotenoids as determined by HPLC. This was followed by all-trans-α-carotene (12%) and β-cryptoxanthin (5%).

With the exception of provitamin A carotenoids, which were intended to be elevated in the GR2E rice, there were no significant differences observed between the non-genetically modified conventional counterpart and the GR2E rice for any of the proximates, minerals, amino acids, vitamins, fatty acids (except for stearic acid), and secondary metabolites. The mean value for stearic acid was within the range reported values found in literature and the ILSI database.

The absorption of β-carotene from foods ranges from ~2% to 65%. Several factors affect the bioavailability of carotenoids including the food matrix, extent of cooking, and amount of co-ingested fat, e.g. in a typical mixed North American diet, β-carotene absorption ranges from 12-16%. There is negative feedback regulation of β-carotene intestinal absorption and of conversion due to increasing cellular levels of all-trans-retinoic acid. The vitamin A equivalency for β-carotene from foods ranges from 3.8:1 (Golden Rice) to 28:1(leafy green vegetables) but even for pure β-carotene in oil, extremes of 55:1 or more have been reported based on vitamin A status and genetic polymorphisms, e.g., in the genes coding for enzymes involved in the conversion of β-carotene to vitamin A (Haskell 2012). The Institute of Medicine currently estimates a vitamin A equivalency ratio for plant sources of β-carotene is 12:1 by weight (i.e. 12 μg β-carotene is equal to 1 μg retinol), whereas for β-cryproxanthin and α-carotene is 24:1. The provitamin A activity of 9-cis and 13-cis isomers of β-carotene is less than 10% of all-trans-β-carotene.

If adequate retinol is provided by the diet, there are no known clinical effects of consuming diets high in carotenoids over the short term. Harmless skin discolouration in the form of carotenodermia (yellow discolouration) or lycopenodermia (orange discolouration) is the only observed adverse effect associated with the excess consumption of carotenoids from food and supplements. The condition is reversible when carotene ingestion is discontinued. The Institute of Medicine has not set any DRIs specifically for carotenoids.

7. Chemistry/Toxicology

The petitioner used data bridged from previously approved GM corn submissions to support the toxicological safety of PMI protein. The data included an acute oral toxicity study performed with mice (6 to 7 mice/sex/group) given microbially produced PMI and a bioinformatics assay using the predicted amino acid sequence of PMI. The results of these studies showed that PMI does not share amino acid sequence homology with known toxins and no adverse effects were reported in treated-mice.

Similarly, the petitioner used data bridged from previously approved GM corn submissions to support the allergenic safety of PMI protein. The data included simulated gastric fluid, simulated intestinal fluid, thermostability and bioinformatics assays. The results of these studies showed that PMI does not share significant amino acid sequence homology with putative allergens (e.g. 35% identity over 80 amino acids). PMI did share homology with an 8 amino acid sequence with frog α-paravalbumin; however, this epitope was not found to be clinically relevant as determined by an IgE screening assay. Further, PMI readily degraded

and digested under conditions normally found during food preparation and in the gastrointestinal tract. As such, intact and functional PMI protein is not expected to be systemically available or pose an allergenic health concern to consumers.

The predicted amino acid sequences of the ZmPSY1 and CRTI proteins were compared to sequences of known toxins retrieved from Uniprot Knowledgebase (550 116 sequences), Swiss-Prot (6588 sequences) and TrEMBL Uniprot Consortium databases (17 510 sequences). A single match (37% sequence homology over 81 amino acids within the N-terminal region, 30 amino acids out of 492 amino acids) was found between CRTI and a sequence in a known toxin. However, this known toxin is not an oral toxin and the sequence similarity is in a cofactor binding domain that is common in many metabolic enzymes; this domain is not responsible for the catalytic and toxic activity of the known toxin. Taken together, there is no indication that this small homology would alter the oral safety of CRTI. This argument is further supported as CRTI is readily denatured and digested as demonstrated by the in vitro heat stability and simulated gastric fluid assays, and CRTI showed an absence of toxic effect in the acute oral toxicity study. Thus, no significant matches were found between ZmPSY1 and CRTI proteins and the oral toxins listed in these databases. It was concluded that ZmPSY1 and CRTI do not share sequence similarity with known oral toxins.

GR2E rice expresses a ZmPSY1 protein that was originally derived from corn. ZmPSY1 protein is produced in the endosperm of corn kernels and facilitates the accumulation of carotenoids, which confers a yellowish or orange colour to the kernel. Farmers have selected for yellow and orange corn due its higher levels of lutein, β-carotene and β-cryptoxanthin as a source of vitamin A. By consuming these varieties, humans have had a history of safe food exposure to Z. mays with elevated levels of ZmPSY1 protein.

Due to the absence of a history of safe food exposure to CRTI, the petitioner performed an acute oral toxicity study, that was compliant with Good Laboratory Practices and Organisation for Economic Co-operation and Development (OECD) guidance, to assess the potential toxic effects of CRTI in male and female CRl:CD1 (ICR) mice. Animals (5 mice per sex per group) were administered 100 mg CRTI protein/kg b.w. or 100 mg bovine serum albumin protein/kg b.w. (control) or vehicle (buffer) administered by gavage as two doses given four hours apart. Animals were monitored for 15 days and then euthanized for necropsy. All animals survived to scheduled sacrifice. No clinical abnormalities or differences in body weight were observed between CRTI-treated animals and controls. A no observed adverse effect level (NOAEL) of 100 mg CRTI protein/kg b.w. was determined.

Children consuming up to 12.5 g rice/kg b.w./day (0.85 µg CRTI/kg b.w./day) will have CRTI exposure levels that are approximately five orders of magnitude less than the NOAEL (100 mg/kg b.w./day) reported in the acute oral toxicity study conducted with mice. This margin of exposure (MOE) for CRTI protein is sufficiently large to be protective of consumer safety.

The petitioner performed a bioinformatics analysis using the predicted amino acid sequence of the ZmPSY1 and CRTI proteins and compared it with sequences of known allergens retrieved from the AllergenOnline database (2016; 1956 sequences). The proteins did not share ≥ 35% amino acid identity with any known allergen or contain potential allergen epitopes. Based on the results of the bioinformatics analysis, it was concluded that ZmPSY1 and CRTI did not match known allergens.

The petitioner demonstrated that microbial ZmPSY1 and CRTI proteins lost enzymatic activity when incubated at temperatures equal to or greater than 50 and 55 ºC, respectively, for 15 minutes. The processing and cooking of GR2E rice products will generally require temperatures that greatly exceed 55°C which will help degrade and/or denature the ZmPSY1 and CRTI proteins in the final food product. These actions will result in a reduced amount of intact or active ZmPSY1 and CRTI in the human diet.

Microbial-derived ZmPSY1 and CRTI were found to be completely digested in simulated gastric fluid (SGF; 10 U pepsin per µg test protein; pH ~ 1.2; incubated at 37 °C) within 5 minutes and 30 seconds, respectively, as visualized by stained SDS-PAGE gel and western blot. As such, ZmPSY1 and CRTI proteins are expected to be digested under the conditions normally found in the stomach such that no intact and functional protein would be absorbed in humans to initiate an allergic response.

Conclusion:

Health Canada’s review of the information presented in support of the food use of Provitamin A Biofortified Rice Event GR2E does not raise concerns related to food safety. Health Canada is of the opinion that food derived from this event is as safe and nutritious as food from current commercial rice varieties.

The petitioner has been informed that, if in the future, there is an interest in selling this rice in Canada, compliance with the Food and Drug Regulations regarding the addition of vitamins to foods would be required. Similarly, they have been informed that, due to the increased levels of provitamin A, the common name of any food products derived from this rice would be required to differentiate this rice from conventional varieties.

Health Canada's opinion deals only with the food use of Provitamin A Biofortified Rice Event GR2E.

This Novel Food Information document has been prepared to summarize the opinion regarding the subject product provided by the Food Directorate, Health Products and Food Branch, Health Canada. This opinion is based upon the comprehensive review of information submitted by the petitioner according to the Guidelines for the Safety Assessment of Novel Foods.

(Également disponible en français)

For further information, please contact:

Novel Food Section
Food Directorate
Health Products and Food Branch
Health Canada, PL2204E
251 Frederick Banting Driveway
novelfoods-alimentsnouveaux@hc-sc.gc.ca

Supplementary Document 8 (Confined testing approval)

NCBP 1990

Republic of the Philippines
National Committee on Biosafety of the Philippines
Department of Science and Technology (DOST)-Biosafety Committee

23 February 2015

DR. NENITA V. DESAMERO
Chairperson
Institutional Biosafety Committee
Philippine Rice Research Institute (PhilRice)
Maligaya, Science City of Muñoz, Nueva Ecija

DOST-BC Ref. No. 2015-0286: Evaluation of Pro-Vitamin A enriched 'Golden Rice' Event E introgressed lines of PSB Rc82 under confined field test conditions

Dear *Dr. Desamero*:

The DOST-Biosafety Committee (DOST-BC), during its 70th meeting on 21 February 2015, approved the conduct of the above-cited experiment. This approval is subject to compliance with all the biosafety measures indicated in the proposal, the conditions enumerated in the annex of this approval letter and the additional conditions that maybe imposed by the Committee, as deemed necessary, for the duration of the experiment.

Please be advised that this project should commence within two (2) years from the date of issuance of the approval letter and should follow the activities indicated in the Gantt chart. Should the proponent fail to start the experiment within the prescribed two-year period, the proposal will have to be re-submitted for re-evaluation of the DOST-BC. Furthermore, we would like to emphasize that the proponent should submit, for endorsement of the PHILRICE-IBC to the DOST-BC, a completion report within ninety (90) days after the project has been completed. Similarly, the PHILRICE-IBC is enjoined to submit a report on all its monitoring activities upon completion of this project, through the submission of the IBC completion report.

Furthermore, kindly note that sections of the proposals that have bearing on the risk assessment and not proprietary in nature shall not be considered as confidential.

As Chairman of the PHILRICE-IBC, please assure that the IBC members are aware of their responsibility in ensuring that the biosafety measures are observed for the duration of the activity. The IBC members, as well as the staff involved should be properly trained to monitor and comply with the biosafety guidelines and conditions set by the DOST-BC. From time to time, designated DOST-BC monitors shall visit your facilities to check for compliance with all confinement and mitigation measures.

Kindly coordinate with the personnel of the Post Entry Quarantine Station of the Bureau of Plant Industry (PEQS-BPI) to finalize the monitoring schedule and for such other requirements necessary to enable you to safely conduct the activities of the above-cited proposal. Lastly, we encourage you to regularly visit the DOST-BC website (http://dost-bc.dost.gov.ph/) for announcements and modifications that may have bearing on your compliance with the biosafety requirements.

Should you have further queries, feel free to contact the DOST-BC Secretariat at (02) 837-2071 local 2012 or e-mail secretariat@dost-bc.dost.gov.ph.

Thank you for your continuing support and cooperation.

Very truly yours,

JAIME C. MONTOYA, MD, MSc., PhD, CESO III
Executive Director, Philippine Council for Health Research and Development
and Chair, DOST Biosafety Committee

c: *Dr. Antonio Alfonso (PHILRICE)*
Asec. Paz J. Benavidez II (DA-BPI)
Ms. Merle B. Palacpac (DA-BPI-PEQS)
DOST-BC Members

Filename: 2015-02-23_DESAMERO_Approval of 2015-0286 (PHILRICE-GR2-E)_M

Rm. 305, DOST Bldg. Gen. Santos Ave.
Bicutan, Taguig City, Metro Manila
E-mail address: secretariat@dost-bc.dost.gov.ph

Telefax Nos.: (632) 837-2930, 837-2943;
Trunkline: 837-2071 to 82 local 2510
URL: NCBP: www.ncbp.dost.gov.ph; BCH: http://bch.dost.gov.ph

ncbp-15-00113

NCBP
1990

Republic of the Philippines
National Committee on Biosafety of the Philippines
Department of Science and Technology (DOST)-Biosafety Committee

ANNEX

Conditions for the Approval of
DOST-BC Ref. No. 2015-0286: **Evaluation of Pro-Vitamin A enriched 'Golden Rice' Event E introgressed lines of PSB Rc82 under confined field test conditions**

a) Before commencing with the activities, the DOST-BC and the Post Entry Quarantine Station, Bureau of Plant Industry (PEQS-BPI) shall inspect and approve the proposed site for the confined test (CT) located at Philippine Rice Research Institute, Brgy. Maligaya, Science City of Muñoz, Nueva Ecija.

b) Prior to the initiation of the activities, the proponent/s shall discuss the monitoring schedule with the DOST-BC and the PEQS-BPI to identify the activities that will require their presence. The proponent/s shall furnish the DOST-BC and the PEQS-BPI with a copy of the agreed monitoring schedule.

c) The DOST-BC approved Project Information Sheet (PIS) shall be posted for three (3) consecutive weeks in at least three (3) conspicuous places in the Barangay and/or City/Municipal halls where the proposed activities will be conducted. The PIS should be posted at least fifteen (15) days before the actual transplanting of seeded transgenic rice in the confined test site. Proof of posting of the PIS, which may either be a certification from the duly authorized Barangay leader(s) or an affidavit executed by the Proponent, should be submitted to the DOST-BC within ten (10) days from the last day of posting.

d) The proponent shall adhere as closely as possible to the schedule of implementation and monitoring activities for this activity.

e) Modifications in the planting schedule and other activities may be made only with the concurrence of the IBC and DOST-BC.

f) At least 150 meters physical or temporal reproductive isolation of the GMO shall be observed at all times.

g) All confinement measures should be in place before the actual transplanting of rice seedlings.

h) The proponent shall ensure that only authorized personnel are allowed inside the test site.

i) The DOST-BC and the PEQS-BPI shall be informed in advance of any visitations by unauthorized personnel.

j) The DOST-BC and the PEQS-BPI shall be informed immediately of any intrusion by unauthorized persons.

k) The proponent shall ensure that rodent control measures are in place and that stray animals are excluded from the test site while trials are being conducted.

l) The proponent shall ensure the security of the trial, the field workers and the monitors.

m) In case of undue destruction of the experimental materials resulting from unauthorized entry of personnel or breach of containment of the confined test facility, the proponent should implement measures to prevent the inadvertent escape of any viable material within the site.

n) The proponent and the supervising IBC shall be held accountable for the undue destruction of the experimental materials and the consequences that its inadvertent escape may cause to the surrounding environment.

o) The rice grains or any plant part shall not be eaten or fed to humans, wildlife and livestock.

p) The proponent shall provide a space within the test site for disposal of the used plant materials.

Rm. 305, DOST Bldg. Gen. Santos Ave.
Bicutan, Taguig City, Metro Manila
E-mail address: secretariat@dost-bc.dost.gov.ph

Telefax Nos.: (632) 837-2930, 837-2943;
Trunkline: 837-2071 to 82 local 2510
URL: NCBP: www.ncbp.dost.gov.ph; BCH: http://bch.dost.gov.ph

q) The harvested grains shall be properly labeled and stored at the designated Seed Storage Facility where transgenic seeds are kept, while plant materials and other plant debris used within the test site should be buried in a pit within the test site or plowed under.

r) The proponent shall leave the test site fallow for at least 30 days. During the fallow period, the proponent shall strictly monitor emergence of volunteer plants and plant pests/diseases within the site for two (2) consecutive weeks after harvest. Furthermore, the proponent shall coordinate with the PEQS-BPI personnel during this period for the monitoring of the fallow site. If necessary, the proponent may irrigate the test site to induce germination of seeds. Any volunteer plants that will emerge, or any ratoon residues shall be uprooted and disposed either by plowing under or by burying in the pit intended for transgenic plant biomass from this confined field test.

s) The proponent shall comply with additional requirements that the DOST-BC may impose, as necessary, during the experiment period.

t) The proponent shall submit through the IBC a completion report ninety (90) days after the official completion of the experiment which shall be determined by the PEQS-BPI monitors after completion of all post-harvest monitoring of volunteers.

u) The IBC shall submit a report on all its monitoring activities upon completion of this project.

-Nothing follows-

ncbp-15-00113

Supplementary Document 9 (varietal registration certification)

Republic of the Philippines
Department of Agriculture
BUREAU OF PLANT INDUSTRY
National Seed Industry Council

CERTIFICATE OF REGISTRATION

issued to

'Malusog 1'

Rice

(Oryza sativa L.)

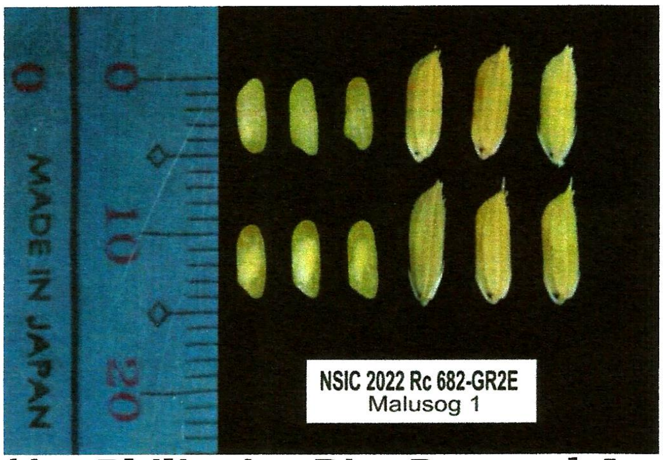

Developed by: **Philippine Rice Research Institute-
International Rice Research Institute**

**Registration Number:
NSIC 2022 Rc 682GR2E**

has complied with the requirements of the NSIC pursuant to

Republic Act 7308 of 1992 also known as the **Seed Industry Development Act** and other pertinent Laws, Rules and Regulations of the Philippines.

Scope of Registration: *National*

Issued On: *April 7, 2022*

Breeding Team:

B.P. Mallikarjuna Swamy	Severino Marundan Jr.	Anielyn Alibuyog	Raul Boncodin
Reynante L. Ordonio	Ronalyn T. Miranda	Anna Theresa Rebong	Russell F. Reinke
Marissa Romero	Helen R. Pasicolan	Antonio A. Alfonso	Roel R. Suralta
Mercy Samia			Democrito B. Rebong

GEORGE Y. CULASTE, Ph.D.
Executive Director

Republic of the Philippines
Department of Health
SINGLE JOINT RESEARCH ETHICS BOARD

SJREB FORM 4.1
CERTIFICATE OF EXEMPTION FROM ETHICS REVIEW

Date: 30 October 2020

This is to certify that the following protocol and related documents have been reviewed and granted exemption from review by the SJREB for implementation

SJREB Protocol No.:	SJREB-2020-83	Sponsor Protocol No.:	N/A

Coordinating Investigator:	Dr. Jesusa C. Beltran	Sponsor:	Philippine Rice Research Institute (PhilRice)<br>International Rice Research Institute (IRRI)

Title:	Research on Market and Consumer Acceptance of Golden Rice in Selected Provinces in the Philippines

Protocol Version No.:	Version 1	Version Date:	October 28, 2020

ICF Version No.:	Version 1	Version Date:	October 28, 2020
Other Documents:	Data Collection Tools<br>CVs and GCP Certificate of the following people: Dr. Jesusa Beltran, Ms. Fidela Bongat, Ms. Kristine Marie Daplin, Mr. Ralph Homer Ante, and Ms. Rochelle Caliwagan,		

NOTE: SJREB has exempted this protocol from ethics review in accordance with National Ethical Guidelines for Health and Health-Related Researchers since it is a market study and does not involve sensitive information

SJREB Chair	Signature	Date
Dr. Jacinto Blas Mantaring III	[signature]	03 Nov 2020

NOTE:

- Final/Closure Reports should be submitted at the end of the study.
- Any amendment to the protocol should be submitted to SJREB for re-evaluation of exemption.

Received by:

Name: ____

Signature: _____________________________________ Date: ____________________________

IRRI Research Ethics Committee Approval Form

Russel Reinke

The IRRI Research Ethics Committee has recently reviewed your responses in the conditions placed upon the ethical approval for the project listed below.

Your project has been deemed to meet the requirements of the IREC and has therefore been approved.

Protocol Code Number:	2022-0016-A-2022-14
Project title:	ASSESSMENT OF GOLDEN RICE ACCEPTABILITY IN SELECTED PROVINCES, PHILIPPINES, 2022-2023
Approval date:	December 12, 2022
Expiry date:	September 30, 2023
IREC decision:	Approved

The standard conditions of this approval are;

(a) Conduct the project strictly in accordance with the proposal submitted and granted ethics approval, including any amendments made to the proposal required by the IREC
(b) Advise the IPMO and the DDG-R via email immediately of any complaints or other issues in relation to the project which may warrant review of the ethical approval of the project.
(c) Make submission for approval of amendments to the approved project before implementing such changes
(d) Provide a ‘Progress Report’ annually for approval
(e) Provide a ‘Final Report’ when the project is complete
(f) Advise IPMO and the DDG-R via email if the project has been discontinued.

Failure to comply with the conditions of the approval may result in withdrawal of approval for the project.

Sincerely,
IRRI Research Ethics Committee

Supplementary Document 12 (farmers' testimonies, as reported in the Golden Rice newsletter)

	Stakeholder	Testimony	Source
1	Farmer/Seed grower in Bayugan City, Agusan del Sur	"Nakita ko dito sa Golden Rice ay talagang maganda siyang binhi especially sa quality at gusto ko matikman ang lasa. Sa characteristics niya, maganda ang kalidad, mahaba ang kanyang uhay. Naengganyo ako maging cooperator ng Golden Rice upang makatulong masolve yung malnutrition ng mga bata, one way na magsupport ako sa Golden Rice upang matulungan ko ang government na mabawasan ang kaso ng malnutrition,"<br><br>[As I have observed, Golden Rice has a good quality of seeds, and I want to taste it already. It also has good characteristics in terms of its elongated stem. I was encouraged to be a cooperator of Golden Rice to help the government minimize the case of malnutrition].	Article title: Pilot provinces reap Golden Rice<br><br>Golden Rice E-Newsletter October 2022 | Issue No. 10 of 2022<br><br>https://us2.campaign-archive.com/?u=831d5b3f7694549624621422c&id=44e7646288
2	Farmer cooperators in Virac, Catanduanes	I was encouraged to plant the Malusog Rice because according to the flyers that I have read from PhilRice, it is nutritious and so I tried it. The crop's performance was good as well as its harvest,"	Article title: Malusog Rice farm walk held in Catanduanes<br><br>Malusog Rice E-Newsletter April 2023 | Issue No. 4 of 2023<br><br>https://us2.campaign-archive.com/?u=831d5b3f7694549624621422c&id=cccc11c976
3	Farmer cooperator from San Andres, Catanduanes	"The planting process of the Malusog Rice is just the same as the ordinary rice. I was encouraged to plant it because it would help the malnourished children in our community,"<br><br>"What motivated me to plant Malusog Rice is the prevalence of malnutrition in our locality. As I learned about its beta carotene content, I was encouraged. Also, because of the Malusog Rice techno-demo, I proved and showed to	

		the other farmers the good performance of the Malusog Rice.”	
4	Farmer-cooperator, Quirino	“Madali lang magtanim ng Malusog Rice. Wala naman ako naging malaking problema dahil wala naman pagkakaiba sa pagtatanim ng ordinaryong bigas (It's easy to grow Malusog Rice. I didn't have a big problem because there is no difference with growing ordinary rice)," <br>“Because it’s the same as ordinary rice, we use the same treatments for any planting issues that we experience," <br>This is the second time he has planted Malusog Rice, and he said that the natural beta carotene it contains encouraged him to support it.	Article title: Malusog Rice harvesting in Quirino province <br>Malusog Rice E-Newsletter April 2023 | Issue No. 4 of 2023 <br>https://us2.campaign-archive.com/?u=831d5b3f7694549624621422c&id=cccc11c976
5	Farmer Cooperator, Ilocos Norte	One of the farmer-cooperators who planted Malusog Rice for the first time shared that this new type of rice is easy to grow. Expressing his support for the project, he said “This is good because it is also nutritious and I hope the Malusog Rice Project continues.”	Article title: Second planting of Malusog Rice reaps 8.3t/ha this May, new yield record set <br>Malusog Rice E-Newsletter May 2023 | Issue No. 5 of 2023 <br>https://us2.campaign-archive.com/?u=831d5b3f7694549624621422c&id=87388f7f1a
6	Farmer cooperator, Catanduanes	“When it came to planting Malusog Rice, the farmer cooperator approached it with the same dedication he had for any other crop. However, the results were extraordinary. His harvest far exceeded the anticipated average yield of 5t in the area.” <br>“In a recent Malusog Rice ceremonial farm walk and briefing, he had the opportunity to share his journey with fellow farmers and members of the	Article title: Farmer cooperator in Catanduanes surpasses expected Malusog Rice yield <br>Malusog Rice E-Newsletter May 2023 | Issue No. 5 of 2023 <br>https://us2.campaign-a

		community. Expressing his gratitude for being a beneficiary of the Malusog Rice planting program, he also emphasized the simplicity of the planting process, stating that it closely resembled conventional rice farming methods. He also emphasized the significance of the program in addressing malnutrition and the importance of supporting initiatives that aim to uplift both individuals and communities."<br><br>"He also emphasized the simplicity of the planting process, stating that it closely resembled conventional rice farming methods."	rchive.com/?u=831d5b3f7694549624621422c&id=87388f7f1a
	Farmer cooperator in Batac, Ilocos Norte.	The farmer-cooperator was still very happy with his harvest of 3.8t/ha, which was higher than his previous yield. "Even if the area is saline, my harvest was still good when I planted Malusog Rice. I am grateful to PhilRice who made us aware of the condition of the field which is a great help to us. Now we know that we should plant saline-tolerant varieties to have high yield," the farmer said.	Article title: Malusog Rice can also thrive in a saline area<br><br>Malusog Rice E-Newsletter June 2023 | Issue No. 6 of 2023<br><br>https://us2.campaign-archive.com/?u=831d5b3f7694549624621422c&id=3e578ded7a

Malusog Rice

Bawat butil, puno ng sustansiya

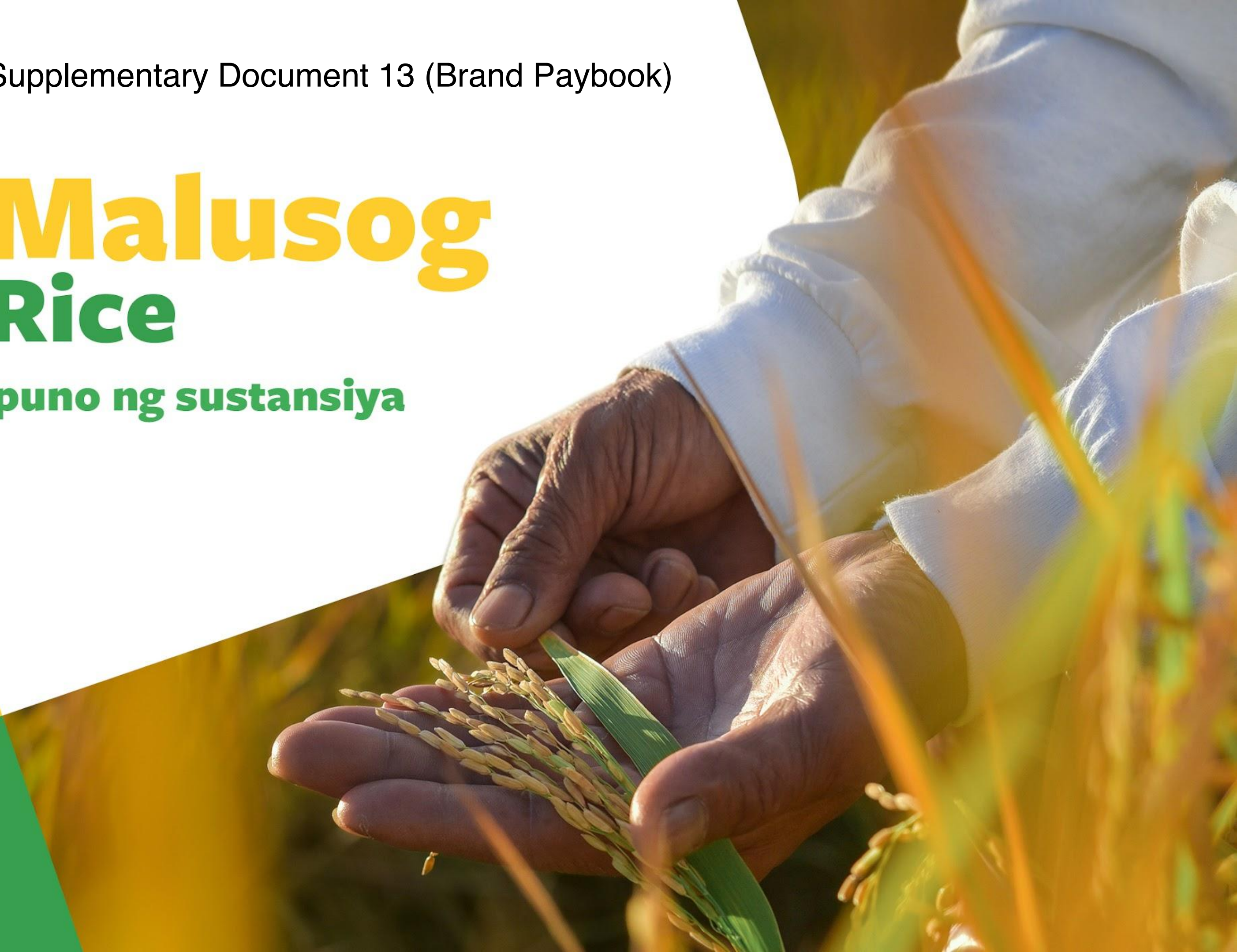

Brand Book

Table of Contents

I. **Overview**
   - A. Target Audience
   - B. Key messaging

II. **Brand Identity**
   - A. Vision, Mission, Values
   - B. Value proposition

III. **Visual Guidelines**
   - A. Logo
   - B. Color Palette
   - C. Typeface
   - D. Guidelines for use

IV. **Communication Guidelines**
   - A. Brand Persona
   - B. Brand Voice
      - 1. Language
      - 2. Tone
      - 3. Purpose

V. **Templates**

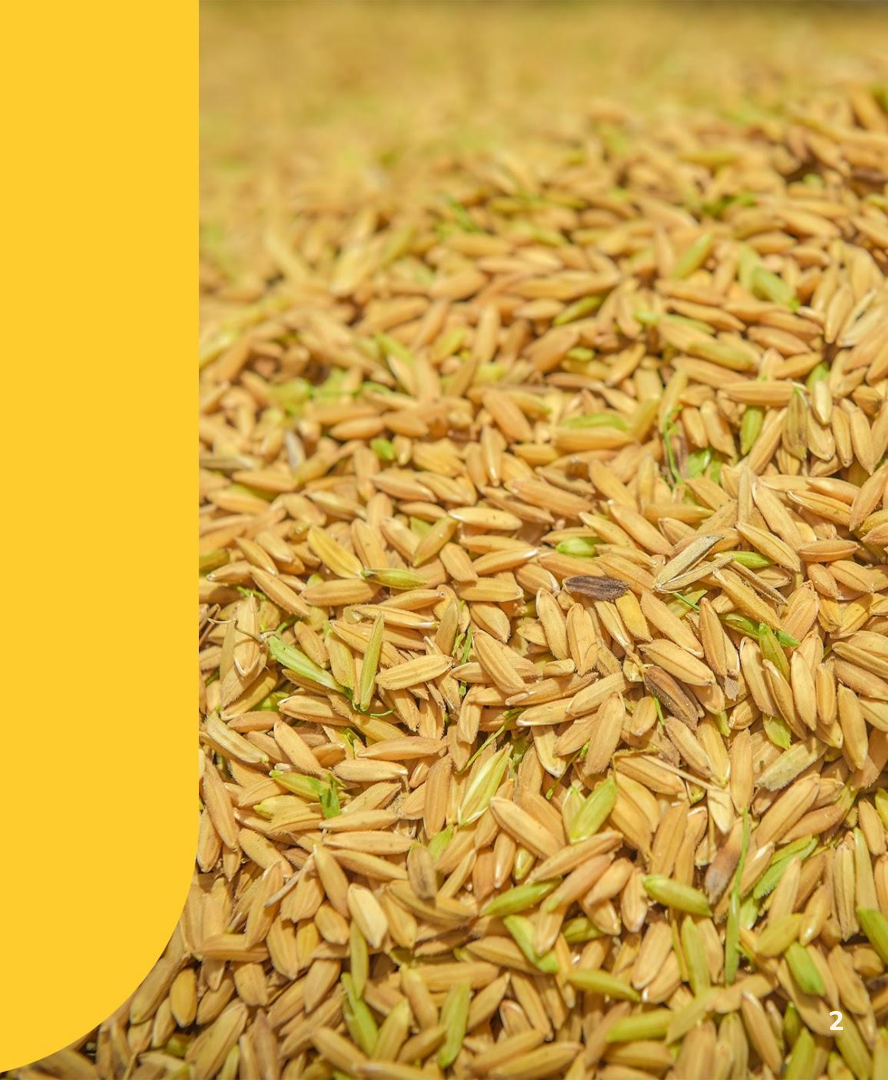

Preface

What is this brand book for?

This brand book serves as a reference material to ensure consistency in all internal and external communication materials developed for Malusog Rice. This document covers the conceptual aspects of the brand such as the mission, vision, and values statements as well as the visual and linguistic aspects, including the logo and communication guidelines.

Who is this document for?

This document is intended for the IRRI and DA PhilRice as well as their partners and other relevant stakeholders to use as reference. This is not meant to be distributed to the general public.

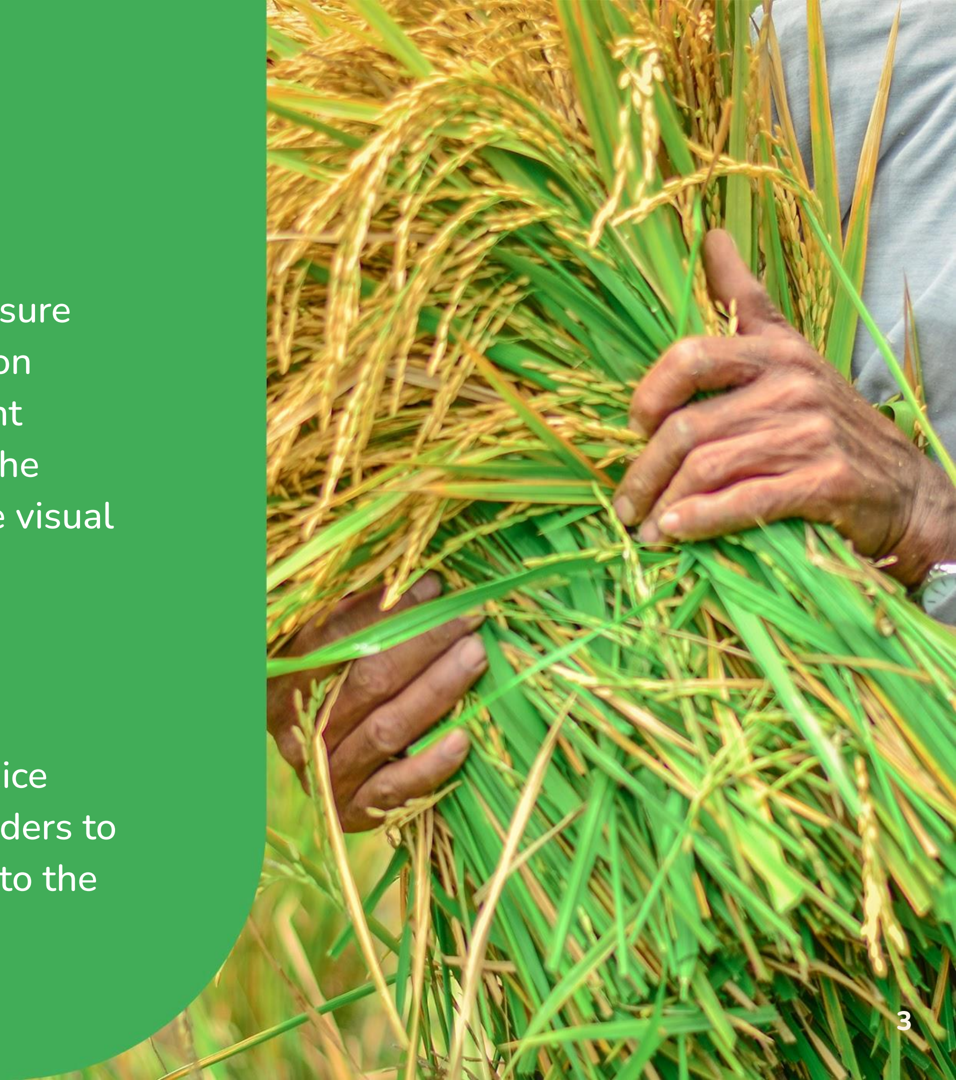

Overview

Overview

Golden Rice is a new type of rice that contains beta-carotene, a plant pigment that the body converts into Vitamin A as needed. It is the first rice variety developed to address the nutrition issue of Vitamin A deficiency (VAD).The Department of Agriculture has approved Golden Rice for commercial propagation in July 21, 2021, which makes the Philippines the first country to do so.

Philippine Rice Research Institute, Department of Agriculture (PhilRice) is leading the development of Golden Rice in the Philippines in partnership with the **International Rice Research Institute (IRRI)**. The project proponents are working closely with government agencies and organizations in the sectors of agriculture and nutrition to develop, test, and promote Golden Rice as a potential new food-based approach to improve Vitamin A status of the country, especially in malnourished and Vitamin A deficient communities.

The product branding developed is specific to the seeds and milled rice grains produced and distributed under the biosafety approval of the Department of Agriculture and partner agencies. These grain products will be marketed as **"Malusog Rice."**

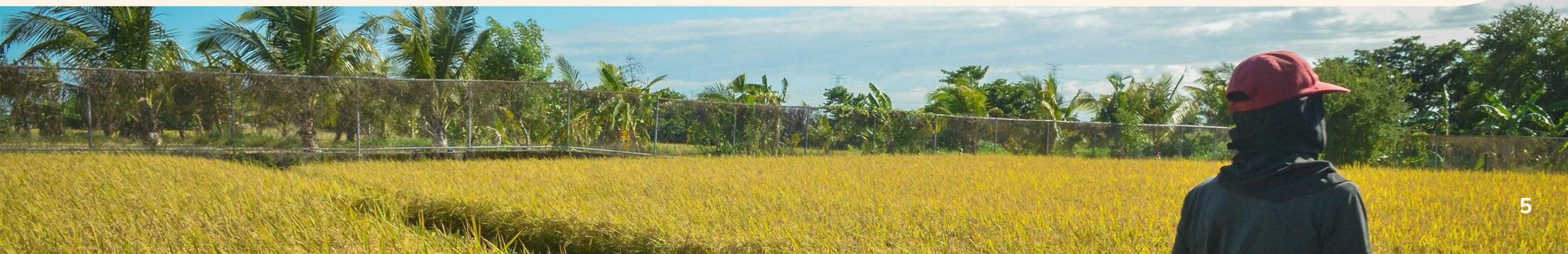

Target Audience

FARMERS are critical to the success of Malusog Rice as it is their work that allows for the propagation and production of the crop for public consumption.

CONSUMERS particularly mothers from most-at-risk populations, are the first market base that need to adopt Malusog Rice and integrate in their families' — especially their children's — diets.

Overview

Target Audience

Neutral to Opposition

Mang Ernesto has been a rice farmer for over 30 years. He comes from a family line of farmers from his father to his grandfather. He has a wife and three children who also help him in the fields.

Ernesto knows about Malusog Rice (formerly Golden Rice) through the pamphlets he has received from its proponents as well as from advocacy and interest groups. He is skeptical about the claims made for the rice variety and feels like his livelihood is being threatened by its introduction. He has a lot of questions about yield, pest-resilience, costs, and cross contamination but does not know whether the answers are reliable.

He is open to learning more about the crop through actual experience before deciding whether he will pursue this as a viable alternative to the varieties he is already familiar with.

Ernesto, 53 years old
Rice Farmer

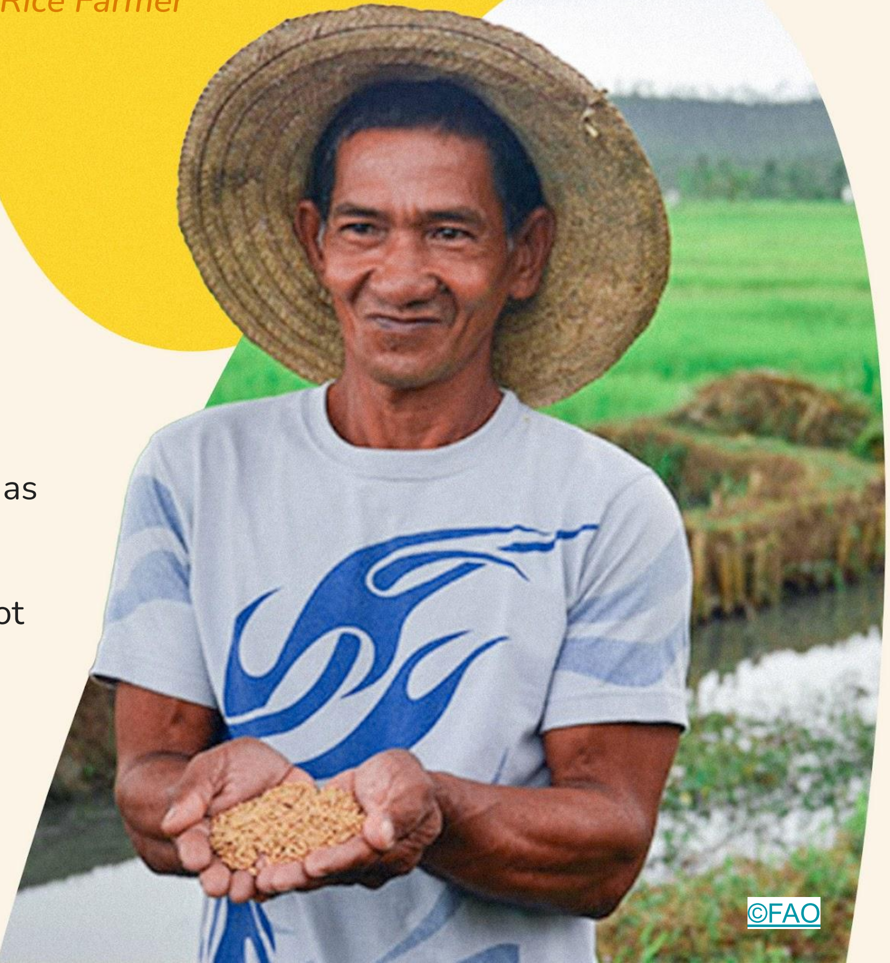

Overview

Target Audience

Neutral to Ally

Rowena is a mother of 4 children, her eldest being 7 years old and her youngest being 2 years old. Her husband works as a construction worker and has irregular income. She earns money by doing laundry and ironing for her neighbors.

She takes care of maintaining the household and is in charge of her family's daily meals. As much as possible she makes sure that they get to eat 3 meals a day even if some of these meals will only consist of rice.

Her middle children aged 4 and 5 often get sick and she heard that this is due to vitamin deficiency in their diets. She hears products such as instant milk, noodles, and canned goods promising to address this but has not heard of Malusog Rice (formerly Golden Rice) yet.

Rowena, 29 years old
Mother

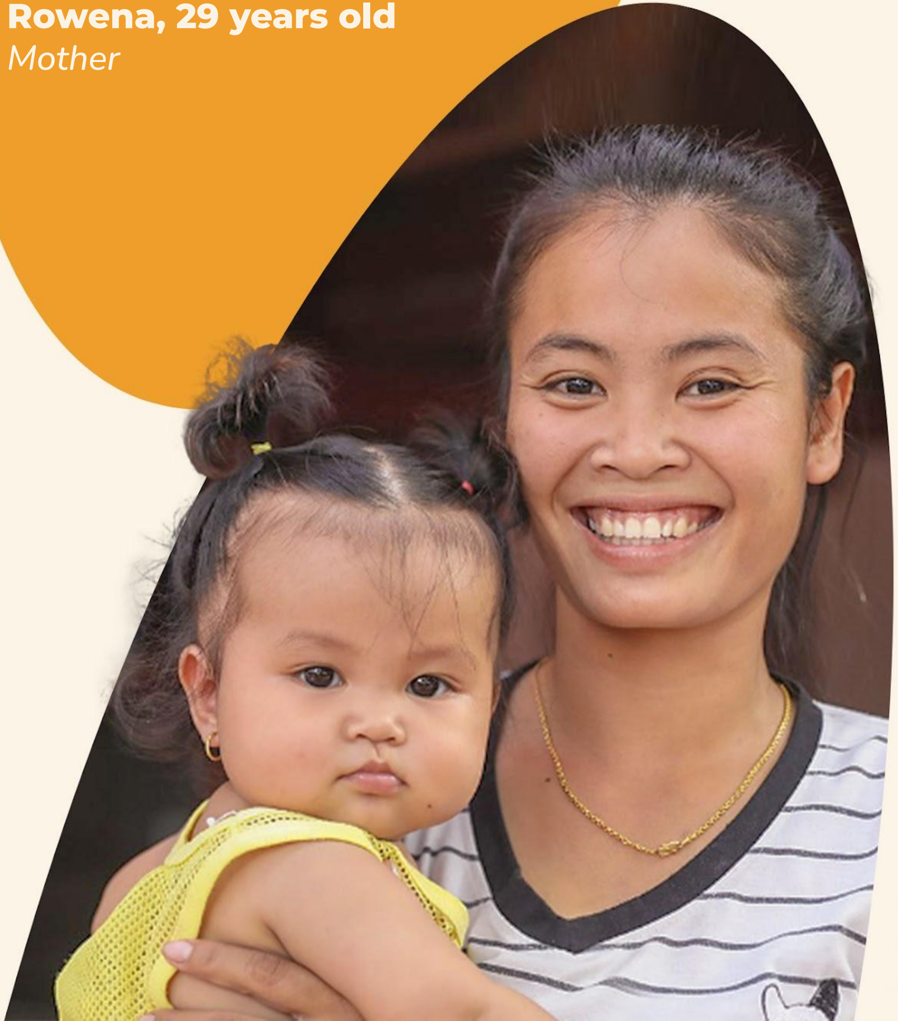

Key Messages

Source of Income

Malusog Rice provides farmers the opportunity to diversify their crops and increase their earning potential by supplying a unique product to a new market.

Source of Nutrients

Malusog Rice is a good source of Vitamin A which is essential to prevent vision loss and maintain good eyesight and a healthy immune system, especially in children under the age of 5.

Safe and Sustainable

Malusog Rice has undergone decades of scientific research ensuring its safety for consumption and sustainability for propagation in rice fields in the Philippines.

Communication Objective

Position **Malusog Rice** as a viable, safe, and sustainable alternative to popular rice varieties to encourage adoption in commercial propagation and public consumption

Overview

Communication Objectives

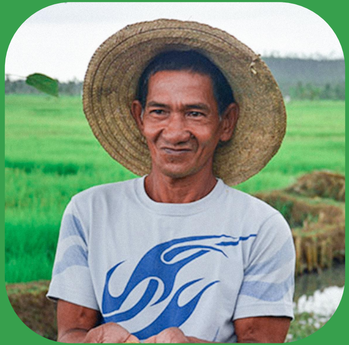

Persuade Ernesto to avail of the free Malusog Rice seeds and plant it in his field for harvest and supply to the market.

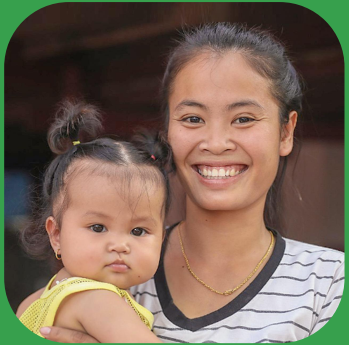

Introduce Malusog Rice to Rowena as a Vitamin A food source that can help address the issue of malnutrition in her family.

Brand Identity

Product Vision and Mission

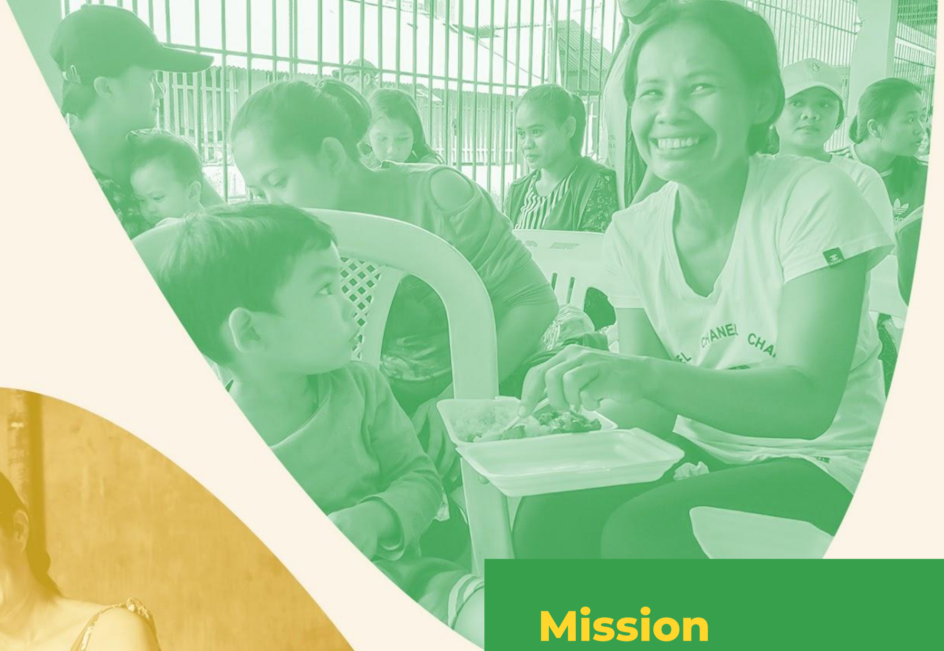

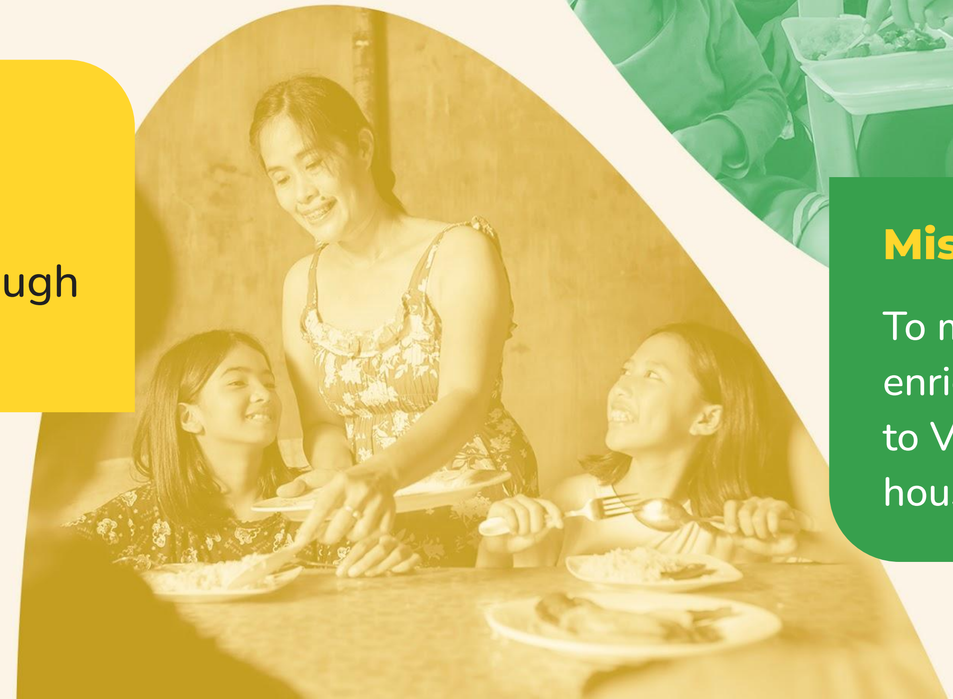

Vision

Filipino families made healthy through proper nutrition.

Mission

To make beta-carotene enriched rice accessible to Vitamin A deficient households.

Product Values

Malusog Rice is...

**Healthy and Nutritious**	Enriched with beta-carotene as an efficient source of Vitamin A
**Safe**	Developed through decades of careful scientific research to effectively address Vitamin A deficiency
**Accessible**	Propagated and distributed under humanitarian use to make the product and technology available to farmers and Vitamin A deficient communities
**Sustainable**	• Requires no use of new farming implements or techniques and suited for Philippine climate, environment and agricultural practices<br>• Complements existing community nutrition programs

Value Proposition

Healthier Filipino families through accessible, nutritious food products developed through decades of research

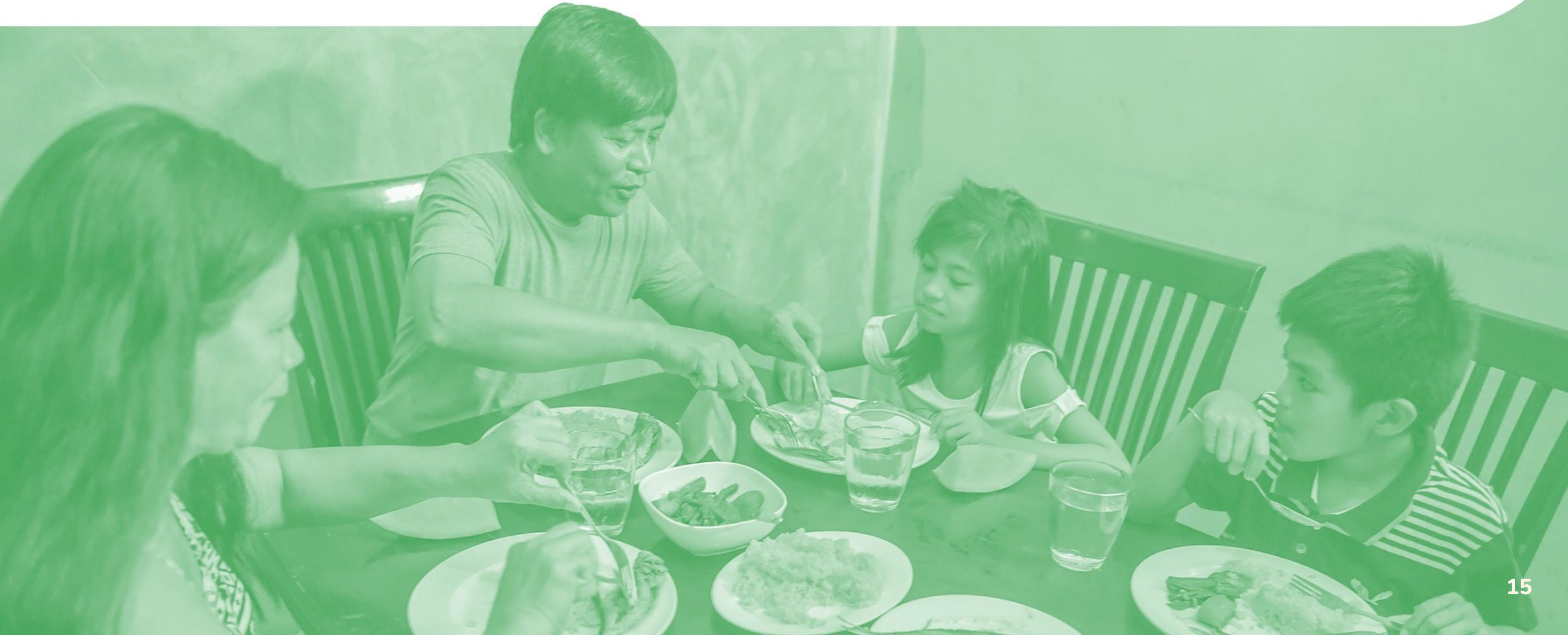

Visual Guidelines

Logo

The **Malusog Rice logo** is a visualization of the commitment to providing proper nutrition to Filipino families through beta-carotene enriched rice.

Design Elements

- Illustration of family with beta-carotene enriched rice grain
- “Malusog Rice” brand name
- Tagline (Bawat butil, puno ng sustansiya)

Malusog Rice

Bawat butil, puno ng sustansiya

Primary Lockup (Horizontal), Full Color

Logo

Variations (Lockups)

The **horizontal version** (as seen in the previous slide) is the logo's Primary lockup. Please use it as often as possible.

In vertical (or square) spaces, use the **stacked version** shown on this slide to maximize the space.

Malusog Rice

Bawat butil, puno ng sustansiya

Secondary Lockup (Stacked), Full Color

Logo

Variations (Colors)

The brand logo should be used in its full colors *whenever possible*. In instances wherein printing capabilities are limited, however, these black-and-white (B&W) versions may be used.

Malusog Rice
Bawat butil, puno ng sustansiya

Malusog Rice
Bawat butil, puno ng sustansiya

Malusog Rice
Bawat butil, puno ng sustansiya

Malusog Rice
Bawat butil, puno ng sustansiya

B&W

Logo

Sizing & Placement

1 When placed on colored background, the full-colored logo may be enclosed in a white container to ensure readability and clarity.

2 If the full-colored logo is to be used on photos, the image–or the part of the image on which the logo is to be placed–should not be too busy.

1

2

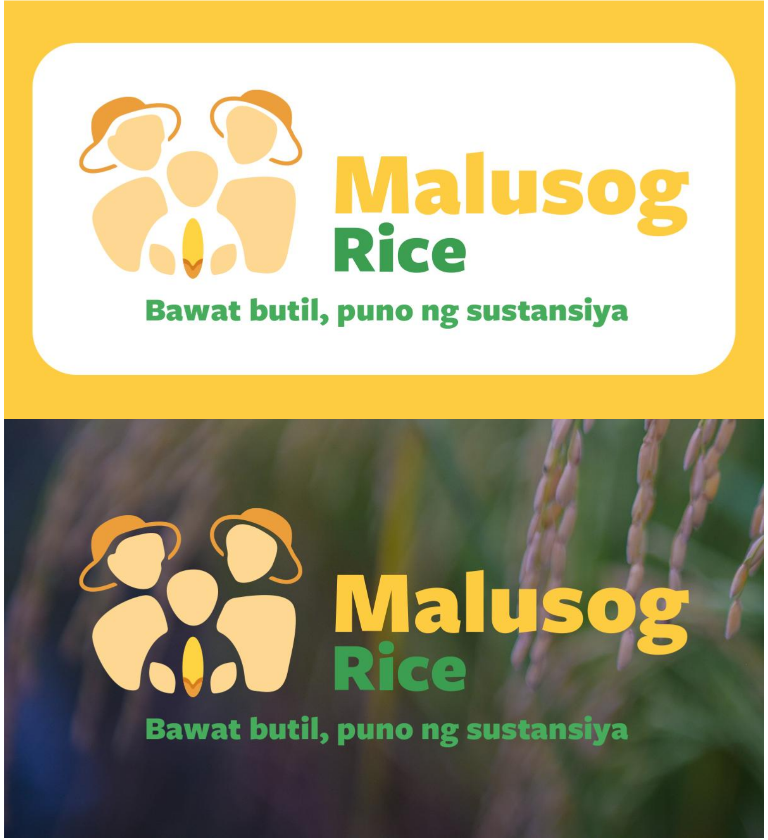

Logo

Sizing & Placement

1 The minimum free space margin around the logo is equivalent to the height of the smallcase ‘o’ in ‘Malusog.’ No outside element should encroach on this space.

2 The logo cannot be reproduced in anything below 547 px for web and 50 mm for print (width).

1

Free space

2

Minimum size: 547 px / 50 mm (width)

Logo

Violations

These are some examples of ways for the logo to be misused or misapplied.

Do not stretch or alter in any way

Do not use colors other than those specified in the guidelines

Do not overlay logo on backgrounds where it is unreadable

Do not add embellishments (drop shadow, glow, outline)

Do not screen; always print at 100% ink density

Do not omit any marks

Color Palette

Application

1 The brand's **Primary colors** are Green and Yellow, a combination that symbolizes health and livelihood, and Malusog Rice's distinctive golden color. They shall be the most prominent colors of the Brand.

2 These are complimented by a **secondary palette** consisting of shades of ochre; to be used in materials that require more variety and dynamism.

Primary

1

#379f4d
R: 55/159/77
C: 78/13/96/1

#fecc41
R: 254/204/65
C: 0/18/91/0

2

#fed892
R: 254/216/146
C: 0/15/51/0

#ec9e3b
R: 236/158/59
C: 4/42/94/0

#d97c1f
R: 217/124/31
C: 12/59/100/1

#fbf4e6

#212121

#d9d9d9

#595959

Secondary

Visual Guidelines

Color Palette

Application

3 The **Neutrals** 'ground' the vibrant color palette, ensuring clarity and readability amidst all the colors. For example, Black should be used for running text; White can be background for huge chunks of text; Gray is used for buttons, arrows, and secondary elements.

4 When the specific shades of the Brand are unavailable (such as when printing capabilities are limited), ordinary black and white may be used.

Primary

#379f4d
R: 55/159/77
C: 78/13/96/1

#fecc41
R: 254/204/65
C: 0/18/91/0

#fed892
R: 254/216/146
C: 0/15/51/0

#ec9e3b
R: 236/158/59
C: 4/42/94/0

#d97c1f
R: 217/124/31
C: 12/59/100/1

Secondary

#fbf4e6 **#212121** 3

#d9d9d9 **#595959**

Neutral

#ffffff **#000000** 4

This is Neutral White

Visual Guidelines

Color Palette

Application

Please observe color harmony and readability when pairing colors together, i.e. do not pair shades that would clash and make text and elements unreadable.

✖ Clashing

As much as possible, please stick to the color combinations provided in the color chart on the right.

Sample Text

1

Contrast Matters

- Form
- Readability

Sample Text

1

Contrast Matters

- Form
- Readability

Sample Text

1

Contrast Matters

- Form
- Readability

Sample Text

1

Contrast Matters

- Form
- Readability

Sample Text

1

Contrast Matters

- Form
- Readability

Visual Guidelines

Typography

Fonts

Montserrat and **Nunito** are the only typefaces to be used when representing the Brand.

Montserrat Extrabold

Aa Bb Cc Dd Ee Ff Gg Hh Ii Jj Kk Ll Mm Nn Oo Pp Qq Rr Ss Tt Uu Vv Ww Xx Yy Zz
0123456789 -=,./;'[]\!@#$%^&*()_+{}|:"<>?

+

Nunito Regular

Aa Bb Cc Dd Ee Ff Gg Hh Ii Jj Kk Ll Mm Nn Oo Pp Qq Rr Ss Tt Uu Vv Ww Xx Yy Zz
0123456789 -=,./;'[]\!@#$%^&*()_+{}|:"<>?

Nunito Regular It

Aa Bb Cc Dd Ee Ff Gg Hh Ii Jj Kk Ll Mm Nn Oo Pp Qq Rr Ss Tt Uu Vv Ww Xx Yy Zz
0123456789 -=,./;'[]\!@#$%^&()_+{}|:"<>?*

Nunito Extra Bold

Aa Bb Cc Dd Ee Ff Gg Hh Ii Jj Kk Ll Mm Nn Oo Pp Qq Rr Ss Tt Uu Vv Ww Xx Yy Zz
0123456789 -=,./;'[]\!@#$%^&*()_+{}|:"<>?

Typography
Usage

For titles and headings, **Montserrat Extra Bold** is the only font style to be used. Please make sure that the font size is at least 1.5x bigger than the body text.

For subheadings and body text, use **Nunito**.

Headings/Title
Montserrat ExBold

Sa dagat at bundok, sa simoy at sa langit mong bughaw

Subheadings
Nunito Regular It

Lorem ipsum dolor sit amet, consectetur adipiscing elit

Body
Nunito Regular
+
Nunito ExBold,
Montserrat ExBold

Nulla convallis, tortor eget pretium elementum, diam nulla laoreet nulla, et tincidunt elit mauris in felis. This is a word being **emphasized**. It is set in **Nunito Extra Bold** Vivamus vitae euismod sem. Donec dui lectus, pellentesque et augue vel, pretium congue ligula:

- **1 out of 3** is an important number
- Pellentesque et porta massa

Visual Guidelines

Photography and Visual Style Guide

IRRI & DA PhilRice's asset bank consists of documentation of their events and activities throughout the country.

Assets should show farmers, consumers, and the people involved in the process of making and consuming Malusog with joyful expressions. Images should show them in action shots. All images should depict realistic scenarios while maintaining dignity in framing the subjects. Ensure that the subjects are framed positively, empowered, and proud.

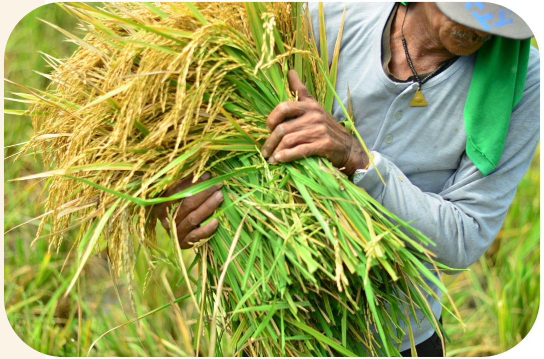

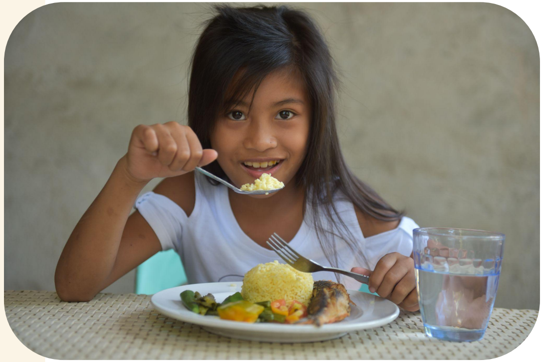

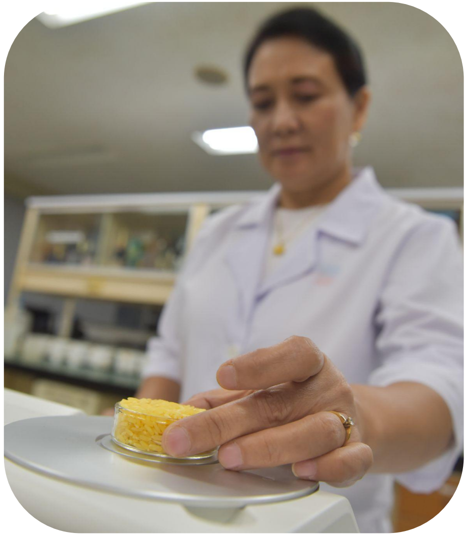

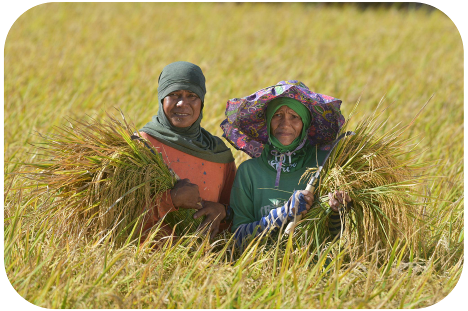

General Photo and Video Guidelines

- **Mind your angles** - In the audience's eyes, the camera angle represents their visual perspective; photos of human subjects should be taken from a low or eye-level angle to portray equality and respect. Conversely, taking photos and videos from a higher angle display subject as being looked down upon.
- **A face to your campaign** - Showing the subject's face allows the audience to connect with the subject. Other elements in the photo and video provide information to the audience while the subject's face give the emotion.
- **Genuine but not Disparaging** - It is important to show authenticity in your photos and videos but remember to be truthful without being disrespectful. They should accurately represent the people and their community while ensuring that the subjects are portrayed with dignity.

General Photo and Video Guidelines

- **Display Diversity** - Whenever possible, ensure that the subject pool is diverse, conscious of gender, age and other representations. Communicate how the brand honors inclusivity and diversity through photo and video materials.
- **Document Consent** - Obtain the subject's consent through formal documentation. Ensure that they are informed of their right to consent or to refuse and that they fully understand the terms to which they consent.

Photo and Video Guidelines with Children

- Ensure local traditions, laws, or restrictions for reproducing personal images are adhered to before photographing or filming a child.
- Obtain informed consent from the child and parent or guardian of the child before photographing or filming a child. An explanation of how the photograph or film will be used must be provided.
- Ensure photographs, films, videos and DVDs present children in a dignified and respectful manner and not in a vulnerable or submissive manner. Children should be adequately clothed and not in poses that could be seen as sexually suggestive.
- Ensure images are honest representations of the context and the facts.
- Ensure file labels, meta data or text descriptions do not reveal identifying information about a child when sending images electronically or publishing images in any form.

Communication Guidelines

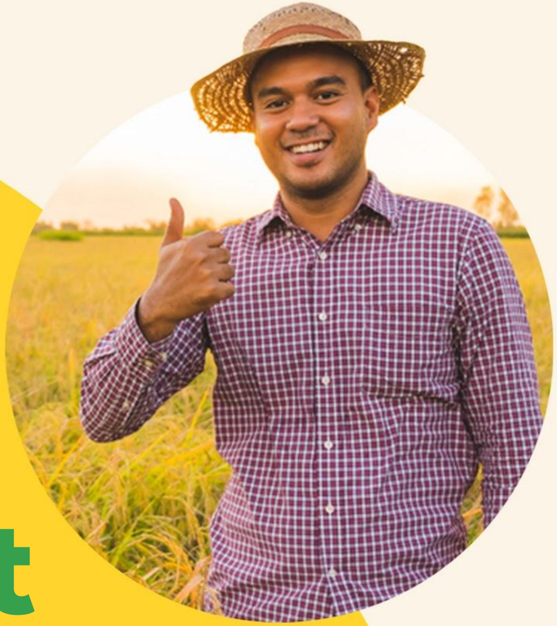

Meet Manuelo

Brand Persona

Manuelo is a young agriculturist and comes from a family of farmers from Laguna. He knows the science, practice, and management of agriculture and agribusiness. More importantly, he knows that people are at the heart of agriculture.

Manuelo is not only knowledgeable but he is also approachable, nurturing, and inclusive. He knows how to make complicated information understandable and less intimidating. He doesn’t use jargon when he speaks.

A brand persona humanizes your business and creates a story that your customers can connect with. - [Forbes.com]

Notes on the Brand Persona

The brand persona is how your brand is humanized as a tool to help you develop your communication materials. Often it is a fictitious character that embodies your brand if it were to become a person.

The brand persona is different from your brand ambassador or spokesperson. However, they should be guided by the language, tone, and voice of your brand.

Brand Voice

Language

- Conversational English and Tagalog
- Informative but not technical

Tone

- Inclusive and inviting
- Friendly but not informal
- Functional yet expressive

Purpose

- Inform
- Invite
- Encourage
- Persuade

Sample Statement: "Ang Malusog Rice ay pinalakas ng beta-carotene na nakatutulong sa pag-absorb ng Vitamin A sa katawan para sa mas matibay na resistensya."

Considerations

While laymanizing information about or relating to Malusog Rice is encouraged, it is also important to note that terms and phrases need to accurately represent or explain the science behind the product.

For example “Beta-carotene enriched” is used instead of “Vitamin A enriched” as the latter misrepresents the product. The product contains beta-carotene, provitamin A, a plant pigment that the body converts into Vitamin A as needed. The rice itself is not enriched with Vitamin A.

Such considerations are important before any communication material is finalized for production and distribution.

Templates

Social Media Posts

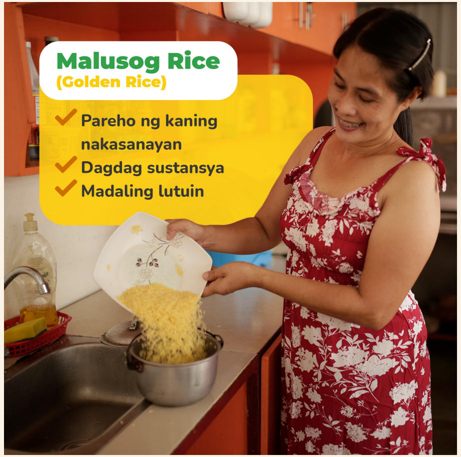

Text + Photo
(Both as main subjects)

- Text container/enclosure for readability
- Foreground element may overlap text container, **but** should not cover or interfere with text

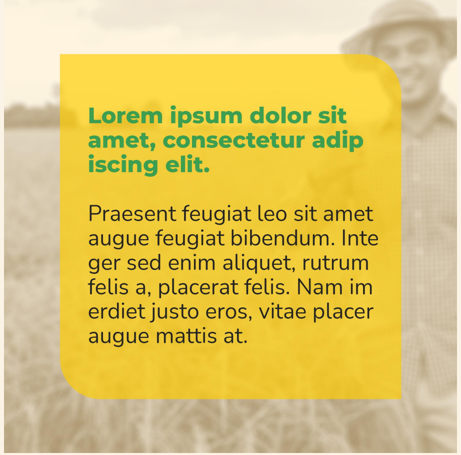

Text-heavy

- Text container/enclosure for readability
- Background may display a supporting image set in low opacity

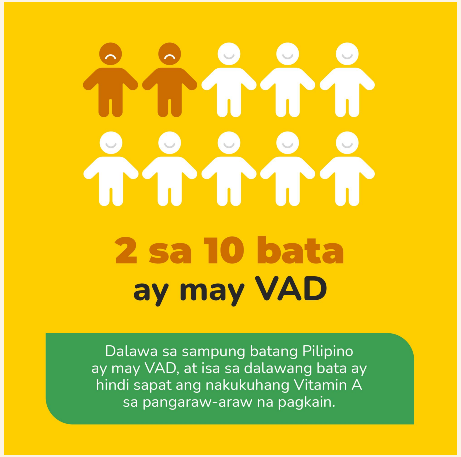

Illustration
(Infographic-type posts)

- Simple and friendlier shapes (less sharp corners, curvier characters)

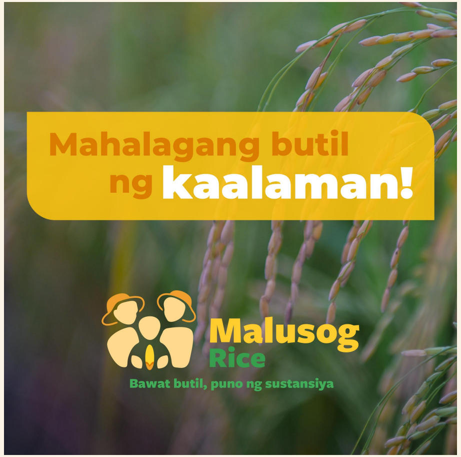

Title Cards
(For videos, photo sets)

- Text container/enclosure for readability
- Logo is displayed in full color (for logo placement on non-white and non-plain background, please see page 20 of this document)

Templates

Print Poster

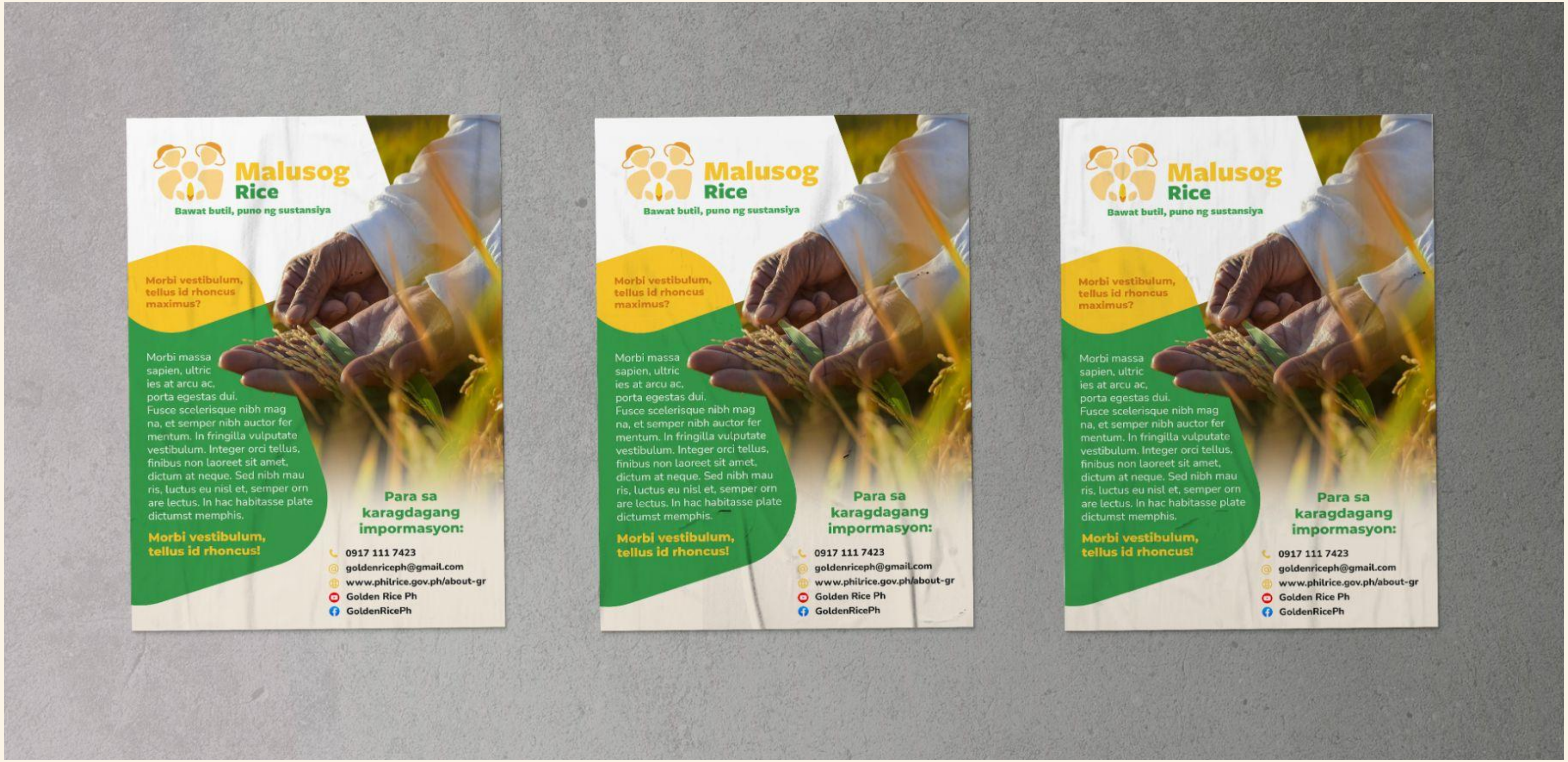

Mockup

Brochure

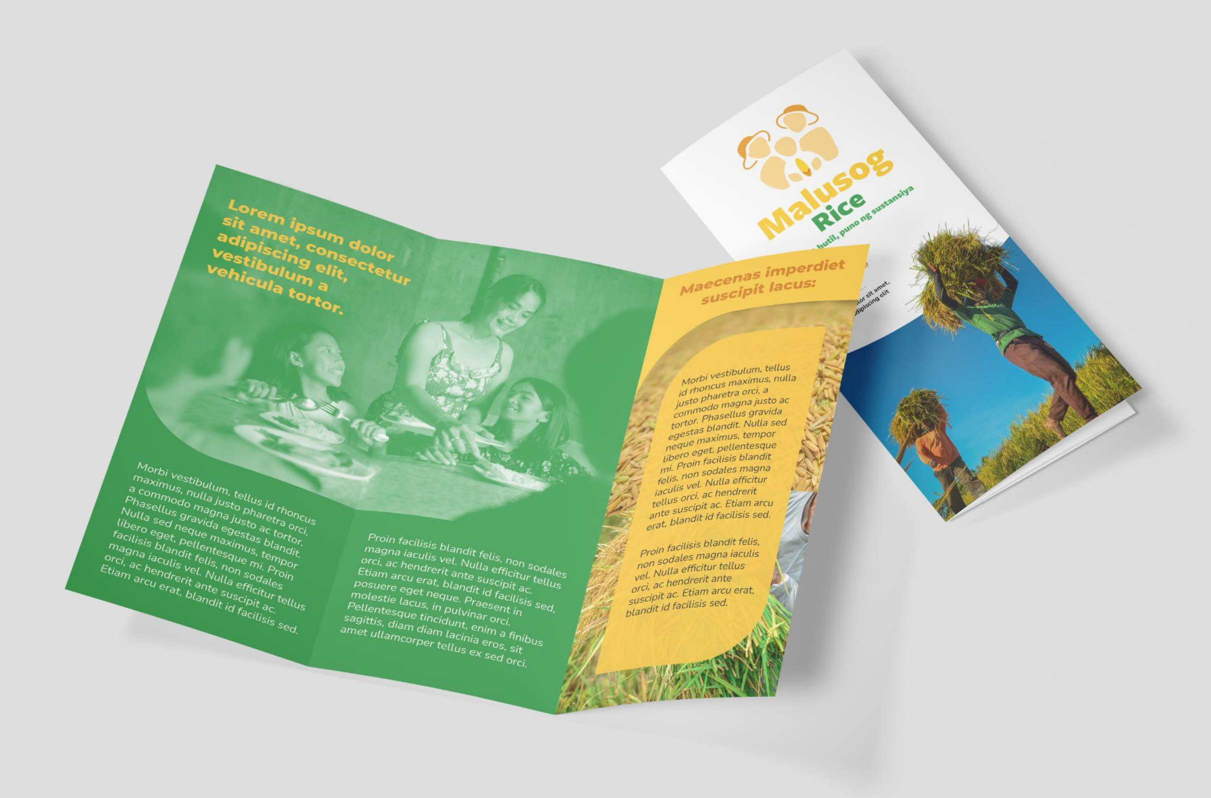

Mockup

Thank You!

Supplementary Document 14 (samples of posters, banners and other information materials)

Kahalagahan ng

Vitamin A

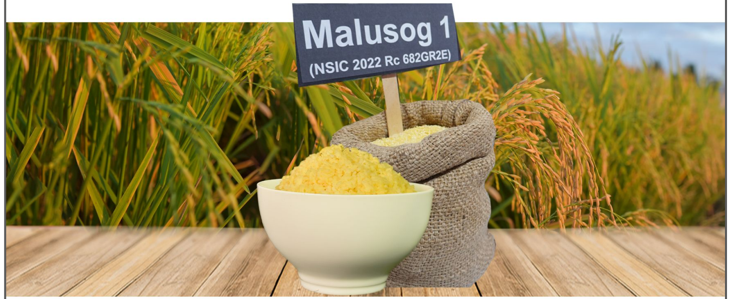

Ano ang
Vitamin A

Ang vitamin A ay isa sa mga pangunahing sustansya na kinakailangan ng katawan. Ito ay makukuha sa iba't-ibang uri ng pagkain gaya ng karne, manok, isda, gatas, carrot, kamatis, kalabasa, at iba pang berde at madahong gulay.

Ano ang
Malusog Rice Program

Ang Malusog Rice Program ay inilunsad upang maihatid ang Malusog Rice sa mga mamimili at magsasakang pilipino. Saklaw ng programa ang pagtatanim, pagpaparami at pagpapalaganap ng binhi, pag-aani ng bigas, at pagkonsumo nito.

Mga benepisyo ng
Vitamin A

- Malinaw na paningin
- Maayos na paglaki at pag-iwas sa pagkabansot
- Pagsulong sa pagtubo ng mga buto
- Resistensya sa sakit at kaligtasan sa kamatayan, lalo na sa mga bata

www.philrice.gov.ph/golden-rice GoldenRicePh GoldenRicePh GoldenRicePh

---

Malusog Rice

Bawat butil, puno ng sustansiya

Kanin na may likas at natural na sustansya

Ang Malusog (Golden) Rice ay bagong uri ng bigas na may likas at natural na beta carotene na nagiging vitamin A ayon sa pangangailangan ng katawan.

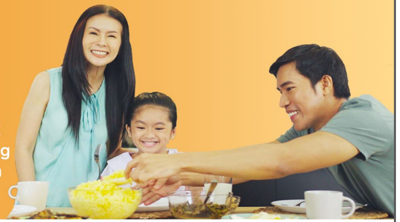

Mayaman sa beta carotene

Text

Ang beta carotene ang nagbibigay ng dilaw na kulay sa Malusog Rice. Katulad din ito ng beta carotene na taglay ng mga dilaw/orange na gulay tulad ng kalabasa at carrots. Mayroon ding beta carotene sa mga berde at madahong gulay tulad ng malunggay at kangkong.

Balanseng pagkain

Mas mainam kung kainin ito tuwing almusal, tanghalian, at hapunan, kasabay ng mga pagkaing mayaman sa iba pang sustansya alinsunod na rin sa rekomendasyon ng Pinggang Pinoy ng Department of Science and Technology - Food and Nutrition Research Institute (DOST-FNRI).

Produkto ng genetic engineering

Ang Malusog Rice ay produkto ng genetic engineering(GE) upang maisalin ang beta carotene sa butil nito. Mga karaniwang produkto rin ng GE ay kagaya ng tokwa, toyo, mais, bakuna, insulin at iba pa.

Isang tasa, puno ng sustansya

Ang isang tasa ng Malusog Rice ay makabagbibigay ng 30-50% ng Estimated Average Requirement (EAR) pangangailangan ng vitamin A ng mga bata, buntis o nagpapasusong ina.

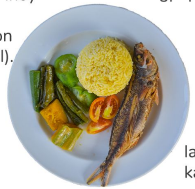

Gaya rin ng ordinaryong bigas

Base sa resulta ng mga naunang pagpapatikim ng Malusog Rice, pareho lamang ang lasa, amoy, at lambot nito sa ordinaryong kanin.

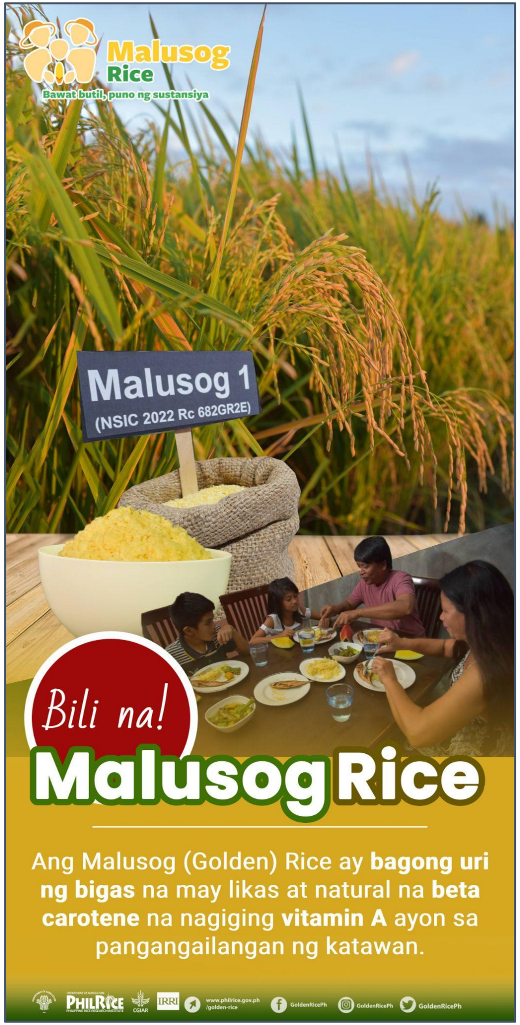
Malusog Rice
Bawat butil, puno ng sustansiya
Malusog 1
(NSIC 2022 Rc 682GR2E)
Bili na!
Malusog Rice
Ang Malusog (Golden) Rice ay bagong uri ng bigas na may likas at natural na beta carotene na nagiging vitamin A ayon sa pangangailangan ng katawan.
PHILRICE
IRRI
www.philrice.gov.ph /golden-rice
GoldenRicePh
GoldenRicePh
GoldenRicePh

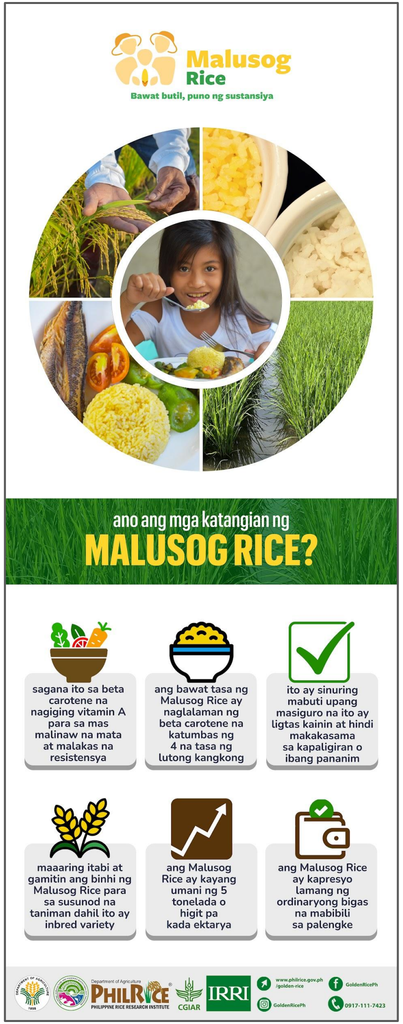
Malusog Rice
Bawat butil, puno ng sustansiya
ano ang mga katangian ng
MALUSOG RICE?
sagana ito sa beta carotene na nagiging vitamin A para sa mas malinaw na mata at malakas na resistensya
ang bawat tasa ng Malusog Rice ay naglalaman ng beta carotene na katumbas ng 4 na tasa ng lutong kangkong
ito ay sinuring mabuti upang masiguro na ito ay ligtas kainin at hindi makakasama sa kapaligiran o ibang pananim
maaaring itabi at gamitin ang binhi ng Malusog Rice para sa susunod na taniman dahil ito ay inbred variety
ang Malusog Rice ay kayang umani ng 5 tonelada o higit pa kada ektarya
ang Malusog Rice ay kapresyo lamang ng ordinaryong bigas na mabibili sa palengke
PHILRICE
PHILIPPINE RICE RESEARCH INSTITUTE
CGIAR
IRRI
www.philrice.gov.ph /golden-rice
GoldenRicePh
GoldenRicePh
0917-111-7423

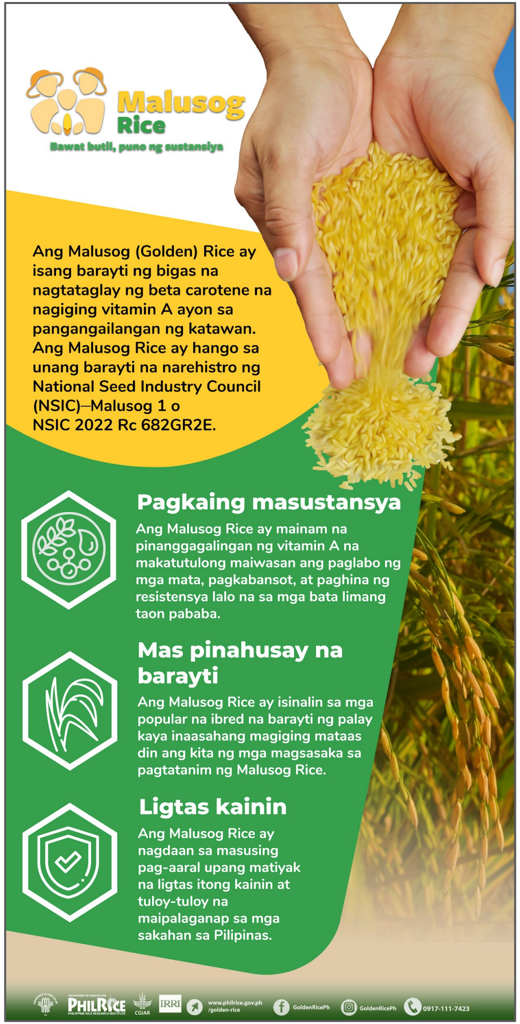
Malusog Rice
Bawat butil, puno ng sustansiya
Ang Malusog (Golden) Rice ay isang barayti ng bigas na nagtataglay ng beta carotene na nagiging vitamin A ayon sa pangangailangan ng katawan. Ang Malusog Rice ay hango sa unang barayti na narehistro ng National Seed Industry Council (NSIC)—Malusog 1 o NSIC 2022 Rc 682GR2E.
Pagkaing masustansya
Ang Malusog Rice ay mainam na pinanggagalingan ng vitamin A na makatutulong maiwasan ang paglabo ng mga mata, pagkabansot, at paghina ng resistensya lalo na sa mga bata limang taon pababa.
Mas pinahusay na barayti
Ang Malusog Rice ay isinalin sa mga popular na ibred na barayti ng palay kaya inaasahang magiging mataas din ang kita ng mga magsasaka sa pagtatanim ng Malusog Rice.
Ligtas kainin
Ang Malusog Rice ay nagdaan sa masusing pag-aaral upang matiyak na ligtas itong kainin at tuloy-tuloy na maipalaganap sa mga sakahan sa Pilipinas.
PHILRICE
CGIAR
IRRI
www.philrice.gov.ph /golden-rice
GoldenRicePh
GoldenRicePh
0917-111-7423

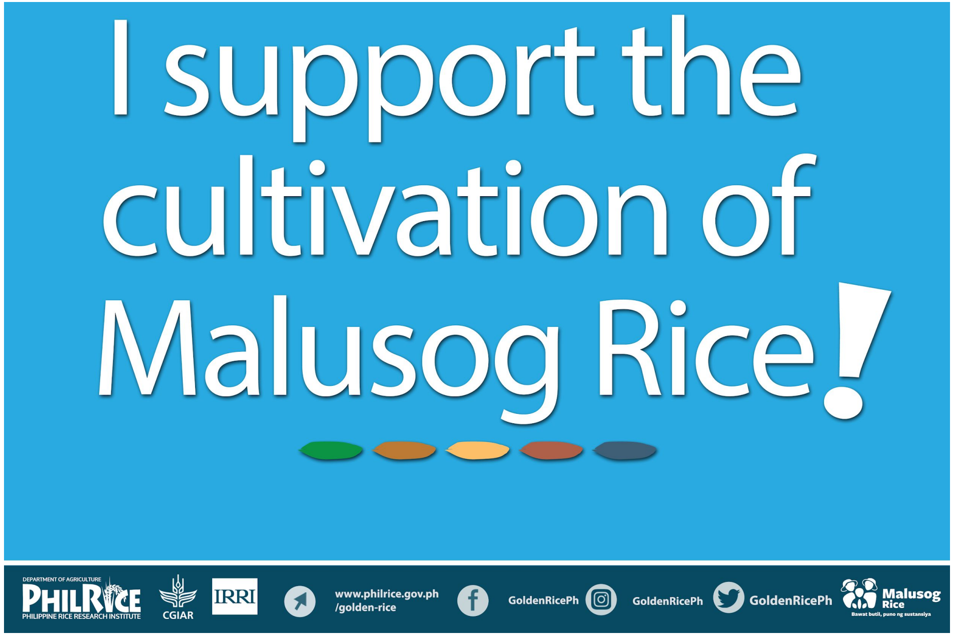
I support the cultivation of Malusog Rice!
DEPARTMENT OF AGRICULTURE
PHILRICE
PHILIPPINE RICE RESEARCH INSTITUTE
CGIAR
IRRI
www.philrice.gov.ph
/golden-rice
GoldenRicePh
GoldenRicePh
GoldenRicePh
Malusog Rice
Bawat butil, puno ng sustansiya

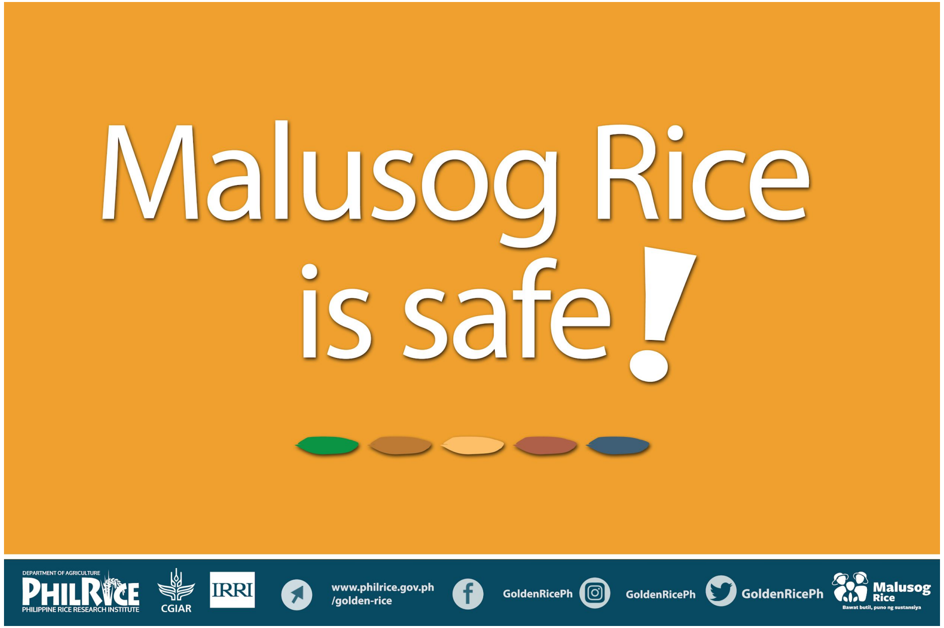
Malusog Rice is safe!
DEPARTMENT OF AGRICULTURE
PHILRICE
PHILIPPINE RICE RESEARCH INSTITUTE
CGIAR
IRRI
www.philrice.gov.ph
/golden-rice
GoldenRicePh
GoldenRicePh
GoldenRicePh
Malusog Rice
Bawat butil, puno ng sustansiya

Malusog Rice is nutritious!

DEPARTMENT OF AGRICULTURE PHILRICE PHILIPPINE RICE RESEARCH INSTITUTE | CGIAR | IRRI | www.philrice.gov.ph/golden-rice | GoldenRicePh | GoldenRicePh | GoldenRicePh | Malusog Rice Bawat butil, puno ng sustansiya

Malusog Rice will help curb vitamin A deficiency!

DEPARTMENT OF AGRICULTURE PHILRICE PHILIPPINE RICE RESEARCH INSTITUTE | CGIAR | IRRI | www.philrice.gov.ph/golden-rice | GoldenRicePh | GoldenRicePh | GoldenRicePh | Malusog Rice Bawat butil, puno ng sustansiya

Ang Golden Rice ay gaya rin ng ordinaryong bigas ngunit may beta-carotene, na siyang pinagmumulan ng vitamin A, na nagbibigay ng dilaw o mala-ginto nitong kulay. Dinebelop ang Golden Rice upang madagdagan ang vitamin A sa ating pagkain nang sa gayon ay matugunan ang suliranin sa vitamin A deficiency o VAD.

Narito ang ilang piling katanungan na madalas itanong ukol sa Golden Rice:

Gaano karaming Golden Rice ang kailangan kong kainin? Maaari ba itong makapagdulot ng masamang epekto kapag n[illegible]

Ang isang tasa ng Golden Rice [illegible] nakapagbibigay ng 30-50% vitar[illegible] pangangailangan ng mga batang [illegible] taon pababa, mga buntis at nag[illegible] ina. Ang beta carotene na taglay [illegible] Rice ay nagiging vitamin A lama[illegible] pangangailangan ng katawan ka[illegible] walang overdose o toxicity na m[illegible] sobrang pagkain nito. Anumang [illegible] beta-carotene ay ilalabas ng kat[illegible] pamamagitan ng pagdumi.

Magkaiba ba ang lasa ng Golden Rice sa ordinaryong kanin?

Base sa resulta ng mga naunang pagpapatikim ng Golden Rice, walang pagkakaiba ang lasa, amoy, at lambot nito sa ordinaryong kanin. Ibig sabihin, walang epekto ang taglay na beta-carotene ng Golden Rice sa lasa nito.

Bilang ito ay isang genetically modified crop, ligtas ba itong kainin ng mga tao?

Ang Golden Rice ay ligtas kainin, gaya ng ibang mga pagkaing genetically engineered, sapagkat ito ay dumaan sa masusing pag-aaral ng mga eksperto at [illegible]

Kailan maaaring makabili ng binhi ng Golden Rice?

Kasalukuyang isinasagawa ng DA-PhilRice at ang mga kaugnay na ahensya nito ang pagpaparami ng mga binhi ng Golden Rice matapos na aprubahan ng DA-BPI ang permiso nito para sa malawakang pagtatanim. Inaasahan na sa unang bahagi ng taong 2022 ay maaari nang maipamahagi ang Golden Rice sa mga piling probinsyang may mataas na kaso ng VAD sa pakikipagtulungan ng iba't-ibang ahensya at lokal na pamahalaan. Mas palalawakin pa ang pamamahagi ng [illegible]

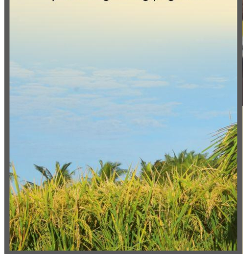

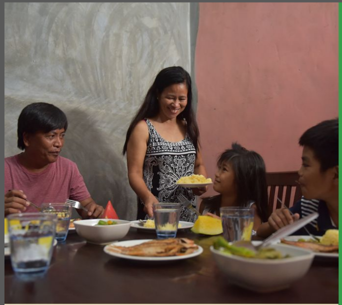

Kikita ba ang mga magsasaka sa pagtatanim ng Malusog Rice?

Ang Malusog Rice trait ay isinalin lamang sa mga paboritong inbred na barayti ng ating mga magsasaka, na kilalang may mataas na ani. Inaasahang magiging mataas din ang kita ng mga magsasaka sa pagtatanim ng Malusog Rice.

Ilang kaban ang kayang anihin sa isang ektaryang Malusog Rice?

Ang ani ng Malusog Rice ay kapareho ng ani ng barayti na pinagmulan nito; humigit-kumulang limang tonelada. Ito ay depende sa lugar, sukat ng bukid, at panahon ng pagtatanim.

Tungkol sa Programa

Ang Malusog Rice Program ay inilunsad na may layuning maihatid ang Malusog Rice sa mga magsasaka at mamamayang pilipino. Saklaw ng programa ang lahat ng mga kinakailangang proseso: mula sa pagtatanim, pagpaparami at pagpapalaganap ng binhi, pag-aani ng bigas, at pagkonsumo nito.

Layunin

Ang Malusog Rice ay nilikha upang makatulong sa pagbibigay solusyon laban sa vitamin A deficiency o kakulangan ng vitamin A sa pagkain.

Pamamaraan ng pagpapamahagi

Ang Malusog Rice ay unang ipapamahagi sa mga piling lugar na may mataas na bilang ng malnutrisyon. Ang DA-PhilRice ay makikipagtulungan sa iba't ibang ahensya mula sa nasyonal hanggang sa local government upang maisakatuparan ang layunin ng Malusog Rice na magkaroon ng sapat na suplay ng masustansya at abot-kayang bigas ang bawat mamamayang Pilipino.

Ipapamahagi ito sa mga programang pang-nutrisyon at pang-agrikultura na kasalukuyang pinapatupad ng pamahalaan. Palalawakin din ang pagpapamahagi nito sa pamilihan, farm industry players, at iba pang pang-komersyal na pamamaraan. Bibigyang prayoridad ng Malusog Rice program ang mga kabataang nakitang stunted (may pagkabansot) at mga populasyon na mahina laban sa vitamin A deficiency tulad ng mga household na nasa laylayan, mga malnourished na kabataan, mga buntis at mga nagpapasusong ina.

Para sa karagdagang impormasyon, maaaring makipag-ugnayan sa:

Malusog Rice Program
DA-Philippine Rice Research Insititute
Maligaya, Science City of Muñoz, 3119 Nueva Ecija

Scan to access Golden Rice collaterals:

Golden Rice Ph | www.philrice.gov.ph/golen-rice/about-gr | 0917-111-7423 | GoldenRicePh | prri.mail@philrice.gov.ph goldenriceph@gmail.com

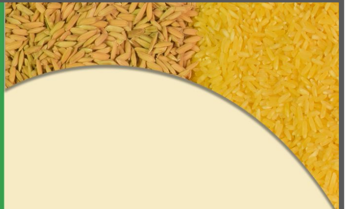

Malusog Rice

Bawat butil, puno ng sustansiya

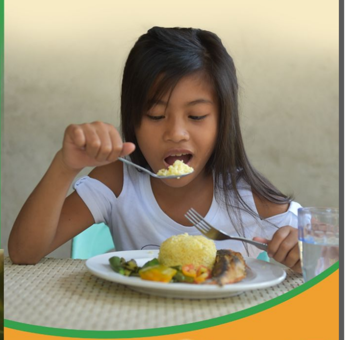

PhilRice · CGIAR · IRRI

QUESTIONS & ANSWERS

JUNE 2022

ENGLISH

Golden Rice

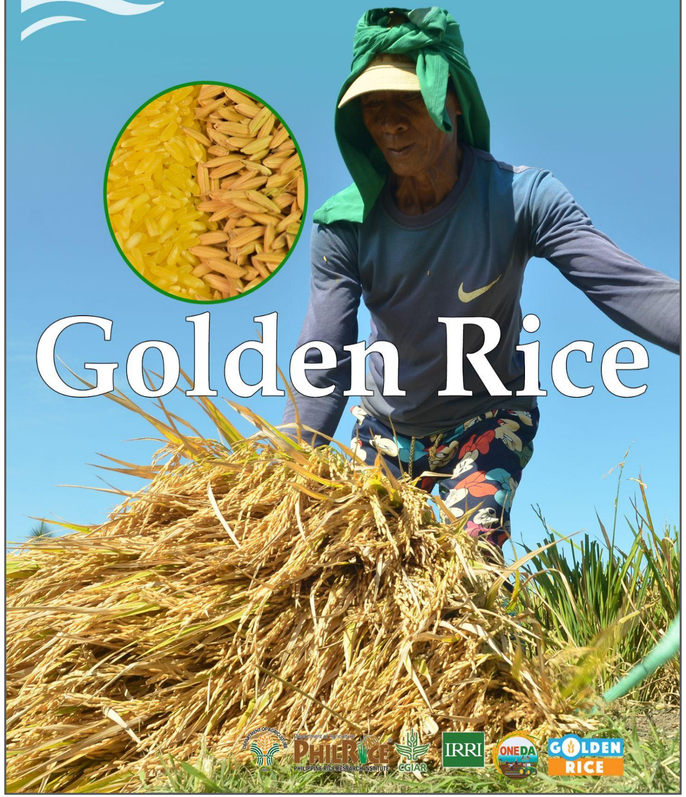

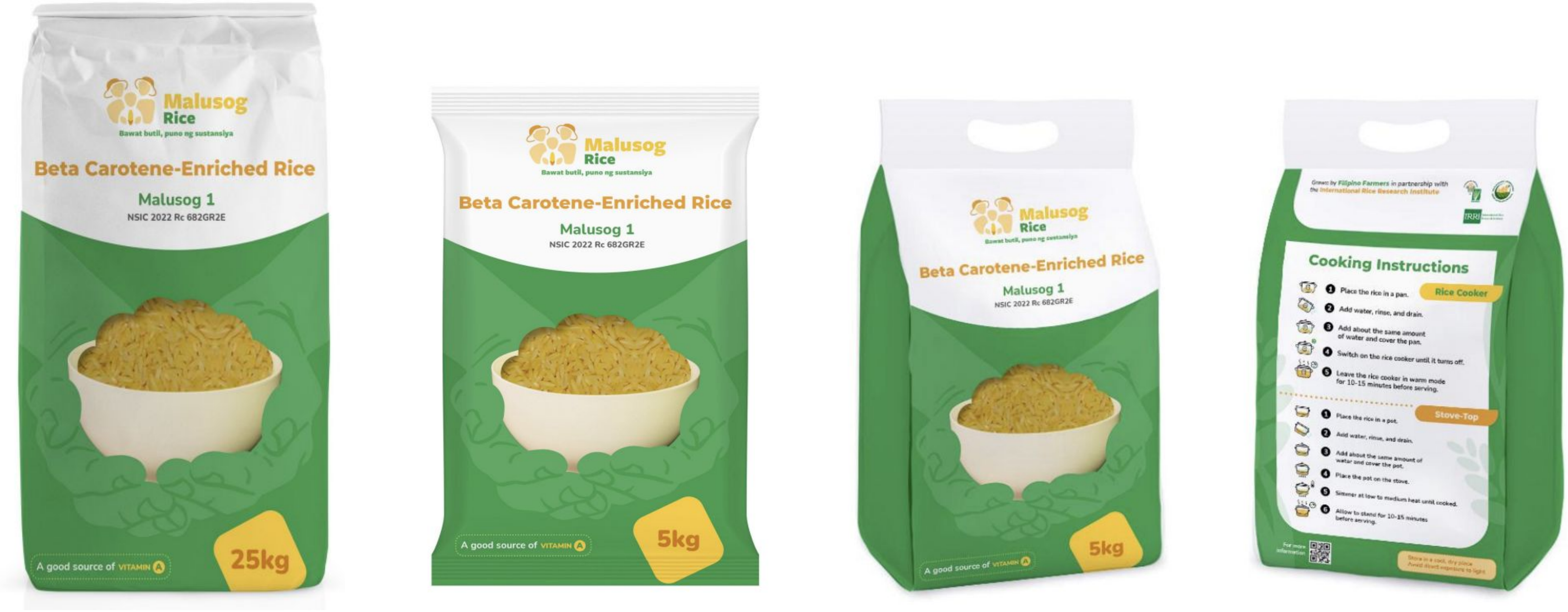
Malusog Rice
Bawat butil, puno ng sustansiya
Beta Carotene-Enriched Rice
Malusog 1
NSIC 2022 Rc 682GR2E
A good source of VITAMIN A
25kg
Malusog Rice
Bawat butil, puno ng sustansiya
Beta Carotene-Enriched Rice
Malusog 1
NSIC 2022 Rc 682GR2E
A good source of VITAMIN A
5kg
Malusog Rice
Bawat butil, puno ng sustansiya
Beta Carotene-Enriched Rice
Malusog 1
NSIC 2022 Rc 682GR2E
A good source of VITAMIN A
5kg
Grown by Filipino Farmers in partnership with the International Rice Research Institute
IRRI
Cooking Instructions
Rice Cooker
1 Place the rice in a pan.
2 Add water, rinse, and drain.
3 Add about the same amount of water and cover the pan.
4 Switch on the rice cooker until it turns off.
5 Leave the rice cooker in warm mode for 10-15 minutes before serving.
Stove-Top
1 Place the rice in a pot.
2 Add water, rinse, and drain.
3 Add about the same amount of water and cover the pot.
4 Place the pot on the stove.
5 Simmer at low to medium heat until cooked.
6 Allow to stand for 10-15 minutes before serving.
For more information
Store in a cool, dry place
Avoid direct exposure to light

Supplementary Document 15 (Malusog Rice recipes)

Malusog Rice E-Newsletter
April 2023 | Issue No. 4 of 2023
https://us2.campaign-archive.com/?u=831d5b3f7694549624621422c&id=cccc11c976

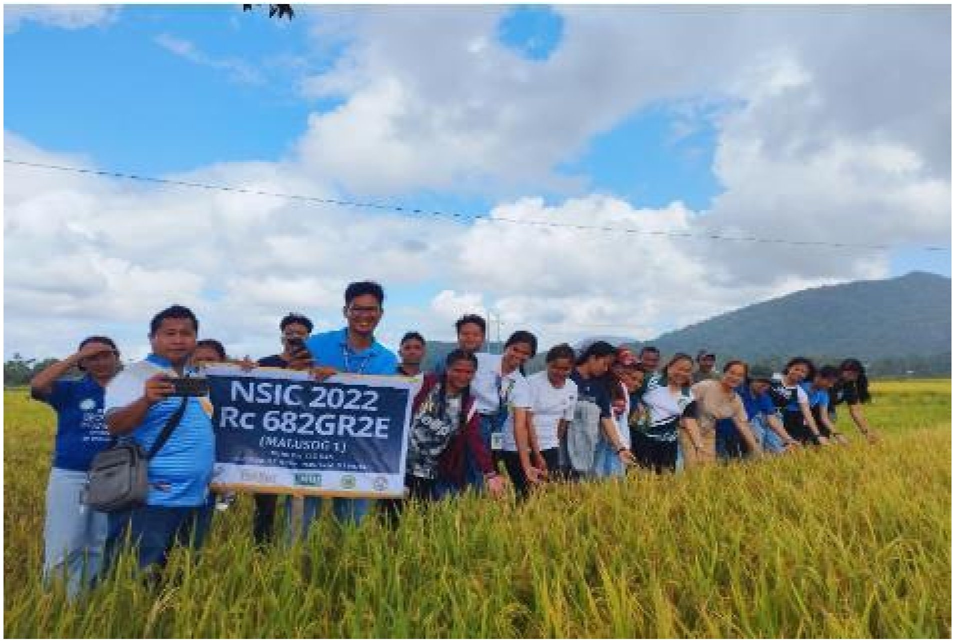

Malusog Rice farm walk held in Catanduanes

Farmers and communities in Virac and San Andres, Catanduanes received a briefing on Malusog Rice through a farm walk held on April 17 and 18, respectively.

Farmer cooperators also shared their testimonies about what encouraged them to plant Malusog Rice.

“The planting process of the Malusog Rice is just the same as the ordinary rice. I was encouraged to plant it because it would help the malnourished children in our community,” a farmer cooperator from San Andres said.

“What motivated me to plant Malusog Rice is the prevalence of malnutrition in our locality. As I learned about its beta carotene content, I was encouraged. Also, because of the Malusog Rice techno-demo, I proved and showed to the other farmers the good performance of the Malusog Rice.”

“I was encouraged to plant the Malusog Rice because according to the flyers that I have read from PhilRice, it is nutritious and so I tried it. The crop’s performance was good as well as its harvest,” shared farmer cooperators in Virac.

As of now, the harvested yield in the Virac sites is 9 metric tons from 3.82 ha (dried weight at 14% moisture content), while the total yield in San Andres is 7.5 metric tons from 6.2 ha (fresh weight at 20.5 % moisture content).

Representatives from the Office of the Provincial Agriculturist (OPAG), Municipal Agriculturist Office (MAO), and Municipal Nutrition Action Office (MNAO) were also present. They are still committed as members of the Provincial Technical Working Members (PTWG) of the program.

“As Municipal Nutrition Action Officer, we can encourage the public to promote Malusog Rice through advocacy campaigns and endorse it to the local barangays for their respective feeding activities. We need to support the Malusog Rice Program because of its objective in helping address VAD in our municipality. Our children as well as the pregnant and lactating mothers will greatly benefit from it,” MNAO Virac Jocelyn Quinones, RND said.

See Facebook post here:

Supplementary Document 16 (list of press releases on Malusog Rice)

News articles on Malusog Rice

1. DA-PhilRice introduces Golden Rice
   Philippine Information Agency
   August 17, 2022

2. Sibalom farmer reaps his first Golden Rice grains
   Province of Antique Facebook Page
   October 18, 2022

3. Malusog Rice tastes like…rice
   BusinessMirror
   November 27, 2022

4. Malusog Rice harvested
   Philippine Rice Research Institute (PhilRice)
   December 28, 2022

5. 100 tons of Golden Rice harvested in 17 sites, including Catanduanes
   Catanduanes Tribune
   January 11, 2023

6. Malusog Rice to address malnutrition in Catanduanes
   DA-PhilRice
   January 20, 2023

7. Successful ‘Malusog Rice’ production celebrated in Antique
   Philippine Information Agency
   March 8, 2023

8. Malusog Rice propagation in Antique gets provincial government support
   DA Western Visayas (Antique RFO)
   April 4, 2023

9. Malusog Rice promises healthy harvest in Catanduanes
   DA Regional Field Office 5 (Bicol)
   March 4, 2023

Supplementary Document 17 (Malusog Rice recipes)

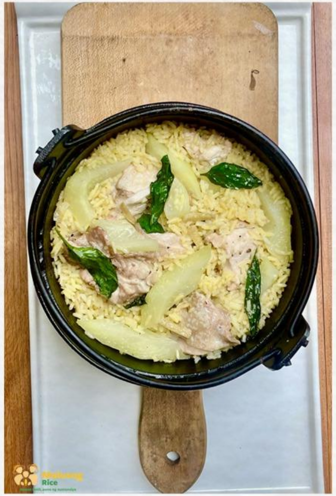

Chicken Tinola Malusog Rice Pot

*Malusog (Golden) Rice is a new type of rice containing beta carotene, which can provide 30-50% estimated average requirement for vitamin A of young children.

Ingredients: Makes 6 servings

- 30 ml vegetable oil
- 50 g sliced ginger
- 10 g garlic
- 500 g boneless chicken thigh
- 360 g Malusog Rice
- 720 ml water or chicken stock
- 150 g green papaya
- 30 ml fish sauce
- 5 g iodized salt
- 5 g ground black pepper
- 5 g chili leaves

Directions:

1. In a lage pot, heat oil, sauté ginger and garlic until fragrant.
2. Add chicken fillets and cook for 3 minutes until it changes color. Set aside.
3. Put the Malusog Rice in to the pot. Mix well.
4. Pour in broth, and fish sauce.
5. Arrange cooked chicken and papaya on top of the rice.
6. Season with salt and pepper.
7. Cover the pot and cook until rice is almost done.
8. Top with chili leaves and cover the pot to finish cooking.
9. Serve hot.

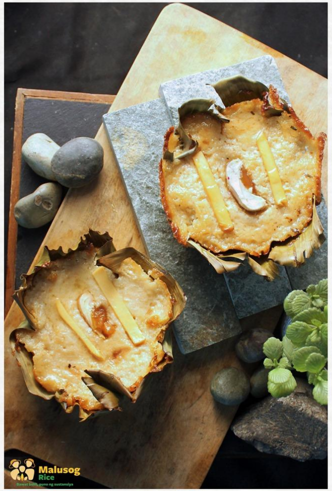

Malusog Rice Bibingka

*Malusog (Golden) Rice is a new type of rice containing beta carotene, which can provide 30-50% estimated average requirement for vitamin A of young children.

Ingredients: Makes 4 bibingkas

- 1 cup Malusog Rice, ground into flour
- 1.5 tsp baking powder
- 1 tsp iodized salt
- 1/2 cup white sugar
- 1 pc salted egg
- 3 pcs eggs
- 1.5 cup coconut milk
- 1/2 cup evaporated milk
- 3 tbsp melted vegetable margarine
- 1/2 cup cheddar cheese
- 1/4 cup grated coconut
- 1 pc banana leaf

Directions:

1. Preheat oven to 375° Fahrenheit.
2. Combine rice flour, baking powder, and salt then mix well. Set aside.
3. Cream butter then gradually put in sugar while whisking.
4. Add the eggs then whisk until every ingredient is well incorporated.
5. Gradually add the rice flour, salt, and baking powder mixture then continue mixing.
6. Pour in coconut milk and fresh milk then whisk some more for 1 to 2 minutes.
7. Arrange the pre-cut banana leaf on a cake pan or baking pan.
8. Pour the mixture on the pan.
9. Bake for 15 minutes.
10. Remove from the oven then top with sliced salted egg and grated cheese (do not turn the oven off).
11. Put back in the oven and bake for 15 to 20 minutes or until the color of the top turns medium brown.
12. Remove from the oven and let cool.
13. Brush with butter and top with grated coconut.

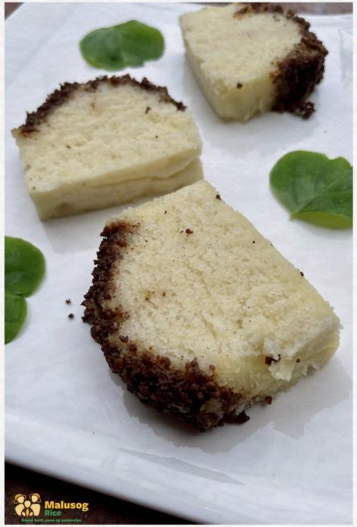

Latik Malusog Rice Puto Loaf

*Malusog (Golden) Rice is a new type of rice containing beta carotene, which can provide 30-50% estimated average requirement for vitamin A of young children.

Ingredients: (Makes 6 servings)

- 480 g coconut cream
- 160 g Malusog Rice
- 2 g iodized salt
- 120 g white sugar
- 6 g baking powder
- 6 g instant yeast
- 182 ml coconut milk
- 160 ml hot water

Directions:

1. Pour coconut cream on a medium sauce pan;
2. Boil until coconut cream curds. Strain and set aside;
3. Grind malusog rice until almost powdered;
4. Combine all dry ingredients in a large mixing bowl;
5. Mix coconut milk, hot water and oil to make it lukewarm. You can also combine both and heat to lukewarm. Add this to the dry ingredients;
6. Let it rest for 10 minutes and then gently mix several times until it becomes thicker;
7. Transfer the mixture to oiled or lined with banana leaves small loaf pans and let it rest for another 20 minutes;
8. Sprinkle latik on top of the mixture;
9. Boil water in steamer then reduce to simmer;
10. Arrange loaf pans on the steamer basket and cover with the lid lined with cloth. 11. Steam for 25-40 minutes or until inserted toothpick comes out clean;
12. Remove the mold from heat and let it cool down a bit before unmolding.

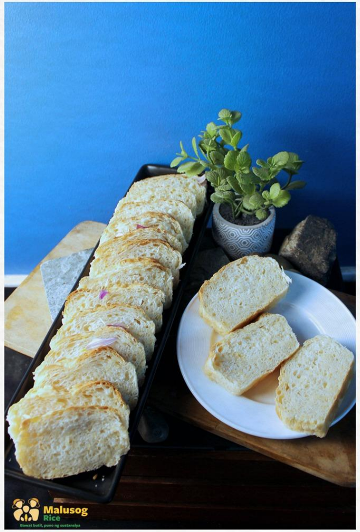

Malusog Rice Bread

*Malusog (Golden) Rice is a new type of rice containing beta carotene, which can provide 30-50% estimated average requirement for vitamin A of young children.

Ingredients: Makes 6 loaves of bread

- 390 g bread flour
- 360 g all-purpose flour
- 16 g instant yeast
- 240 ml water
- 240 ml full fresh milk
- 60 g cubed vegetable margarine
- 3 cups cooked Malusog Rice

Directions:

1. In a large bowl, combine bread flour, all-purpose flour, yeast, and salt.
2. In a bowl, heat combined water, milk, and sugar. Mix well.
3. Add liquid to dry ingredients; knead until formed. Add butter and continue kneading for 10 minutes.
4. Turn onto a lightly floured surface; knead until smooth and elastic, about 6-8 minutes.
5. Add in Malusog Rice. Continue kneading to form a stiff dough.
6. Place dough in a bowl coated with oil, turning once to coat top.
7. Cover and let rise in a warm place until doubled, about 30-40 minutes.
8. Punch dough down. Turn onto a lightly floured surface; divide into 6. Shape into loaves. Place in 23.5cm x 9cm loaf pans coated with cooking spray. Cover and let rise until doubled, about 30 minutes.
9. Bake at 375° for 40-45 minutes or until golden brown. Remove from pans and place in wire racks to cool completely.

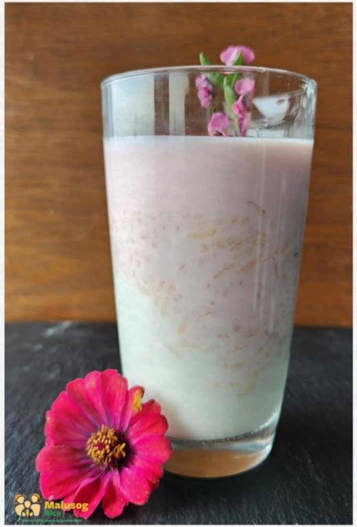

Malusog Iskrambol Rice Drink

*Malusog (Golden) Rice is a new type of rice containing beta carotene, which can provide 30-50% estimated average requirement for vitamin A of young children.

Ingredients: Makes 10 servings

- 187 g Malusog Rice, soaked in 4 cups water with cinnamon bark
- 0.6 l water
- 213 ml evaporated milk
- 250 ml condensed milk
- 93 g white sugar
- 1 ml red food coloring
- 62.5 g lakatan banana or 2.5 ml banana extract
- 50 g powdered sugar

Directions:

1. Puree Malusog Rice, cinnamon bark and soaking water until completely ground.
2. Strain mixture thru a fine sieve, transfer in a blender.
3. Add evaporated milk, condensed milk, white sugar, powdered milk, red food color, and bananas. Puree well
4. Transfer to serving glasses with crushed ice and sprinkle powdered milk on top.

OPTIONAL: Garnish with marshmallow, cooked Malusog Rice, and chocolate syrup.

PHILRICE PHILIPPINE RICE RESEARCH INSTITUTE CGIAR IRRI www.philrice.gov.ph /golden-rice GoldenRicePh GoldenRicePh GoldenRicePh CCA

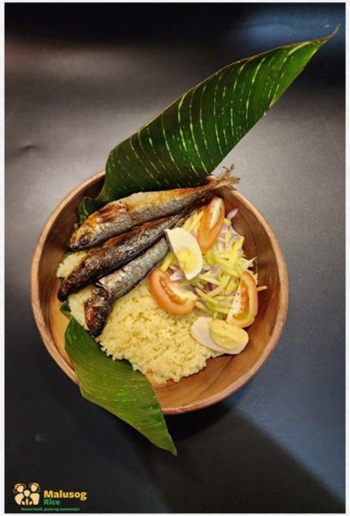

Tinapa, Mangga, Itlog na maalat at Malusog Rice sinangag

*Malusog (Golden) Rice is a new type of rice containing beta carotene, which can provide 30-50% estimated average requirement for vitamin A of young children.

Ingredients: Makes 6 servings

- 80 ml canola oil
- 250 g tinapang galunggong
- 400 g cooked Malusog Rice
- 50 g garlic
- 5 g iodized salt
- 200 g green mango, cut into strips
- 3 pcs salted egg, cut into wedges
- 100 g tomato, cut into wedges
- 60 g red onion, cut into thin strips

Directions:

1. Heat canola oil in a pan;
2. Fry tinapa and set aside;
3. Using the same pan and oil, lightly toast minced galic;
4. Add cooked Malusog Rice and saute, season with salt;
5. Place in a serving bowls, put the fried tinapa on top of rice;
6. Combine mangoes and onions, place beside the tinapa;
7. Serve tinapa rice bowl with sliced tomatoes and salted eggs.

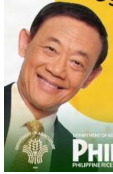

PHILRICE PHILIPPINE RICE RESEARCH INSTITUTE CGIAR IRRI www.philrice.gov.ph /golden-rice GoldenRicePh GoldenRicePh GoldenRicePh CCA

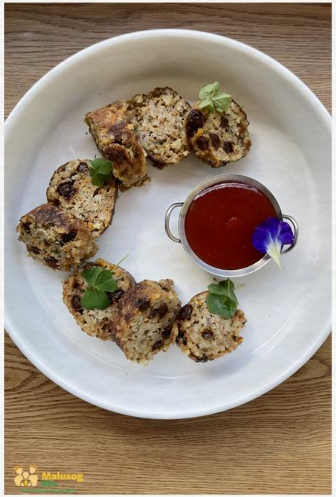

Malusog Rice Embutido

*Malusog (Golden) Rice is a new type of rice containing beta carotene, which can provide 30-50% estimated average requirement for vitamin A of young children.

Ingredients: Makes 6 servings

- 90 g Malusog Rice
- 250 g ground pork
- 2 pcs egg
- 30 g all-purpose flour
- 100 g grated carrots
- 30g mined white onion
- 80 g sweet pickle relish
- 30 g tomato ketchup
- 360 g raisins
- 5 g iodozed salt
- 5 g ground black pepper

Directions:

1. Cook the Malusog Rice in water. Let it cool.
2. Put all ingredients in a large bowl.
3. Mix everything until well incorporated. Set aside.
4. In an aluminum foil, place half of the meat mixture.
5. Roll the foil so that the meat forms a cylinder or log.
6. Twist the ends of the foil to lock.
7. Steam for 45 to 60 minutes.

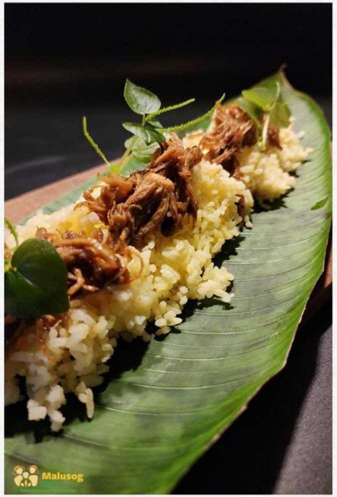

Chicken Pastil at Malusog Rice

*Malusog (Golden) Rice is a new type of rice containing beta carotene, which can provide 30-50% estimated average requirement for vitamin A of young children.

Ingredients: Makes 6 servings

- 1 L Water
- 5 g iodized salt
- 2 pcs bay leaf
- 300 g Malusog Rice
- 45 g garlic
- 60 ml canola oil
- 1 pc red onion
- 350 g chicken breast
- 80 ml soy sauce
- 45 ml white wine vinegar
- 2.5 g ground black pepper
- 30g margarine
- 10 g brown sugar

Directions:

CHICKEN
1. In a pot add 1 liter of water, 1 tsp salt and 1 tsp black pepper & 2 bay leaves;
2. Add 350g chicken breast fillet;
3. Boil on medium heat for about 15 mins;
4. Drain, let it cool down & shred the chicken;
5. Set aside boiling liquid.

MALUSOG RICE
1. Combine malusog rice and 2 1/2 cups of reserved chicken boiling liquid;
2. Bring to boil uncovered. Turn the heat down and simmer. Cover the pot and cook the rice over very low heat

PASTIL
1. Heat oil in a pan. Add minced garlic and minced red onion
2. Fry until translucent then add 350g shredded chicken and saute until light brown in color
3. Season with black pepper and sugar. Add soy sauce & bring to boil. Add vinegar and let it simmer
4. Add 2 tbsp margarine and serve on top of hot rice.

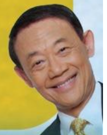

DEPARTMENT OF AGRICULTURE PHILRICE PHILIPPINE RICE RESEARCH INSTITUTE CGIAR IRRI www.philrice.gov.ph /golden-rice GoldenRicePh GoldenRicePh GoldenRicePh CCA Center for Culinary Arts, Manila

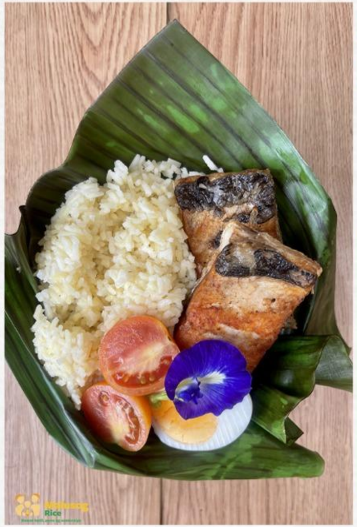

Daing na Bangus Binalot

*Malusog (Golden) Rice is a new type of rice containing beta carotene, which can provide 30-50% estimated average requirement for vitamin A of young children.

Ingredients: (Makes 6 servings)

- 500 g bangus belly
- 240 ml vinegar
- 30 g crushed garlic
- 5 g peppercorn
- 2 pcs banana leaves
- 630 g cooked Malusog Rice
- 3 pcs tomato (cut in half)
- 3 pcs hard boiled eggs (cut in half)

Direction:

1. Marinate bangus belly in vinegar, garlic and peppercorns at least overnight;
2. Preheat oil in a pan then fry bangus until golden brown;
3. Lay banana leaves on a clean counter or plate;
4. Scoop malusog rice then top with tomatoes and hard-boiled eggs;
5. Wrap the leaves tightly.

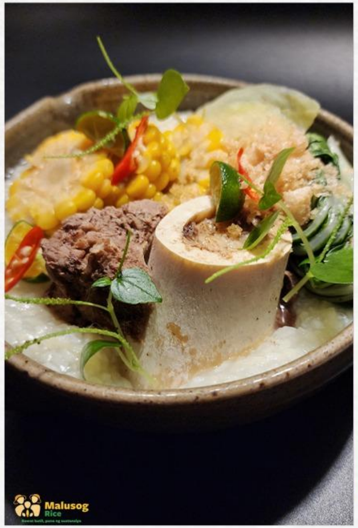

Malusog Bulalugaw

*Malusog (Golden) Rice is a new type of rice containing beta carotene, which can provide 30-50% estimated average requirement for vitamin A of young children.

Ingredients: Makes 10 servings

- 1 kg beef shank, nilaga cut
- 3 l beef stock
- 120 g glutinous rice
- 220 g Malusog Rice
- 500 g sweet corn
- 60 g garlic
- 200 g red onion
- 80 g pechay Baguio cut into segmets
- 80 g Chinese pechay cut into segments
- 100 g cabbage cut into wedges
- 5 g black ground pepper
- 60 ml fish sauce
- 60 g calamansi
- 40 g ground chicharon
- 20 g green onion
- 5 g red chili pepper

Directions:

1. Combine beef stock, patis, black pepper, beef shank, corn, onions, and garlic in a pot, then cover. Bring to boil and simmer until meat is tender. Add vegetables and continue cooking until vegetables are cooked. Strain all ingredients and set aside.
2. Bring remaining simmering liquid to a boil. Add Malusog Rice and glutinous rice and cook until thick and rice is tender.
3. Add strained meat and vegetables to cooked rice porridge.
4. Garnish with chicharon, calamnsi, green onions, and red chili if desired.

Supplementary Document 18 (stakeholder’s feedback on sensory characteristics of Malusog Rice)

<table>
<tr><th></th><th>Stakeholder</th><th>Testimony</th><th>Source</th></tr>
<tr><td>1</td><td>Cecile Ibayan from Barangay Buyo, Virac,</td><td>“It was not difficult for me to feed my children the Malusog Rice because they really liked it, and I am satisfied because it is more nutritious. I approve of the Malusog Rice!”</td><td>Article title: Malusog Rice distributed in Catanduanes

Golden Rice E-Newsletter December 2022 | Issue No. 12 of 2022

https://us2.campaign-archive.com/?u=831d5b3f7694549624621422c&id=ac3ae4efa3</td></tr>
<tr><td>2</td><td>Rocky Mabborang, project manager and marketing officer of the Cagayan Seed Producers Multipurpose Cooperative, Cagayan</td><td>“It’s surprisingly tasteful. It also smells like ordinary well milled rice,”</td><td rowspan="2">Article title: Malusog Rice gains positive feedback at Agri Summit in Cagayan

Malusog Rice E-Newsletter April 2023 | Issue No. 4 of 2023

https://us2.campaign-archive.com/?u=831d5b3f7694549624621422c&id=cccc11c976</td></tr>
<tr><td>3</td><td>Ian Soliven, science research technician from Cagayan Valley Research Center, Cagayan</td><td>“It’s palatable and soft in texture. I’m sure kids will love it.”</td></tr>
<tr><td>4</td><td>Farmer Purification Tabo from Catanduanes</td><td>"It is just the same as the white rice, it does not have any distinct smell. Even if I mix it with the white rice, it tastes the same. Of course, I will encourage other families to eat Malusog Rice, especially if it is cheaper."</td><td>Article title: Taste buds agree: Malusog Rice excels in taste tests in various regions

Malusog Rice E-Newsletter May 2023 | Issue No. 5 of 2023

https://us2.campaign-archive.com/?u=831d5b3f7694549624621422c&id=87388f7f1a</td></tr>
</table>

5	Vice Governor Samuel E. Tortor and 13 Sangguniang Panlalawigan members	"In Agusan del Sur, Vice Governor Samuel E. Tortor and 13 Sangguniang Panlalawigan members were also able to taste Malusog Rice in their regular session on May 8. According to the members of SP, they liked the taste of Malusog Rice as it was just the same as ordinary rice."	Malusog Rice E-Newsletter May 2023 | Issue No. 5 of 2023<br><br>Taste buds agree: Malusog Rice excels in taste tests in various regions<br><br>https://us2.campaign-archive.com/?u=831d5b3f7694549624621422c&id=87388f7f1a
6	Participants of Training of Trainers (ToT) on Production of High-Quality Inbred Rice Seeds and Farm Mechanization from DA-PhilRice Batac on May 24.	Most of them were also impressed that Malusog Rice has "good eating quality" and has its added nutritional value.	

Supplementary Document 19 (documentation of stakeholder support for Malusog Rice, as reported in the Malusog Rice newsletter)

	Stakeholder	Testimony	Source
1	Municipal Nutrition Action Officer, Virac, Catanduanes	“As Municipal Nutrition Action Officer, we can encourage the public to promote Malusog Rice through advocacy campaigns and endorse it to the local barangays for their respective feeding activities. We need to support the Malusog Rice Program because of its objective in helping address VAD in our municipality.	Article title: Malusog Rice farm walk held in Catanduanes<br><br>Malusog Rice E-Newsletter April 2023 | Issue No. 4 of 2023<br><br>https://us2.campaign-archive.com/?u=831d5b3f7694549624621422c&id=cccc11c976
2	Mayor, Balungao, Pangasinan	“As we see it, Malusog Rice can be a big help in providing nutritional value in the diet especially for children. This would be a great advantage if they would incorporate it in their diet.”	Article title: Second planting of Malusog Rice reaps 8.3t/ha this May, new yield record set<br><br>Malusog Rice E-Newsletter May 2023 | Issue No. 5 of 2023<br><br>https://us2.campaign-archive.com/?u=831d5b3f7694549624621422c&id=87388f7f1a

<table>
<tr><td>3</td><td>Former Mayor Mr. Eddie Guillen, Piddig, Ilocos Norte</td><td>“Mr. Guillien mentioned that Golden Rice can become part of their First 1000 Days Program where pregnant and lactating mothers as well as their older babies can fully benefit from this innovation.”</td><td>Article title: Ilocos Norte and Pangasinan Golden Rice production areas in full swing

Golden Rice E-Newsletter September 2022 | Issue No. 9 of 2022

https://us2.campaign-archive.com/?u=831d5b3f7694549624621422c&id=aa7b259033</td></tr>
<tr><td>4</td><td>National Food Authority Region 5</td><td>“This is to ensure that farmer cooperators receive a fair price for their crop, which encourages them to grow more of the Malusog Rice.”

“The purchased rice will then be procured by the Provincial Social Welfare and Development in Catanduanes to be distributed to the target communities in need of additional sources of vitamin A.”</td><td>Article title: Malusog Rice program-based deployment approach at work in Catanduanes

Malusog Rice E-Newsletter April 2023 | Issue No. 4 of 2023

https://us2.campaign-archive.com/?u=831d5b3f7694549624621422c&id=cccc11c976</td></tr>
<tr><td>5</td><td>Governor Rhodora Cadiao of Antique</td><td>“As a mother who wants to provide proper nourishment to my children, I want them to eat and get the benefits of Malusog Rice.”</td><td rowspan="2">Article title: Malusog Rice program continues to gain support of local chief executives

Malusog Rice E-Newsletter June 2023 | Issue No. 6 of 2023

https://us2.campaign-archive.com/?u=831d5b3f7694549624621</td></tr>
<tr><td>6</td><td>Governor Fredenil Hernaez Castro of Capiz</td><td>“Governor Hernaez, on the other hand, said he wants to plant Malusog Rice himself in support of the program. He stated specifically that he will use 1 hectare of his own farm land for the production.”</td></tr>
</table>

			422c&id=3e578ded7a
7	Vice Mayor Eric Rey G. Siohan of Esperanza,Agusan del Sur.	"I hope that next year, after consuming Malusog Rice, our children will be healthy, and this municipality will no longer be the third in the municipalities that have the highest number of malnutrition cases. I also encourage the MSWD to use Malusog Rice in their supplemental feeding program," Vice Mayor Siohan said.	Article title: Esperanza, Agusan del Sur says YES to Malusog Rice<br><br>Malusog Rice E-Newsletter June 2023 | Issue No. 6 of 2023<br><br>https://us2.campaign-archive.com/?u=831d5b3f7694549624621422c&id=3e578ded7a
8	2nd District Rep. Mark Cojuancgo, Pangasinan	“I’ve been advocating the release of Malusog Rice in the Philippines for over twenty years now as children especially in poor families do not meet the nutrients they need in the daily diet,” Cojuangco said.<br><br>“Children with VAD are stunted, have poor brain development, and weak immune systems. That's why there’s a need for Malusog Rice to be propagated to help us address these health concerns,” he added.	Article title: Malusog Rice to be integrated in Pangasinan rice relief program<br><br>Malusog Rice E-Newsletter June 2023 | Issue No. 6 of 2023<br><br>https://us2.campaign-archive.com/?u=831d5b3f7694549624621422c&id=3e578ded7a
